# Drug Metabolizing Enzyme and Transporter Gene Variation, Nicotine Metabolism, Prospective Abstinence, and Cigarette Consumption

**DOI:** 10.1371/journal.pone.0126113

**Published:** 2015-07-01

**Authors:** Andrew W. Bergen, Martha Michel, Denise Nishita, Ruth Krasnow, Harold S. Javitz, Karen N. Conneely, Christina N. Lessov-Schlaggar, Hyman Hops, Andy Z. X. Zhu, James W. Baurley, Jennifer B. McClure, Sharon M. Hall, Timothy B. Baker, David V. Conti, Neal L. Benowitz, Caryn Lerman, Rachel F. Tyndale, Gary E. Swan, Transdisciplinary Research in Cancer of the Lung Research Team

**Affiliations:** 1 Center for Health Sciences, SRI International, Menlo Park, California, United States of America; 2 Academic Research Systems, University of California San Francisco, San Francisco, California, United States of America; 3 Department of Human Genetics, Emory University School of Medicine, Atlanta, Georgia, United States of America; 4 Department of Psychiatry, Washington University School of Medicine, St. Louis, Missouri, United States of America; 5 Oregon Research Institute, Eugene, Oregon, United States of America; 6 Department of Pharmacology and Toxicology, University of Toronto, Toronto, Ontario, Canada; 7 BioRealm, LLC, Monument, Colorado, United States of America; 8 Group Health Research Institute, Seattle, Washington, United States of America; 9 Department of Psychiatry, University of California San Francisco, San Francisco, California, United States of America; 10 Center for Tobacco Research and Intervention, Department of Medicine, University of Wisconsin School of Medicine and Public Health, Madison, Wisconsin, United States of America; 11 Department of Preventive Medicine, University of Southern California, Los Angeles, California, United States of America; 12 Departments of Medicine and of Bioengineering & Therapeutic Sciences, University of California San Francisco, San Francisco, California, United States of America; 13 Department of Psychiatry, University of Pennsylvania, Philadelphia, Pennsylvania, United States of America; 14 Cambell Family Mental Health Research Institute, Centre for Addiction and Mental Health, and Departments of Psychiatry, and of Pharmacology and Toxicology, University of Toronto, Toronto, Ontario, Canada; 15 Stanford Prevention Research Center, Department of Medicine, Stanford University School of Medicine, Palo Alto, California, United States of America; National Cancer Institute, National Institutes of Health, UNITED STATES

## Abstract

The Nicotine Metabolite Ratio (NMR, ratio of *trans*-3’-hydroxycotinine and cotinine), has previously been associated with CYP2A6 activity, response to smoking cessation treatments, and cigarette consumption. We searched for drug metabolizing enzyme and transporter (DMET) gene variation associated with the NMR and prospective abstinence in 2,946 participants of laboratory studies of nicotine metabolism and of clinical trials of smoking cessation therapies. Stage I was a meta-analysis of the association of 507 common single nucleotide polymorphisms (SNPs) at 173 DMET genes with the NMR in 449 participants of two laboratory studies. Nominally significant associations were identified in ten genes after adjustment for intragenic SNPs; *CYP2A6* and two *CYP2A6* SNPs attained experiment-wide significance adjusted for correlated SNPs (*CYP2A6 P*_ACT_=4.1E-7, rs4803381 *P*_ACT_=4.5E-5, rs1137115, *P*_ACT_=1.2E-3). Stage II was mega-regression analyses of 10 DMET SNPs with pretreatment NMR and prospective abstinence in up to 2,497 participants from eight trials. rs4803381 and rs1137115 SNPs were associated with pretreatment NMR at genome-wide significance. In *post-hoc* analyses of *CYP2A6* SNPs, we observed nominally significant association with: abstinence in one pharmacotherapy arm; cigarette consumption among all trial participants; and lung cancer in four case:control studies. *CYP2A6* minor alleles were associated with reduced NMR, CPD, and lung cancer risk. We confirmed the major role that CYP2A6 plays in nicotine metabolism, and made novel findings with respect to genome-wide significance and associations with CPD, abstinence and lung cancer risk. Additional multivariate analyses with patient variables and genetic modeling will improve prediction of nicotine metabolism, disease risk and smoking cessation treatment prognosis.

## Introduction

The predominant enzyme involved in nicotine and cotinine metabolism is CYP2A6 [1, 2], where nicotine is metabolized primarily to cotinine, and cotinine primarily to *trans*-3’-hydroxycotinine. The Nicotine Metabolite Ratio (NMR) is the ratio of the metabolites *trans*-3’-hydroxycotinine and cotinine. The ratio reflects the enzymatic activity of CYP2A6 and is a biomarker of the rate of nicotine clearance [3]. The ratio is associated with smoking topography [4], responsiveness to smoking cessation treatments [5], and the number of cigarettes smoked per day (CPD) [6]. Significant correlations between the NMR and a) oral clearance of nicotine, b) oral clearance and *t*_1/2_ of cotinine, and c) lack of production of *trans*-3’-hydroxycotinine in individuals homozygous for null *CYP2A6* alleles, support the validity of the NMR as a marker of CYP2A6 activity [3]. In addition to *CYP2A6*, genetic and biochemical studies have identified contributions of additional DMET loci to measures of nicotine metabolism in diverse samples and study designs. Gene variants associated with differences in nicotine metabolism [7] influence smoking behavior, tobacco exposures and attributable disease risks, and could serve as biomarkers for disease risk, and treatment prognosis [8].

To discover and develop novel biomarkers of nicotine metabolism and related phenotypes, we performed a planned analysis to: a) identify DMET SNPs associated with the laboratory study-based NMR [9, 10] using the DMET Plus Array [11]; b) validate nominally significant DMET SNP associations with baseline NMR in clinical trial participants from two trials [12]; and, c) further validate nominally significant DMET SNPs with end-of-treatment and six-month seven day point prevalence abstinence (abstinence) in clinical trial participants from eight trials [12–18]. We also conducted *post-hoc* analyses of validated DMET SNPs in clinical trial participants with abstinence by smoking cessation pharmacotherapy assignment, and with nicotine dependence measures among all participants. Finally, we interrogated a meta-GWAS lung cancer database [19] for association results of two validated DMET SNPs.

## Methods

### Human Subjects

Institutional Review Board approval for each study, and informed written consent from each participant, was obtained by the Principal Investigators of each study. Institutional Review Board approval for these analyses was obtained from the Committee on Human Research at the University of California San Francisco and the Human Subjects Committee at SRI International, and for the TRICL study by the institutional ethics review committees at the involved institutions.

### Stage I Participants

Stage I participants were derived from two distinct studies (Table 1). Healthy twin pairs and siblings were recruited from the Northern California Twin Registry, age ≥18 and <65 years, <130% of height-adjusted ideal weight, neither pregnant nor intending to become pregnant, and after exclusion of chronic medical and psychiatric conditions (PKTWIN study) [9]. Participants underwent a 30 minute infusion of deuterium-labeled nicotine (3’,3’-dideuteronicotine) and cotinine (2’,4’,5’,6’-tetradeuterocotinine), monitoring, and blood and urine collection in a hospital setting [9]. Probands and two first-degree relatives from 158 pedigrees with ≥three ever-smoking individuals per pedigree were recruited to assess the relations between genetic factors, environmental factors and tobacco use (SMOFAM study) [10]. Individuals from 61 pedigrees completed a clinical study of nicotine metabolism; oral administration of a fixed dose of deuterium-labeled nicotine and cotinine at home, monitored by a nurse, was followed by collection, aliquoting and freezing of saliva samples at multiple time points [10]. Participants completed a detailed questionnaire and provided a blood sample for DNA extraction and analysis [10]. Levels of nicotine, cotinine *trans*-3’-hydroxycotinine and their glucuronides were estimated via liquid chromatography-mass spectrometry and pharmacokinetic phenotypes calculated, as described [3], from each study.

**Table 1:** Laboratory study participant characteristics.

Dataset		PKTWIN	SMOFAM
N individuals		247	202
N families		120	59
Age, Years, mean (SD)	Overall	247 (37.5, 12.7)	202 (39.7, 13.8)
	Twins	235 (37.2, 12.6)	
	Siblings	12 (43.7, 13.2)	
	Offspring		113 (28.1, 4.6)
	Parents		89 (54.4, 4.3)
Sex (N, %)^a^	Female	178 (72.1%)	107 (53.0%)
Smoking status (N, %)^a^	Current	44 (17.8%)	70 (34.7%)
	Former	62 (25.1%)	66 (32.7%)
	Never	141 (57.1%)	66 (32.7%)
NMR (mean, SD)	VA, PA^b^	247 (0.27, 0.14)	
	^c^OA, SA		202 (0.28, 0.14)
BMI (kg/m^2^)^a^		247 (25.1, 4.8)	202 (28.6, 6.78)
Zygosity	MZ	188 (80.0%)	
Menopausal status	Pre-menopausal	130 (77.4%)	
	During menopause	18 (10.7%)	
	Post-menopausal	20 (11.9%)	
Hormone use	Oral Contraceptive	40 (25.8%)	
	^d^HRT	16 (10.3%)	

^a^ *P*<0.001 for *t*, *χ* ^2^, and *t* tests, respectively.

^b^Venous administration, plasma analysis.

^c^Oral administration, saliva analysis.

^d^Hormone Replacement Therapy.

### Stage I Analysis

Genomic DNA was extracted from whole blood [20], and quantified [21]. DNA samples were genotyped using the Affymetrix DMET Plus Array [11] (S1 File). Genotype and phenotype data are accessible through application to a data access committee using dbGaP Study ID phs000931.v1.p1. We performed association analyses with common biallelic SNPs and indels [≥0.05 minor allele frequency (MAF) in each dataset] with the NMR derived from the six hour biospecimen collection. In the PKTWIN dataset, the NMR was square-root transformed and adjusted for age, age-squared, BMI, sex, smoking status (current versus other), and any hormone use (menopausal status and/or reproductive hormone use versus no hormone use). In the SMOFAM dataset, the NMR was log transformed and adjusted for age, aged-squared, BMI, sex, and smoking status. We constructed principal components of population genetic variation using 655 unlinked (*r* ^2^<0.5) DMET Plus Array SNPs in 323 PKTWIN individuals and 212 SMOFAM individuals, and used the first ten principal components to further adjust the NMR in each dataset. To avoid heterogeneity due to potential differential linkage disequilibrium among different continental population samples, genotype-phenotype analyses presented were performed on individuals who self-identified as White. Additive models were evaluated for each common SNP via Hierarchical Linear Modeling (HLM) [22] as our primary analysis (S1 File). We performed a sample-size weighted fixed-effect meta-analysis followed by *P*_ACT_ adjustment using test statistics from the HLM analyses. The meta-*P*_ACT_ procedure [23] assesses meta-analysis significance at two levels: adjusted for all SNPs tested within a gene, and within the entire experiment, accounting for intragenic SNP correlation.

### Stage II Participants

We used DNA samples and clinical data from self-identified White participants from eight clinical trials of smoking cessation therapies conducted in six US sites [12–18] to validate nominally significant DMET SNPs from Stage I with pretreatment NMR and prospective abstinence (Table 2). Treatment-seeking smokers (≥10 CPD, ≥18 years of age, recruited using local media and screened for eligibility), were randomized to placebo or active pharmacotherapy [12, 14–18], or were prescribed varenicline and randomized to three modes of behavioral therapy [13]; all participants were provided with multiple sessions of supportive therapy (individual, group, telephone, internet or combined modes). Trial participants provided a blood [12, 14–18] or a saliva [13] sample on study entry; blood was used for pretreatment NMR determination from two clinical trials [12] and blood or saliva for DNA extraction from eight trials [12–18].

**Table 2:** Clinical trial participant characteristics.

NCT Trial ID	00326781	00322205	00301145	00087880	00086385	01621009	01621022	00332644
Investigator	Lerman	Lerman	Swan	Hall	Hall	Baker	Baker	Baker
N genotyped	310	341	493	149	177	176	159	694
Age Mean (SD)	46.5 (10.9)	44.4 (11.6)	49.2 (11.4)	42.0 (9.6)	57.3 (5.9)	37.7 (11.3)	41.6 (11.1)	44.4 (11.7)
BMI Mean (SD)	27.6 (5.2)	26.9 (4.8)	27.8 (5.8)	26.4 (4.7)	26.5 (5.9)	26.6 (5.7)	26.6 (5.3)	28.8 (6.7)
College (%)	50.3	41.8	25.6	50.7	57.6	20.5	17.7	22.5
Female (%)	46.5	54.6	68.8	38.9	41.8	53.4	59.1	59.5
Married (%)	48.4	51.0	68.2	24.2	29.4	44.6	46.5	47.4
FTND Mean (SD)	5.56 (2.2)	5.34 (2.1)	5.17 (2.1)	4.80 (2.1)	4.86 (2.1)	5.10 (2.4)	5.67 (2.1)	5.23 (2.2)
CPD^a^ Mean (SD)	23.9 (9.5)	22.0 (9.4)	20.3 (8.3)	19.2 (7.6)	20.8 (8.8)	21.5 (8.3)	23.8 (9.4)	21.7 (8.9)
CPD^b^ Mean (SD)	1.57 (0.9)	1.36 (0.7)	1.21 (0.7)	1.15 (0.7)	1.26 (0.8)	1.54 (0.7)	1.68 (0.8)	1.47 (0.8)
TTFC Mean (SD)	2.11 (0.9)	2.09 (0.9)	2.09 (0.9)	1.78 (0.9)	1.93 (1.0)	1.78 (1.0)	2.15 (0.8)	1.92 (0.9)
Pharmacotherapy^c^	NRT	BUP, PLA	VAR	NRT+BUP	NRT+BUP	BUP, PLA	BUP, PLA	NR, BU, PL
N Arms	2	2	3	5	4	4	2	4
EOT ABS^d^ (%)	0.326	0.264	0.550	0.633	0.667	0.273	0.220	0.415
6MO^e^ ABS (%)	0.197	0.211	0.426	0.449	0.616	0.148	0.214	0.320

^a^Continuous.

^b^FTND/FTCD.

^c^Nicotine Replacement Therapy (NRT, NR), Bupropion (BUP, BU), Placebo (PLA, PL), Varenicline (VAR), combined NRT and BUP (NRT+BUP).

^d^EOT ABS = End of treatment seven day point prevalence abstinence, except for trials 00087880 and 00086385 which provided NRT+BUP for 12 weeks and then randomized participants to chronic NRT, chronic BUP or no further treatment.

^e^6MO = Six months.

### Stage II Analysis

For validation, we chose one or more SNPs with uncorrected meta-analysis *p*-values<0.05 from each of the ten genes with meta-P_ACT_ *p*-values<0.05 and evaluated availability of TaqMan SNP Genotyping Assays (Life Technologies). Of 16 selected SNPs, 12 SNPs had predesigned assays, two were proxy SNPs (*r* ^2^ = 1, Utah residents with ancestry from northern and western Europe HapMap sample, CEU) with predesigned assays, and two had custom assays designed (S1 File and S4 Table). We performed genotyping on the TaqMan OpenArray SNP Genotyping System or ViiA 7 using laboratory study, clinical trial, and HapMap (Coriell Cell Repositories, Camden, NJ) DNA samples. Stage II SNP genotyping assays were evaluated for clustering, concordance and completion rates prior to removing DNA samples with low completion rates, then for concordance, completion and Hardy Weinberg Equilibrium (HWE). Quality control procedures and summary results are described in S1 File, and completion rate and HWE significance by assay and by clinical trial are described in S5 and S6 Tables. Six of 16 selected DMET SNPs failed genotype clustering, completion rate and HWE quality thresholds. Thus, ten SNPs from seven of 10 genes passed quality thresholds and were included in subsequent analyses. In the course of evaluating the specificity of custom assays for one *CYP2A6* SNP (rs4803381), we resolved an ambiguous genomic location, i.e., evidence for rs4803381’s location proximal to *CYP2A6* rather than proximal to *CYP2A7* (S1 File).

We used linear regression to estimate association of ten DMET SNPs on natural log transformed NMR in treatment-seeking smokers from two trials [12], adjusted for age, age-squared, BMI, sex, the first three principal components of population genetic variation obtained from analysis of 45 ancestry informative markers [24], and site. We used logistic regression to estimate association between ten DMET SNPs on end of treatment and on six month abstinence in treatment-seeking smokers from eight trials [12–18], adjusting for age, age-squared, BMI, sex, education (college degree or less than a college degree), marital status (married or other), principal components, and 26 treatment arms. For both analyses, we imputed missing values for BMI (N = 43), education (N = 10) and marital status (N = 7) 20 times, analyses were performed on each data set, and results combined with adjustment to reflect the variance attributable to the imputations [25].

### *Post-hoc* analyses

For two *CYP2A6* SNPs (rs4803381 and rs1137115) we estimated the proportions of variance and significance of association with: cigarettes per day (CPD), coded as a continuous variable and as in the Fagerström Test for Nicotine Dependence (FTND) [26] or Cigarette Dependence (FTCD) [27]; time to first cigarette after waking (TTFC); and total FTND/FTCD score. Model covariates in nicotine dependence regressions were age, age squared, BMI, sex, education, marital status, three principal components of population genetic variation, and site. We performed regression analyses to evaluate the influence of rs4803381 on prospective abstinence outcomes stratified by pharmacotherapy. Model covariates in abstinence regressions were the same as in the nicotine dependence regressions with the addition of clinical trial arm. Power analyses of SNP associations with NMR and six month prospective abstinence were performed. The TRICL meta-GWAS database, comprised of multiple case:control studies of lung cancer [19], was interrogated for existing association results at two *CYP2A6* SNPs.

### Analysis software, annotation, and model parameters

We used: SAS (Cary, NC) for data curation and HWE testing; plink [28] to evaluate Mendelian transmission; GCTA [29] to estimate principal components; STATA (StataCorp, College Station, TX) to perform HLM, imputation and other regression analyses; *P*_ACT_ [30] and meta-*P*_ACT_ [23] to adjust for multiple testing; Haploview to calculate linkage disequlibrium [31]; SNAP [32] to identify proxy SNPs; Quanto [33] for power analyses; and dbSNP [34] and Affymetrix resources for SNP annotation. Chromosome coordinates are from the NCBI36/hg18 assembly [34]. SNP models were additive, tests were two-sided, and all alphas were 0.05.

## Results

### Stage I participants, common DMET SNPs and the NMR

PKTWIN participants are significantly (P < .001) more likely to be female, less likely to be a current smoker, and, lower in adiposity than SMOFAM participants (Table 1). Five SMOFAM individuals were excluded from analysis, one with a NMR value more than five standard deviates above the mean, and four due to genotypes inconsistent with interview-based family relationships. We excluded 121 and 28 DMET SNPs in PKTWIN and SMOFAM, respectively, from analysis due to nominally significant deviation (*p*-values<0.05) from HWE. We tested 608 DMET SNPs common in PKTWIN and 531 SNPs common in SMOFAM, and adjusted for multiple tests in genes with more than one SNP tested using the P_ACT_ procedure. We meta-analyzed 507 SNPs within 173 genes in 449 individuals from the PKTWIN and SMOFAM datasets and adjusted for multiple SNPs within a gene, and for two datasets, using the meta-*P*_ACT_ procedure. S1–S3 Tables contain genotype counts and HLM analyses results, meta-analysis Z scores and *p*-values, and meta-*P*_ACT_ gene-wise *p*-values, respectively. Ten genes exhibited gene-wise meta-P_ACT_ *p*-values<0.05; *CYP2A6*, *CYP2D6*, and *SPG7* were the top three ranked genes (Table 3). Forty-one SNPs had uncorrected meta-analysis *p*-values<0.05 with *CYP2A6* and *CYP2D6* SNPs in the top three ranked SNPs. rs4803381 was the top ranked SNP with an uncorrected meta-analysis *P* = 1.33E-07. After multiple test correction for 507 SNPs, two *CYP2A6* SNPs attained experiment-wide significance: rs4803381 (*P*_ACT_ = 4.53E-05) and rs1137115 (*P*_ACT_ = .0012), located in the promoter (c.-1013) and first exon (c.51) of *CYP2A6*. These SNPs are in strong linkage disequilibrium with each other (*r* ^2^ = 0.63 and 0.62 and D’ = 0.99 and 1.00 in PKFAM and SMOFAM), and in ex vivo hepatic tissue have been shown to be associated with significantly reduced protein and activity [35]. rs4803381 and rs1137115 are assocated with many CYP2A6* star allele (*) haplotypes (*1B/*1B2, *1B5, *1B6, *1B8, *1B9, *1B10, *1B11, *1D, *1J, *9A, *9B, *18C, *24A, *24B, *31A, *31B, *35A and *1A, *1B14, *1B17, *2, *14, *18B, *20, *21, *28A, *28B, *41, *42, *44, *45, respectively) [36].

**Table 3:** DMET genes associated with the laboratory study-based NMR, by meta-P_ACT_ *P*<0.05.

Gene	meta-*P*_*ACT*_	chr:coor of transcript^a^	SNPs ranked by meta-*P*_ACT_ *p*-value
*CYP2A6*	4.05E-07	chr19:46,041,283–46,048,192	rs4803381, rs1137115, rs4079369, rs8192729
*CYP2D6*	1.03E-03	chr22:40,852,445–40,856,827	rs1080985, rs28371725, rs16947, rs1080983, rs1065852
			rs28360521, rs1800716, rs3892097, rs1135840, rs1058164
*SPG7*	1.08E-02	chr16:88,102,306–88,151,675	rs12960, rs2292954
*XDH*	1.11E-02	chr2:31,410,692–31,491,115	rs1884725, rs2295475
*CHST8*	1.49E-02	chr19:38,804,701–38,956,254	rs1064349
*CHST13*	2.46E-02	chr3:127,725,866–127,744,824	rs1873397, rs6783962, rs4305381, rs1056522
*SLCO1B1*	2.46E-02	chr12:21,175,404–21,283,997	rs11045819, rs2291075, rs2306283, rs4149057, rs4149056
*CYP4F3*	2.66E-02	chr19:15,587,029–15,601,447	rs1805041, rs1805042
*SLC15A1*	4.42E-02	chr13:98,134,057–98,202,909	rs2297322, rs1339067
*CBR1*	4.76E-02	chr21:36,364,155–36,367,332	rs2835265, rs1005695, rs998383, rs3787728

^a^NCBI36/hg18

### Stage II participants, NMR and prospective abstinence

DNA samples from 2,499 clinical trial participants (Table 2) were subject to genotyping at 16 DMET SNPs, and to quality control filtering (S1 File and S4–S6 Tables). Two *CYP2A6* SNPs evaluated for association with pretreatment NMR were associated at genome-wide significance [*β* = -0.280, 95%CI (-0.336, -0.225), *P* = 1.25E-21, N = 633 individuals, and -0.240, 95%CI (-0.301, -0.178), *P* = 7.30E-14, N = 614 individuals]. The Stage II mean (standard deviation) completion rates for the two *CYP2A6* SNPs over the eight randomized clinical trials were: rs4803381, 0.9946 (0.0045) and rs1137115, 0.9834 (0.0218). One of eight clinical trial HWE *p*-values for rs4803381 was < .05, while all other *p*-values were >.155; no clinical trial HWE *p*-value for rs1137115 was < .05. The two *CYP2A6* SNPs are in strong linkage disequilibrium with each other (*r* ^2^ = 0.58 and D’ = 0.98 in the two clinical trials). No other analyzed DMET SNPs were statistically significantly associated with pretreatment NMR, including the *CYP2D6* SNP rs28371725 (Table 4). In joint analysis of rs4803381 and rs1137115 with baseline NMR (N = 605 individuals), rs4803381 was experiment-wide statistically significantly associated [*β* = -0.209, 95%CI (-0.293, -0.125), *P* = 1.31E-6], while rs1137115 was not [*β* = -0.075, 95%CI (-0.168, 0.017), *P* = 0.110]. Pretreatment NMR variance accounted for by covariates alone, with rs4803381, with rs1137115, and with both SNPs were 9.2%, 21.9%, 18.5%, and 22.2%. The mean (SD) and N of the residualized transformed NMR in individuals with SNP reference, heterozygote and minor allele homozygote genotypes were: -0.947 (0.153) 263, -0.950 (0.166) 296; and -0.953 (0.161) 74 for rs4803381; and -0.955 (0.156) 351, -0.951 (0.166) 228, -0.947 (0.143) 35 for rs1137115. Coefficient signs from Stage I and II analyses matched for both *CYP2A6* SNPs. No DMET SNPs were associated with prospective abstinence over all participants, at either time point (S7 Table); power to detect ORs of 1.2 to 1.4-fold was good to excellent (S8 Table).

**Table 4:** DMET SNP association with NMR, Stage I (PKTWIN, SMOFAM, Meta-analysis) and Stage II (RCT), by Stage I Meta-*P*_ACT_.

Gene	SNP^a^	*β*_PK_ ^b^	*SE*_PK_	*P*_PK_	*β*_SM_	*SE*_SM_	*P*_SM_	*Z*_Meta_	*P*_Meta_	*β*_RCT_	*SE*_RCT_	*P*_RCT_
*CYP2A6*	rs4803381	-0.054	0.014	0.000	-0.070	0.020	0.000	-5.274	1.3E-7	-0.280	0.028	1E-21
*CYP2A6*	rs1137115	-0.053	0.015	0.000	-0.068	0.023	0.003	-4.657	3.2E-6	-0.240	0.031	4E-14
*XDH*	rs1884725	0.040	0.014	0.004	0.014	0.024	0.569	2.768	0.0056	-0.002	0.033	0.949
*SLCO1B1*	rs11045819^c^	0.047	0.018	0.007	0.024	0.028	0.394	2.752	0.0059			
	rs17329885^d^									-0.012	0.040	0.767
*CYP4F3*	rs1805041	0.007	0.015	0.644	0.074	0.021	0.000	2.456	0.0140	0.039	0.031	0.216
*CBR1*	rs2835265^c^	0.056	0.022	0.012	0.022	0.033	0.497	2.447	0.0144			
	rs2835272^d^									0.068	0.045	0.133
*CYP2D6*	rs28371725	-0.048	0.019	0.011	-0.005	0.035	0.888	-2.299	0.0215	0.013	0.047	0.779
*SLC15A1*	rs2297322	-0.022	0.020	0.275	-0.079	0.031	0.012	-2.284	0.0224	0.031	0.044	0.481
*CYP4F3*	rs1805042	-0.015	0.012	0.229	-0.041	0.018	0.023	-2.272	0.0231	-0.020	0.031	0.520
*SLCO1B1*	rs2306283	0.013	0.013	0.319	0.049	0.020	0.013	2.178	0.0294	0.018	0.030	0.558

^a^See S4 Table for details on SNPs.

^b^ *β*, *P* = coefficient and *P* from PKTWIN [9] (PK), SMOFAM [10] (SM), and two clinical trials [12] (RCT), respectively; *SE* = Standard Error; *Z*_Meta_ = meta-analysis Z score; and *P*_Meta_ = meta-analysis *P*_ACT_.

^c,d^Proxy SNPs, *r* ^2^ = 1.0, CEU for laboratory^c^ and clinical trial^d^ analyses, respectively.

### *Post-hoc* analyses

rs4803381 and rs1137115 were statistically significantly associated with cigarettes per day at different levels of significance (continuous, *p*-values of .0001 and .0017; categorical, *p*-values of .0020 and .0215), but not with TTFC or with total FTND (Table 5 and S9 Table). In joint analysis of rs4803381 and rs1137115, rs4803381 remained nominally associated with CPD (continuous, *P* = .0279; categorical, *P* = .0376).

**Table 5:** *Post-hoc* analyses of *CYP2A6* SNPs and measures of nicotine dependence^a^ in treatment-seeking smokers.

	CPD (continuous)	CPD (FTND coding)	TTFC	FTND
Mean (SD)	21.67 (8.95)	1.34 (0.77)	1.99 (0.90)	5.23 (2.18)
Min, Max	1, 100	0, 3	0, 3	0, 10
Covariates^b^ *r* ^2^	0.1064	0.1056	0.0633	0.0674
Covariates and rs4803381 *r* ^2^	0.1118	0.1092	0.0633	0.0678
rs4803381 *p*-values	0.0001	0.0020	0.9045	0.2938
Covariates and rs1137115, *r* ^2^	0.1101	0.1076	0.0633	0.0678
rs1137115, *p*-values	0.0017	0.0215	0.8415	0.2938
Covariates and both *CYP2A6* SNPs, *r* ^2^	0.1119	0.1092	0.0633	0.0678
rs4803381 and rs1137115, *p*-values	0.0279 0.6966	0.0376, 0.9522	0.9601, 0.8650	0.6966, 0.6892

^a^Pairwise correlations of cigarettes per day (continuous) with categorical CPD, TTFC and FTND were 0.914, 0.400 and 0.614, of categorical CPD with TTFC and FTND were 0.379 and 0.622, and of TTFC with FTND were 0.799.

^b^Age, age squared, BMI, sex, education, marital status, three principal components of population genetic variation and site. Descriptive and regression analyses were performed on a sample of 2,418 individuals with called genotypes at both rs4803381 and rs1137115.

In analyses of end-of-treatment and six month abstinence among individuals randomized to one of six (end-of-treatment) and to one of nine (six months) different pharmacotherapies, we observed two results of interest (S10 Table). In the first result of interest, we observed increased abstinence in individuals randomized to nicotine replacement therapy (NRT) (S10 Table). After analyses by mode of administration, we observed increased abstinence at end-of-treatment and at six months, with a trend towards significance (*p*-values < .10) in individuals randomized to NRT patch [in 339 individuals, OR_end-of-treatment_ (95%CI) *P* = 1.348 (0.961–1.890) 0.084 and OR_six months_ (95%CI) *P* = 1.386 (0.973–1.975) 0.071]. These observations, while not statistically significant, are the most directly interpretable. We also observed a nominally statistically significant rs4803381 association with six month abstinence in one arm of 66 individuals first treated with combined NRT and bupropion from baseline to 12 weeks and then randomized to chronic bupropion (*P* = 0.023). This result has a more speculative interpretation.

rs1137115 was nominally significantly associated with lung cancer in a meta-analysis of four studies from the TRICL database [fixed effects odds ratio (95%CI) *P* 0.92 (0.87–0.97) 0.0022], and in two of the individual case:control studies (S11 Table). rs4803381 does not have results in the TRICL database. The direction of effect of the rs1137115 association with lung cancer is consistent with the reduced nicotine metabolism and cigarette consumption associated with the rs1137115 minor allele we observed in our Stage I and II analyses, and with the functional effects of rs1137115 evaluated in *ex vivo* hepatic tissue [35, 37].

## Discussion

### Findings

There were five novel findings in this study, two in the *a priori* phase, and three in the *post-hoc* phase. *CYP2A6* was associated with laboratory based NMR at experiment-wide significance in an analysis of 173 DMET genes. rs4803381 and rs1137115 were associated with the pretreatment NMR at genome-wide significance. In *post-hoc* analyses in clinical trial participants, these two *CYP2A6* SNPs were shown to be associated at experiment-wide significance with baseline CPD in treatment-seeking smokers of eight clinical trials, and rs4803381 was associated with six month abstinence in individuals randomized to chronic bupropion treatment at nominal significance. In the *post-hoc* meta-analysis of four case:control studies, rs1137115 was associated with lung cancer at nominal significance. The minor alleles of these *CYP2A6* SNPs were associated with reduced nicotine metabolism, smoking heaviness and lung cancer risk in a mechanistically interpretable fashion (reduced nicotine metabolism rate reduces cigarette consumption reduces lung cancer risk).

### rs4803381, rs1137115, and correlates

Our multivariate analysis of two *CYP2A6* SNPs, demographics, population genetic variation and clinical trial site accounted for up to 22.2% of the variance of the pretreatment NMR with the individual SNPs accounting for approximately half of this variance. Validation of the association of *CYP2A6* SNPs with the NMR confirms the role CYP2A6 plays in nicotine metabolism [7, 38, 39]. Both SNPs are common polymorphisms in continental ancestry samples, where the rs4803381 European ancestry minor allele is the major allele in Asian and African HapMap populations. The minor alleles of rs4803381 and rs1137115 are associated with reduced *CYP2A6* transcript levels [35, 37], CYP2A6 protein level [35], and CYP2A6 activity [35, 37] and are linked with reduced function *CYP2A6* alleles [36] (S4 Table).

rs4803381 and rs1137155 were significantly associated with cigarette consumption coded continuously or as in the FTND/FTCD (Table 5). The influence of a single allele of rs4803381 and of rs1137115 in our multivariate regressions of treatment-seeking smokers were -0.11 and -0.098 standard deviates of continuously coded CPD, or a reduction of 0.99 and 0.88 cigarette per day (unadjusted means in S9 Table), which represent effect sizes larger than those associated with other *CYP2A6* SNPs, e.g., rs1801272 (c.479T>A/p.L160H), with effect size of 0.68 CPD (N = 66,380, *P* = 1.1E-4) [40]. rs1137115 was nominally significantly associated with lung cancer in a meta-GWAS database, adding to existing evidence that other *CYP2A6* variants contribute to lung cancer risk (S2 File). The functional effects of rs4803381 and rs1137115, i.e., reduced transcription, protein level and activity [35, 37] suggest that *CYP2A6* variation that influences regulation may be more important than *CYP2A6* non-synonymous variants that directly influence enzyme activity, e.g., rs1801272, but with lower prevalence [41].

The validated *CYP2A6* SNPs were not associated with the clinically relevant outcome of abstinence in all trial participants, adjusted for pharmacotherapy. However, when we performed analyses stratified by pharmacotherapy, we observed interesting effect sizes in several smaller strata, including trending associations with increased abstinence in individuals randomized to NRT patch, and nonminally significant decreased abstinence in individuals randomized to chronic bupropion. The former result is consistent with reported results in individuals from two trials [18, 42] analyzed together here, with the influence of *CYP2A6* activity on abstinence previously evaluated using the biochemical NMR [42], or the CYP2A6 activity model [43]. In our analysis of indivdiuals randomized to NRT patch from two trials [18, 42], we find that a single *CYP2A6* SNP is a statistically trending predictor where reduced nicotine metabolism implies increased nicotine plasma levels and increased abstinence. The latter result in individuals randomized to chronic bupropion therapy reaches our *a priori* nominally statistically significant threshold but is of uncertain clinical significance due to the small size of the pharmacotherapy group and multiple pharmacotherapy groups examined. A speculative interpretation might suggest that linkage disequilibrium between rs4803381 and *CYP2B6* SNPs that influence expression or activity might influence six month abstinence in indivduals undergoing chronic treatment with bupropion. Interaction between *CYP2A6* and *CYP2B6* variants in association with nicotine metabolism has been reported [44]. *CYP2B6* SNPs that influence bupropion metabolism also influence abstinence in individuals at six months in individuals treated with bupropion for 10 weeks [45], and linkage disequilbrium between relevant *CYP2A6* and *CYP2B6* alleles is low [46]. *CYP2B6* association with smoking heaviness has been reported at robust statistical significance (rs7260329, *P* = 6E-6) in a genome-wide scan [40]. Mechanistic understanding of the potential association of rs4803381 and six month abstinence in individuals randomized to chronic bupropion treatment awaits replication and more data to permit testing of *CYP2A6* and *CYP2B6* metabolic and treatment outcome hypotheses.

### Limitations

In this study, we did not identify the monooxygenase gene *FMO3* in our Stage I meta-analysis; *FMO3* haplotypes were previously associated with the ratio of cotinine to the sum of nicotine and cotinine in a laboratory study [47], with CPD in dependent smokers [47], and with the NMR among slow metabolizers [48]. Future work should include haplotype and diplotype analyses to evaluate the joint influence of multiple variants. Limitations of DMET Plus array gene coverage contributed to the inability to identify some DMET gene variants previously associated with nicotine metabolism. Finally, cytochrome P450 SNPs were over-represented among DMET SNPs that failed quality control thresholds in Stage II of our analysis, suggesting that methods that more effectively interrogate cytochrome P450 loci variation may enable further analyses of NMR variance and related phenotypes at these loci. Significant *CYP2A6* SNP associations with CPD, with prospective abstinence by pharmacotherapy, and with lung cancer were *post-hoc* and were not replicated in this study. In addition, findings from analyses of treatment-seeking cigarette smokers may not generalize to all tobacco product smokers.

## Conclusions

In this two-stage scan of DMET gene variation association with the NMR, we utilized two of the largest laboratory-based studies of nicotine metabolism to nominate DMET genes and SNPs, and eight randomized controlled trials of smoking cessation therapies and a lung cancer meta-GWAS database, to validate novel SNP associations with the NMR and to explore associations with related phenotypes. We ranked DMET candidate genes and SNPs by their association with the laboratory study NMR using a single model. We validated association of *CYP2A6* SNPs with a similar phenotype, the clinical trial-based pretreatment NMR. This suggests that multiple *CYP2A6* polymorphisms and non-genetic covariates will improve the predictive power of CYP2A6 activity models and support a continued focus on CYP2A6 activity models for use where phenotypic measures of nicotine metabolites are not available.

Enhanced knowledge of the genes that influence nicotine metabolism, smoking behaviors and clinical outcomes will help to characterize the risks from gene variants on smoking behaviors and smoking-attributable disease, identify novel biomarkers for therapeutic efficacy, and novel targets for the development of smoking cessation therapies. Based on this analysis and additional studies cited, *CYP2A6* SNPs account for large fractions of the variance of the NMR, smaller fractions of the variance of cigarette consumption, and influence risk for lung cancer, but do not account for other nicotine dependence factors. This study does not provide guidance on the use of individual DMET SNPs to assign clinical treatment for tobacco dependence as the findings of interest in this study were unreplicated. Research must continue to improve our understanding of factors influencing tobacco product exposures and attributable disease, to develop improved interventions, and to protect the public health.

## Supporting Information

S1 Table PKTWIN and SMOFAM DMET Plus SNP HLM Results. (DOCX)

S2 Table PKFAM DMET Plus SNP Meta-analysis Results. (DOCX)

S3 Table Meta-P_ACT_ Gene-wise Results. (DOCX)

S4 Table SNPs chosen for TaqMan SNP Genotyping Assay Genotyping. (DOCX)

S5 Table TaqMan SNP Genotyping Assay completion rate, by RCT. (DOCX)

S6 Table TaqMan SNP Genotyping Assay HWE Exact *p*-value, by RCT. (DOCX)

S7 Table DMET SNPs and Prospective Abstinence in Eight RCTs. (DOCX)

S8 Table Power to Detect SNP Odds Ratios, Six Month Abstinence. (DOCX)

S9 Table rs4803381, rs1137115 and Measures of Nicotine Dependence. (DOCX)

S10 Table rs4803381 and Abstinence by Pharmacotherapy Randomization. (DOCX)

S11 Table TRICL rs1137115 Lung Cancer Results. (DOCX)

S1 File Discovery (Stage I) and Validation (Stage II) Analyses. (DOCX)

S2 File Prior CYP2A6 Associations with NMR, Abstinence, CPD and Lung Cancer Risk. (DOCX)
