# Supplementary material for: Drug Metabolizing Enzyme and Transporter Gene Variation, Nicotine Metabolism, Prospective Abstinence, and Cigarette Consumption
Source: PLoS One. 2015 Jul 1;10(7):e0126113. doi: 10.1371/journal.pone.0126113 (PMC4488893; doi:10.1371/journal.pone.0126113)
Supplement: S1 Table — (DOCX) [file pone.0126113.s001.docx]

**S1 Table. PKTWIN and SMOFAM DMET^™^ Plus SNP HLM Results**^a^

|  |  |  |  | **PKTWIN** | | | | | | | **SMOFAM** | | | | | | |
| --- | --- | --- | --- | --- | --- | --- | --- | --- | --- | --- | --- | --- | --- | --- | --- | --- | --- |
| **rsID** | **Gene** | **Chr:coor** | **Probe** | **C_11** | **C_12** | **C_22** | **MAF** | **coef** | **se** | **P** | **C_11** | **C_12** | **C_22** | **MAF** | **coef** | **se** | **P** |
| rs592792 | *GSTM4* | 1:110013479 | am_11695 | 198 | 55 | 2 | 0.116 | -0.003 | 0.022 | 0.898 | 146 | 56 | 1 | 0.143 | -0.053 | 0.027 | 0.050 |
| rs1296954 | *GSTM5* | 1:110055934 | am_11710 | 69 | 152 | 33 | 0.429 | 0.002 | 0.015 | 0.887 | 71 | 105 | 27 | 0.392 | 0.025 | 0.022 | 0.261 |
| rs2479390 | *GSTM5* | 1:110057598 | am_11719 | 163 | 82 | 10 | 0.200 | -0.001 | 0.016 | 0.957 | 127 | 62 | 14 | 0.222 | 0.044 | 0.024 | 0.059 |
| rs11807 | *GSTM5* | 1:110062265 | am_11724 | 163 | 88 | 4 | 0.188 | 0.008 | 0.017 | 0.655 | 112 | 77 | 14 | 0.259 | 0.004 | 0.025 | 0.867 |
| rs7483 | *GSTM3* | 1:110081224 | am_11730 | 137 | 87 | 31 | 0.292 | -0.013 | 0.013 | 0.321 | 98 | 87 | 18 | 0.303 | -0.012 | 0.020 | 0.526 |
| rs1799735 | *GSTM3* | 1:110081777 | am_11734 | 174 | 70 | 11 | 0.180 | 0.005 | 0.016 | 0.755 | 144 | 52 | 7 | 0.163 | -0.007 | 0.026 | 0.777 |
| rs12727968 | *SLC16A1* | 1:113253807 | am_11739 | 181 | 65 | 9 | 0.163 | -0.007 | 0.018 | 0.704 | 166 | 34 | 2 | 0.094 | -0.043 | 0.036 | 0.232 |
| rs9429505 | *SLC16A1* | 1:113256094 | am_11740 | 156 | 90 | 7 | 0.206 | 0.014 | 0.016 | 0.385 | 125 | 70 | 8 | 0.212 | 0.020 | 0.026 | 0.436 |
| rs7169 | *SLC16A1* | 1:113256622 | am_11742 | 73 | 134 | 48 | 0.451 | 0.001 | 0.014 | 0.941 | 68 | 104 | 31 | 0.409 | -0.023 | 0.020 | 0.240 |
| rs1049434 | *SLC16A1* | 1:113258069 | am_11744 | 73 | 134 | 48 | 0.451 | 0.001 | 0.014 | 0.941 | 68 | 104 | 31 | 0.409 | -0.023 | 0.020 | 0.240 |
| rs2228099 | *ARNT* | 1:149075513 | am_11804 | 89 | 124 | 41 | 0.406 | -0.010 | 0.013 | 0.445 | 84 | 104 | 15 | 0.330 | 0.058 | 0.019 | 0.003 |
| rs5085 | *NR1I3* | 1:159459135 | am_11809 | 161 | 84 | 10 | 0.204 | -0.010 | 0.019 | 0.589 | 132 | 65 | 5 | 0.186 | 0.022 | 0.026 | 0.397 |
| rs11584174 | *NR1I3* | 1:159479077 | am_11833 | 174 | 70 | 11 | 0.180 | -0.007 | 0.016 | 0.684 | 148 | 53 | 2 | 0.140 | -0.054 | 0.030 | 0.072 |
| rs2501870 | *NR1I3* | 1:159479193 | am_11834 | 138 | 103 | 14 | 0.257 | 0.008 | 0.015 | 0.615 | 103 | 80 | 19 | 0.292 | -0.013 | 0.022 | 0.557 |
| rs55802895 | *NR1I3* | 1:159479367 | am_11835 | 173 | 72 | 9 | 0.177 | -0.002 | 0.017 | 0.884 | 148 | 53 | 2 | 0.140 | -0.054 | 0.030 | 0.072 |
| rs2266782 | *FMO3* | 1:169343590 | am_11875 | 87 | 142 | 26 | 0.380 | 0.007 | 0.014 | 0.639 | 54 | 118 | 31 | 0.443 | -0.040 | 0.020 | 0.049 |
| rs2066534 | *FMO3* | 1:169343996 | am_11880 | 144 | 104 | 7 | 0.231 | -0.004 | 0.017 | 0.800 | 108 | 86 | 9 | 0.256 | -0.017 | 0.026 | 0.519 |
| rs909530 | *FMO3* | 1:169349798 | am_11888 | 171 | 68 | 16 | 0.196 | 0.007 | 0.014 | 0.627 | 126 | 56 | 21 | 0.241 | -0.008 | 0.024 | 0.729 |
| rs2266780 | *FMO3* | 1:169349866 | am_11891 | 187 | 62 | 6 | 0.145 | 0.013 | 0.017 | 0.445 | 140 | 52 | 11 | 0.182 | -0.040 | 0.026 | 0.128 |
| rs1736565 | *FMO6* | 1:169379114 | am_11912 | 54 | 145 | 56 | 0.504 | -0.005 | 0.013 | 0.691 | 59 | 110 | 34 | 0.438 | 0.005 | 0.020 | 0.814 |
| rs2272797 | *FMO6* | 1:169385464 | am_11913 | 193 | 56 | 5 | 0.130 | 0.017 | 0.018 | 0.335 | 146 | 46 | 8 | 0.155 | -0.029 | 0.031 | 0.338 |
| rs7889839 | *FMO6* | 1:169387797 | am_11914 | 193 | 57 | 5 | 0.131 | 0.017 | 0.018 | 0.335 | 147 | 48 | 8 | 0.158 | -0.030 | 0.030 | 0.321 |
| rs7886938 | *FMO6* | 1:169387892 | am_11915 | 193 | 57 | 5 | 0.131 | 0.017 | 0.018 | 0.335 | 147 | 48 | 8 | 0.158 | -0.030 | 0.030 | 0.321 |
| rs2020861 | *FMO2* | 1:169435209 | am_11938 | 67 | 147 | 41 | 0.449 | -0.008 | 0.015 | 0.562 | 62 | 106 | 35 | 0.433 | -0.030 | 0.022 | 0.174 |
| rs2020863 | *FMO2* | 1:169441155 | am_11942 | 202 | 51 | 2 | 0.108 | -0.032 | 0.022 | 0.132 | 175 | 25 | 2 | 0.072 | -0.002 | 0.044 | 0.956 |
| rs7512785 | *FMO2* | 1:169445911 | am_11968 | 136 | 104 | 15 | 0.263 | 0.000 | 0.017 | 0.997 | 98 | 90 | 15 | 0.296 | -0.065 | 0.026 | 0.013 |
| rs7515157 | *FMO2* | 1:169446101 | am_11970 | 136 | 104 | 15 | 0.263 | 0.000 | 0.017 | 0.997 | 98 | 90 | 15 | 0.296 | -0.065 | 0.026 | 0.013 |
| rs742350 | *FMO1* | 1:169516668 | am_12017 | 182 | 69 | 4 | 0.151 | -0.009 | 0.019 | 0.613 | 139 | 61 | 3 | 0.165 | 0.008 | 0.028 | 0.773 |
| rs1126692 | *FMO1* | 1:169518911 | am_12023 | 182 | 69 | 4 | 0.151 | -0.009 | 0.019 | 0.613 | 139 | 61 | 3 | 0.165 | 0.008 | 0.028 | 0.773 |
| rs12954 | *FMO1* | 1:169521418 | am_12028 | 195 | 52 | 8 | 0.133 | -0.013 | 0.017 | 0.448 | 149 | 50 | 4 | 0.143 | -0.013 | 0.029 | 0.665 |
| rs7877 | *FMO1* | 1:169521514 | am_12031 | 131 | 101 | 22 | 0.285 | -0.013 | 0.014 | 0.354 | 95 | 91 | 17 | 0.308 | -0.003 | 0.023 | 0.910 |
| rs7541966 | *FMO4* | 1:169573779 | am_12057 | 176 | 66 | 4 | 0.150 | 0.009 | 0.019 | 0.630 | 124 | 55 | 5 | 0.177 | 0.001 | 0.029 | 0.982 |
| rs2223477 | *FMO4* | 1:169575456 | am_12058 | 127 | 95 | 32 | 0.313 | 0.009 | 0.013 | 0.467 | 89 | 92 | 22 | 0.335 | -0.007 | 0.022 | 0.762 |
| rs2072671 | *CDA* | 1:20788288 | am_11499 | 122 | 115 | 18 | 0.296 | -0.015 | 0.014 | 0.289 | 105 | 80 | 18 | 0.286 | -0.022 | 0.022 | 0.302 |
| rs818202 | *CDA* | 1:20789378 | am_11500 | 90 | 122 | 43 | 0.408 | 0.014 | 0.013 | 0.299 | 69 | 99 | 35 | 0.416 | 0.032 | 0.020 | 0.115 |
| rs1048977 | *CDA* | 1:20817642 | am_11520 | 114 | 113 | 28 | 0.331 | -0.010 | 0.014 | 0.474 | 101 | 81 | 21 | 0.303 | 0.003 | 0.022 | 0.883 |
| rs1051740 | *EPHX1* | 1:224086256 | am_12092 | 138 | 96 | 21 | 0.271 | 0.015 | 0.014 | 0.277 | 96 | 97 | 10 | 0.288 | -0.037 | 0.023 | 0.104 |
| rs2292566 | *EPHX1* | 1:224086276 | am_12093 | 175 | 78 | 2 | 0.161 | -0.006 | 0.019 | 0.752 | 157 | 45 | 1 | 0.116 | -0.001 | 0.031 | 0.977 |
| rs324420 | *FAAH* | 1:46643348 | am_11525 | 156 | 89 | 9 | 0.211 | 0.025 | 0.016 | 0.115 | 115 | 78 | 9 | 0.238 | 0.003 | 0.022 | 0.876 |
| rs3215983 | *CYP4B1* | 1:47053334 | am_11546 | 169 | 84 | 2 | 0.173 | 0.011 | 0.019 | 0.556 | 132 | 66 | 5 | 0.187 | -0.028 | 0.025 | 0.258 |
| rs2297810 | *CYP4B1* | 1:47053446 | am_11549 | 162 | 91 | 2 | 0.186 | 0.005 | 0.018 | 0.794 | 132 | 66 | 5 | 0.187 | -0.028 | 0.025 | 0.258 |
| rs4646491 | *CYP4B1* | 1:47053471 | am_11550 | 169 | 84 | 2 | 0.173 | 0.011 | 0.019 | 0.556 | 132 | 66 | 5 | 0.187 | -0.028 | 0.025 | 0.258 |
| rs2297809 | *CYP4B1* | 1:47055359 | am_11554 | 169 | 82 | 4 | 0.176 | 0.001 | 0.018 | 0.937 | 132 | 66 | 5 | 0.187 | -0.028 | 0.025 | 0.258 |
| rs11211402 | *CYP4A11* | 1:47164641 | am_11564 | 190 | 63 | 2 | 0.131 | 0.022 | 0.019 | 0.249 | 156 | 44 | 3 | 0.123 | -0.035 | 0.028 | 0.208 |
| rs4926802 | *CYP4Z1* | 1:47344489 | am_11609 | 110 | 121 | 12 | 0.298 | -0.009 | 0.016 | 0.564 | 107 | 76 | 14 | 0.264 | -0.033 | 0.022 | 0.138 |
| rs7512729 | *CYP4Z1* | 1:47351058 | am_11611 | 91 | 131 | 33 | 0.386 | 0.007 | 0.014 | 0.614 | 89 | 83 | 31 | 0.357 | -0.032 | 0.019 | 0.096 |
| rs890293 | *CYP2J2* | 1:60165082 | am_11637 | 229 | 25 | 1 | 0.053 | -0.039 | 0.030 | 0.188 | 175 | 25 | 3 | 0.076 | 0.047 | 0.034 | 0.160 |
| rs2297595 | *DPYD* | 1:97937679 | am_11667 | 201 | 50 | 2 | 0.107 | 0.013 | 0.022 | 0.557 | 165 | 33 | 5 | 0.106 | 0.035 | 0.029 | 0.230 |
| rs1801265 | *DPYD* | 1:98121473 | am_11669 | 159 | 77 | 19 | 0.225 | 0.007 | 0.014 | 0.643 | 128 | 62 | 13 | 0.217 | 0.004 | 0.023 | 0.867 |
| rs1530031 | *CHST10* | 2:100375758 | am_12523 | 91 | 103 | 58 | 0.435 | -0.008 | 0.012 | 0.478 | 61 | 105 | 37 | 0.441 | -0.028 | 0.020 | 0.150 |
| rs1530030 | *CHST10* | 2:100376032 | am_12526 | 87 | 117 | 51 | 0.429 | 0.002 | 0.013 | 0.898 | 54 | 114 | 35 | 0.453 | -0.036 | 0.020 | 0.076 |
| rs3748930 | *CHST10* | 2:100376514 | am_12532 | 79 | 117 | 58 | 0.459 | -0.004 | 0.012 | 0.767 | 52 | 114 | 37 | 0.463 | -0.038 | 0.020 | 0.055 |
| rs3828193 | *CHST10* | 2:100397993 | am_12535 | 87 | 117 | 51 | 0.429 | 0.002 | 0.013 | 0.898 | 56 | 113 | 34 | 0.446 | -0.036 | 0.020 | 0.069 |
| rs1402467 | *SULT1C2* | 2:108361240 | am_12590 | 166 | 77 | 11 | 0.195 | -0.042 | 0.016 | 0.007 | 132 | 60 | 11 | 0.202 | 0.015 | 0.024 | 0.533 |
| rs1050891 | *HNMT* | 2:138488230 | am_12643 | 158 | 85 | 12 | 0.214 | -0.012 | 0.015 | 0.430 | 140 | 56 | 7 | 0.172 | -0.007 | 0.029 | 0.798 |
| rs4245861 | *HNMT* | 2:138489164 | am_12646 | 158 | 85 | 12 | 0.214 | -0.012 | 0.015 | 0.430 | 140 | 56 | 7 | 0.172 | -0.007 | 0.029 | 0.798 |
| rs4646333 | *HNMT* | 2:138489699 | am_12647 | 158 | 85 | 12 | 0.214 | -0.012 | 0.015 | 0.430 | 140 | 56 | 7 | 0.172 | -0.007 | 0.029 | 0.798 |
| rs496550 | *ABCB11* | 2:169487958 | am_12655 | 82 | 132 | 41 | 0.420 | 0.023 | 0.013 | 0.084 | 54 | 107 | 42 | 0.470 | -0.039 | 0.020 | 0.053 |
| rs495714 | *ABCB11* | 2:169488010 | am_12656 | 82 | 131 | 42 | 0.422 | 0.021 | 0.013 | 0.114 | 52 | 109 | 42 | 0.475 | -0.038 | 0.020 | 0.059 |
| rs473351 | *ABCB11* | 2:169488142 | am_12658 | 90 | 128 | 37 | 0.396 | 0.020 | 0.013 | 0.129 | 56 | 110 | 36 | 0.450 | -0.041 | 0.020 | 0.042 |
| rs497692 | *ABCB11* | 2:169497262 | am_12674 | 83 | 129 | 42 | 0.419 | 0.015 | 0.013 | 0.258 | 52 | 109 | 42 | 0.475 | -0.038 | 0.020 | 0.059 |
| rs2287622 | *ABCB11* | 2:169538574 | am_12726 | 93 | 121 | 41 | 0.398 | -0.008 | 0.013 | 0.539 | 96 | 87 | 20 | 0.313 | -0.020 | 0.023 | 0.393 |
| rs7602171 | *ABCB11* | 2:169588424 | am_12778 | 118 | 109 | 28 | 0.324 | 0.001 | 0.014 | 0.914 | 120 | 65 | 18 | 0.249 | -0.011 | 0.026 | 0.674 |
| rs3770602 | *ABCB11* | 2:169589937 | am_12780 | 118 | 107 | 30 | 0.327 | 0.001 | 0.014 | 0.966 | 120 | 65 | 18 | 0.249 | -0.011 | 0.026 | 0.674 |
| rs4668115 | *ABCB11* | 2:169592894 | am_12783 | 119 | 113 | 23 | 0.312 | -0.001 | 0.014 | 0.962 | 127 | 64 | 12 | 0.217 | -0.023 | 0.026 | 0.389 |
| rs3731722 | *AOX1* | 2:201242634 | am_12843 | 230 | 21 | 4 | 0.057 | -0.036 | 0.026 | 0.160 | 175 | 24 | 4 | 0.079 | 0.006 | 0.035 | 0.858 |
| rs7563682 | *AOX1* | 2:201247451 | am_12847 | 107 | 111 | 37 | 0.363 | 0.004 | 0.013 | 0.771 | 79 | 88 | 36 | 0.394 | 0.011 | 0.019 | 0.547 |
| rs11684227 | *AOX1* | 2:201248778 | am_12848 | 94 | 124 | 37 | 0.388 | 0.002 | 0.013 | 0.854 | 71 | 91 | 41 | 0.426 | 0.018 | 0.018 | 0.337 |
| rs11678615 | *AOX1* | 2:201249866 | am_12849 | 230 | 21 | 4 | 0.057 | -0.036 | 0.026 | 0.160 | 175 | 24 | 4 | 0.079 | 0.018 | 0.034 | 0.587 |
| rs6729738 | *AOX1* | 2:201251741 | am_12852 | 52 | 139 | 64 | 0.524 | 0.003 | 0.013 | 0.813 | 52 | 95 | 56 | 0.510 | -0.026 | 0.018 | 0.163 |
| rs1048013 | *CYP20A1* | 2:203862797 | am_12857 | 92 | 127 | 36 | 0.390 | 0.009 | 0.014 | 0.533 | 70 | 91 | 42 | 0.431 | -0.005 | 0.018 | 0.765 |
| rs3832043 | *UGT1A9* | 2:234245202 | am_12947 | 86 | 120 | 49 | 0.427 | 0.002 | 0.014 | 0.867 | 84 | 91 | 28 | 0.362 | 0.028 | 0.021 | 0.175 |
| rs7586110 | *UGT1A1* | 2:234255266 | am_12956 | 110 | 106 | 39 | 0.361 | -0.007 | 0.016 | 0.659 | 68 | 98 | 37 | 0.424 | -0.001 | 0.028 | 0.962 |
| rs6759892 | *UGT1A9* | 2:234266408 | am_12969 | 97 | 117 | 41 | 0.390 | -0.015 | 0.015 | 0.344 | 66 | 94 | 43 | 0.443 | -0.010 | 0.030 | 0.733 |
| rs1105880 | *UGT1A9* | 2:234266704 | am_12973 | 111 | 118 | 26 | 0.333 | -0.027 | 0.018 | 0.127 | 77 | 97 | 29 | 0.382 | 0.019 | 0.032 | 0.550 |
| rs2070959 | *UGT1A6* | 2:234266930 | am_12974 | 124 | 106 | 24 | 0.303 | -0.019 | 0.018 | 0.303 | 80 | 94 | 25 | 0.362 | 0.030 | 0.031 | 0.329 |
| rs3755320 | *UGT1A6* | 2:234286800 | am_12982 | 209 | 44 | 2 | 0.094 | 0.034 | 0.023 | 0.134 | 173 | 29 | 1 | 0.076 | -0.028 | 0.033 | 0.397 |
| rs3821242 | *UGT1A9* | 2:234302542 | am_13005 | 90 | 116 | 47 | 0.415 | 0.010 | 0.015 | 0.493 | 59 | 94 | 48 | 0.473 | -0.009 | 0.024 | 0.705 |
| rs6706232 | *UGT1A3* | 2:234302592 | am_13007 | 90 | 114 | 44 | 0.407 | 0.010 | 0.015 | 0.499 | 60 | 94 | 48 | 0.470 | -0.009 | 0.024 | 0.708 |
| rs7574296 | *UGT1A9* | 2:234302988 | am_13011 | 69 | 117 | 48 | 0.455 | 0.006 | 0.015 | 0.707 | 44 | 95 | 48 | 0.511 | 0.002 | 0.025 | 0.950 |
| rs4124874 | *UGT1A1* | 2:234330398 | am_13018 | 88 | 115 | 50 | 0.425 | 0.007 | 0.014 | 0.601 | 59 | 96 | 48 | 0.473 | -0.008 | 0.023 | 0.747 |
| rs10929302 | *UGT1A1* | 2:234330521 | am_13019 | 135 | 99 | 21 | 0.276 | -0.007 | 0.017 | 0.659 | 82 | 99 | 20 | 0.346 | 0.021 | 0.027 | 0.449 |
| rs1976391 | *UGT1A1* | 2:234330722 | am_13020 | 127 | 105 | 23 | 0.296 | -0.016 | 0.017 | 0.339 | 82 | 97 | 24 | 0.357 | 0.023 | 0.028 | 0.402 |
| rs3755319 | *UGT1A1* | 2:234332321 | am_13021 | 90 | 115 | 50 | 0.422 | 0.008 | 0.014 | 0.559 | 60 | 95 | 48 | 0.470 | -0.009 | 0.024 | 0.710 |
| rs887829 | *UGT1A1* | 2:234333309 | am_13022 | 127 | 105 | 23 | 0.296 | -0.016 | 0.017 | 0.339 | 82 | 97 | 24 | 0.357 | 0.023 | 0.028 | 0.402 |
| rs34815109 | *UGT1A1* | 2:234333620 | am_13024 | 128 | 98 | 21 | 0.283 | -0.015 | 0.017 | 0.370 | 82 | 92 | 24 | 0.354 | 0.024 | 0.028 | 0.403 |
| rs10929303 | *UGT1A1* | 2:234346155 | am_13067 | 147 | 96 | 12 | 0.235 | -0.042 | 0.016 | 0.008 | 126 | 69 | 8 | 0.209 | 0.013 | 0.025 | 0.617 |
| rs1042640 | *UGT1A1* | 2:234346283 | am_13068 | 156 | 85 | 14 | 0.222 | -0.037 | 0.015 | 0.017 | 129 | 68 | 6 | 0.197 | 0.013 | 0.026 | 0.607 |
| rs8330 | *UGT1A1* | 2:234346384 | am_13070 | 143 | 95 | 17 | 0.253 | -0.033 | 0.015 | 0.029 | 123 | 71 | 9 | 0.219 | 0.017 | 0.025 | 0.480 |
| rs1395 | *SLC5A6* | 2:27278140 | am_12327 | 115 | 111 | 29 | 0.331 | 0.016 | 0.014 | 0.255 | 96 | 81 | 26 | 0.328 | -0.001 | 0.020 | 0.940 |
| rs1884725 | *XDH* | 2:31425290 | am_12370 | 154 | 82 | 19 | 0.235 | 0.040 | 0.014 | 0.004 | 130 | 64 | 8 | 0.198 | 0.014 | 0.024 | 0.569 |
| rs2295475 | *XDH* | 2:31443351 | am_12385 | 135 | 102 | 18 | 0.271 | -0.012 | 0.015 | 0.445 | 92 | 90 | 21 | 0.325 | 0.038 | 0.022 | 0.078 |
| rs1056837 | *CYP1B1* | 2:38151654 | am_12455 | 86 | 117 | 52 | 0.433 | 0.015 | 0.013 | 0.231 | 47 | 114 | 42 | 0.488 | 0.003 | 0.022 | 0.906 |
| rs1056836 | *CYP1B1* | 2:38151707 | am_12461 | 86 | 115 | 54 | 0.437 | 0.016 | 0.013 | 0.200 | 47 | 114 | 42 | 0.488 | 0.003 | 0.022 | 0.906 |
| rs3814055 | *NR1I2* | 3:120982725 | am_13207 | 90 | 126 | 39 | 0.400 | -0.005 | 0.013 | 0.728 | 76 | 101 | 26 | 0.377 | 0.031 | 0.019 | 0.106 |
| rs6785049 | *NR1I2* | 3:121016423 | am_13253 | 90 | 114 | 51 | 0.424 | -0.003 | 0.013 | 0.800 | 78 | 95 | 30 | 0.382 | -0.011 | 0.020 | 0.584 |
| rs2276707 | *NR1I2* | 3:121016843 | am_13256 | 164 | 77 | 14 | 0.206 | -0.001 | 0.015 | 0.973 | 129 | 65 | 9 | 0.204 | -0.010 | 0.025 | 0.687 |
| rs2293616 | *SLC15A2* | 3:123124383 | am_13298 | 76 | 116 | 63 | 0.475 | 0.006 | 0.012 | 0.647 | 51 | 108 | 43 | 0.480 | -0.007 | 0.020 | 0.734 |
| rs2257212 | *SLC15A2* | 3:123126494 | am_13300 | 76 | 116 | 63 | 0.475 | 0.006 | 0.012 | 0.647 | 52 | 108 | 42 | 0.475 | -0.007 | 0.020 | 0.723 |
| rs1143670 | *SLC15A2* | 3:123129331 | am_13301 | 76 | 116 | 63 | 0.475 | 0.006 | 0.012 | 0.647 | 52 | 108 | 43 | 0.478 | -0.006 | 0.019 | 0.760 |
| rs1143671 | *SLC15A2* | 3:123129976 | am_13306 | 75 | 118 | 60 | 0.470 | 0.008 | 0.013 | 0.545 | 52 | 108 | 42 | 0.475 | -0.007 | 0.020 | 0.723 |
| rs1143672 | *SLC15A2* | 3:123130858 | am_13307 | 76 | 116 | 63 | 0.475 | 0.006 | 0.012 | 0.647 | 52 | 108 | 42 | 0.475 | -0.007 | 0.020 | 0.723 |
| rs1801282 | *PPARG* | 3:12368125 | am_13097 | 209 | 43 | 3 | 0.096 | 0.008 | 0.022 | 0.697 | 142 | 57 | 4 | 0.160 | -0.018 | 0.027 | 0.511 |
| rs1152003 | *PPARG* | 3:12452055 | am_13140 | 108 | 124 | 22 | 0.331 | -0.001 | 0.014 | 0.971 | 87 | 101 | 14 | 0.319 | 0.000 | 0.024 | 0.992 |
| rs3856650 | *CHST13* | 3:127729888 | am_13323 | 90 | 132 | 33 | 0.388 | 0.032 | 0.013 | 0.018 | 67 | 93 | 43 | 0.441 | 0.022 | 0.019 | 0.244 |
| rs4305381 | *CHST13* | 3:127732567 | am_13324 | 150 | 95 | 8 | 0.219 | -0.010 | 0.017 | 0.565 | 109 | 82 | 12 | 0.261 | -0.009 | 0.022 | 0.674 |
| rs6783962 | *CHST13* | 3:127739359 | am_13325 | 112 | 118 | 25 | 0.329 | 0.008 | 0.014 | 0.567 | 78 | 89 | 36 | 0.397 | 0.016 | 0.019 | 0.384 |
| rs1873397 | *CHST13* | 3:127740562 | am_13326 | 113 | 117 | 25 | 0.327 | 0.007 | 0.014 | 0.610 | 79 | 87 | 36 | 0.394 | 0.021 | 0.019 | 0.252 |
| rs1056522 | *CHST13* | 3:127744035 | am_13333 | 132 | 105 | 18 | 0.276 | 0.005 | 0.014 | 0.725 | 95 | 87 | 21 | 0.318 | 0.008 | 0.020 | 0.699 |
| rs3755740 | *CHST2* | 3:144319656 | am_13337 | 130 | 105 | 19 | 0.281 | -0.013 | 0.015 | 0.391 | 121 | 71 | 11 | 0.229 | -0.055 | 0.022 | 0.013 |
| rs3755739 | *CHST2* | 3:144319962 | am_13338 | 132 | 102 | 21 | 0.282 | -0.012 | 0.014 | 0.420 | 120 | 72 | 11 | 0.232 | -0.057 | 0.022 | 0.010 |
| rs4683739 | *CHST2* | 3:144320051 | am_13339 | 136 | 102 | 15 | 0.261 | -0.008 | 0.015 | 0.579 | 131 | 63 | 9 | 0.200 | -0.044 | 0.023 | 0.062 |
| rs6664 | *CHST2* | 3:144324430 | am_13346 | 118 | 110 | 27 | 0.322 | -0.018 | 0.014 | 0.184 | 108 | 83 | 12 | 0.264 | -0.022 | 0.023 | 0.344 |
| rs2341970 | *SLC6A6* | 3:14449229 | am_13148 | 133 | 106 | 16 | 0.271 | 0.001 | 0.015 | 0.944 | 96 | 88 | 19 | 0.310 | 0.004 | 0.020 | 0.829 |
| rs9036 | *SLC6A6* | 3:14505725 | am_13153 | 168 | 73 | 14 | 0.198 | 0.021 | 0.015 | 0.163 | 122 | 66 | 15 | 0.236 | -0.009 | 0.021 | 0.676 |
| rs562 | *ABCC5* | 3:185120539 | am_13365 | 61 | 137 | 57 | 0.492 | 0.008 | 0.013 | 0.530 | 40 | 106 | 57 | 0.542 | 0.021 | 0.021 | 0.319 |
| rs3749442 | *ABCC5* | 3:185143279 | am_13377 | 163 | 89 | 3 | 0.186 | -0.029 | 0.017 | 0.089 | 135 | 59 | 9 | 0.190 | 0.017 | 0.026 | 0.500 |
| rs939336 | *ABCC5* | 3:185168228 | am_13393 | 69 | 143 | 43 | 0.449 | 0.002 | 0.014 | 0.866 | 60 | 104 | 39 | 0.448 | 0.005 | 0.021 | 0.797 |
| rs7636910 | *ABCC5* | 3:185182210 | am_13400 | 101 | 130 | 24 | 0.349 | 0.019 | 0.014 | 0.176 | 91 | 88 | 24 | 0.335 | -0.021 | 0.021 | 0.337 |
| rs2298419 | *SLC22A13* | 3:38297630 | am_13163 | 107 | 118 | 25 | 0.336 | 0.003 | 0.014 | 0.860 | 86 | 95 | 20 | 0.336 | -0.005 | 0.020 | 0.810 |
| rs4679028 | *SLC22A13* | 3:38302296 | am_13166 | 159 | 91 | 5 | 0.198 | 0.000 | 0.017 | 0.991 | 111 | 73 | 19 | 0.273 | 0.009 | 0.020 | 0.651 |
| rs171248 | *SLC22A14* | 3:38338397 | am_13175 | 100 | 121 | 34 | 0.371 | -0.005 | 0.014 | 0.723 | 70 | 88 | 45 | 0.438 | -0.005 | 0.019 | 0.781 |
| rs183574 | *SLC22A14* | 3:38338841 | am_13176 | 101 | 119 | 34 | 0.368 | -0.007 | 0.014 | 0.627 | 71 | 91 | 41 | 0.426 | -0.009 | 0.019 | 0.655 |
| rs149738 | *SLC22A14* | 3:38342988 | am_13180 | 102 | 120 | 33 | 0.365 | -0.005 | 0.014 | 0.715 | 70 | 88 | 45 | 0.438 | -0.005 | 0.019 | 0.781 |
| rs735320 | *CYP8B1* | 3:42890882 | am_13182 | 165 | 76 | 14 | 0.204 | 0.035 | 0.015 | 0.017 | 147 | 49 | 7 | 0.155 | -0.025 | 0.026 | 0.348 |
| rs6771233 | *CYP8B1* | 3:42897701 | am_13191 | 118 | 108 | 29 | 0.325 | -0.002 | 0.013 | 0.880 | 95 | 89 | 19 | 0.313 | 0.006 | 0.022 | 0.771 |
| rs6774801 | *CYP8B1* | 3:42898780 | am_13192 | 118 | 108 | 29 | 0.325 | -0.002 | 0.013 | 0.880 | 95 | 89 | 19 | 0.313 | 0.006 | 0.022 | 0.771 |
| rs1154400 | *ADH5* | 4:100229033 | am_13717 | 140 | 96 | 19 | 0.263 | -0.002 | 0.015 | 0.910 | 98 | 80 | 25 | 0.320 | 0.022 | 0.021 | 0.300 |
| rs2602836 | *ADH5* | 4:100233828 | am_13720 | 108 | 101 | 46 | 0.378 | 0.001 | 0.012 | 0.915 | 74 | 86 | 43 | 0.424 | 0.005 | 0.021 | 0.823 |
| rs1126672 | *ADH4* | 4:100266835 | am_13727 | 155 | 83 | 17 | 0.229 | -0.001 | 0.015 | 0.951 | 102 | 81 | 20 | 0.298 | 0.026 | 0.022 | 0.233 |
| rs1126671 | *ADH4* | 4:100267437 | am_13729 | 145 | 85 | 25 | 0.265 | 0.008 | 0.014 | 0.557 | 99 | 81 | 23 | 0.313 | 0.025 | 0.022 | 0.254 |
| rs1126670 | *ADH4* | 4:100271756 | am_13730 | 145 | 85 | 25 | 0.265 | 0.008 | 0.014 | 0.557 | 99 | 81 | 23 | 0.313 | 0.025 | 0.022 | 0.254 |
| rs3762894 | *ADH4* | 4:100285107 | am_13741 | 170 | 79 | 5 | 0.175 | -0.006 | 0.017 | 0.719 | 156 | 40 | 6 | 0.129 | 0.010 | 0.031 | 0.743 |
| rs10002894 | *ADH6* | 4:100360263 | am_13759 | 131 | 99 | 25 | 0.292 | 0.031 | 0.013 | 0.018 | 113 | 69 | 21 | 0.273 | 0.004 | 0.022 | 0.863 |
| rs10008281 | *ADH6* | 4:100361325 | am_13760 | 139 | 105 | 11 | 0.249 | -0.017 | 0.015 | 0.271 | 130 | 67 | 6 | 0.195 | 0.037 | 0.027 | 0.168 |
| rs6830685 | *ADH6* | 4:100362207 | am_13761 | 133 | 97 | 25 | 0.288 | 0.029 | 0.013 | 0.028 | 114 | 68 | 21 | 0.271 | 0.005 | 0.022 | 0.826 |
| rs6811453 | *ADH1A* | 4:100414000 | am_13763 | 100 | 131 | 24 | 0.351 | -0.019 | 0.014 | 0.167 | 71 | 93 | 39 | 0.421 | -0.023 | 0.021 | 0.283 |
| rs12512110 | *ADH1A* | 4:100414838 | am_13764 | 212 | 42 | 1 | 0.086 | -0.002 | 0.025 | 0.922 | 171 | 30 | 2 | 0.084 | -0.006 | 0.034 | 0.861 |
| rs1826909 | *ADH1A* | 4:100436766 | am_13775 | 102 | 129 | 23 | 0.344 | -0.022 | 0.014 | 0.122 | 70 | 96 | 37 | 0.419 | -0.025 | 0.022 | 0.247 |
| rs1789915 | *ADH1C* | 4:100485394 | am_13817 | 135 | 99 | 21 | 0.276 | -0.005 | 0.014 | 0.713 | 95 | 80 | 28 | 0.335 | -0.049 | 0.023 | 0.032 |
| rs4148269 | *UGT2B15* | 4:69195442 | am_13437 | 107 | 116 | 32 | 0.353 | 0.017 | 0.013 | 0.196 | 80 | 94 | 28 | 0.371 | -0.034 | 0.019 | 0.071 |
| rs1902023 | *UGT2B15* | 4:69218679 | am_13439 | 64 | 133 | 58 | 0.488 | -0.011 | 0.013 | 0.396 | 51 | 108 | 44 | 0.483 | -0.036 | 0.020 | 0.075 |
| rs3100 | *UGT2B15* | 4:69547273 | am_13442 | 125 | 98 | 32 | 0.318 | 0.010 | 0.013 | 0.449 | 87 | 88 | 28 | 0.355 | -0.036 | 0.018 | 0.049 |
| rs4148269 | *UGT2B15* | 4:69547466 | am_13444 | 107 | 116 | 32 | 0.353 | 0.017 | 0.013 | 0.196 | 80 | 95 | 28 | 0.372 | -0.034 | 0.019 | 0.071 |
| rs7662029 | *UGT2B7* | 4:69996501 | am_13458 | 84 | 104 | 67 | 0.467 | -0.021 | 0.012 | 0.096 | 58 | 95 | 50 | 0.480 | -0.018 | 0.020 | 0.378 |
| rs7668258 | *UGT2B7* | 4:69996667 | am_13459 | 84 | 104 | 67 | 0.467 | -0.021 | 0.012 | 0.096 | 58 | 95 | 49 | 0.478 | -0.018 | 0.020 | 0.370 |
| rs28365062 | *UGT2B7* | 4:69998860 | am_13463 | 204 | 45 | 6 | 0.112 | -0.037 | 0.020 | 0.058 | 160 | 37 | 5 | 0.116 | 0.000 | 0.028 | 0.991 |
| rs7438284 | *UGT2B7* | 4:69998926 | am_13464 | 83 | 104 | 67 | 0.469 | -0.020 | 0.012 | 0.106 | 58 | 95 | 50 | 0.480 | -0.018 | 0.020 | 0.378 |
| rs7439366 | *UGT2B7* | 4:69998927 | am_13465 | 83 | 104 | 67 | 0.469 | -0.020 | 0.012 | 0.106 | 58 | 95 | 50 | 0.480 | -0.018 | 0.020 | 0.378 |
| rs7697037 | *UGT2B11* | 4:70114564 | am_13478 | 213 | 36 | 6 | 0.094 | -0.029 | 0.021 | 0.165 | 176 | 24 | 3 | 0.074 | 0.006 | 0.036 | 0.866 |
| rs1131878 | *UGT2B4* | 4:70380493 | am_13507 | 109 | 112 | 34 | 0.353 | -0.002 | 0.013 | 0.870 | 85 | 94 | 24 | 0.350 | 0.004 | 0.020 | 0.825 |
| rs1966151 | *UGT2B4* | 4:70380716 | am_13509 | 147 | 92 | 16 | 0.243 | 0.000 | 0.015 | 0.983 | 125 | 71 | 7 | 0.209 | -0.016 | 0.023 | 0.468 |
| rs13142440 | *UGT2B4* | 4:70381153 | am_13511 | 137 | 103 | 15 | 0.261 | -0.012 | 0.014 | 0.406 | 99 | 88 | 16 | 0.296 | -0.005 | 0.021 | 0.804 |
| rs13119049 | *UGT2B4* | 4:70381154 | am_13513 | 137 | 103 | 15 | 0.261 | -0.012 | 0.014 | 0.406 | 99 | 88 | 16 | 0.296 | -0.005 | 0.021 | 0.804 |
| rs2288741 | *UGT2A1* | 4:70490067 | am_13534 | 137 | 108 | 10 | 0.251 | -0.015 | 0.016 | 0.332 | 141 | 58 | 4 | 0.163 | -0.009 | 0.025 | 0.708 |
| rs4148304 | *UGT2A1* | 4:70494917 | am_13535 | 191 | 58 | 6 | 0.137 | 0.020 | 0.018 | 0.249 | 124 | 71 | 7 | 0.210 | 0.012 | 0.023 | 0.601 |
| rs11249454 | *UGT2A1* | 4:70533823 | am_13558 | 184 | 66 | 5 | 0.149 | 0.019 | 0.020 | 0.361 | 162 | 38 | 3 | 0.108 | 0.024 | 0.034 | 0.483 |
| rs11731028 | *SULT1B1* | 4:70637429 | am_13600 | 93 | 125 | 37 | 0.390 | -0.010 | 0.013 | 0.435 | 96 | 87 | 20 | 0.313 | -0.013 | 0.020 | 0.507 |
| rs11249460 | *SULT1B1* | 4:70640615 | am_13602 | 83 | 127 | 45 | 0.425 | 0.010 | 0.013 | 0.457 | 71 | 104 | 28 | 0.394 | 0.050 | 0.020 | 0.014 |
| rs1604741 | *SULT1B1* | 4:70645708 | am_13603 | 65 | 141 | 49 | 0.469 | -0.005 | 0.013 | 0.680 | 59 | 107 | 37 | 0.446 | -0.031 | 0.020 | 0.113 |
| rs3822172 | *SULT1E1* | 4:70758429 | am_13632 | 187 | 60 | 8 | 0.149 | 0.042 | 0.017 | 0.015 | 128 | 74 | 1 | 0.187 | 0.016 | 0.026 | 0.551 |
| rs3775770 | *SULT1E1* | 4:70758859 | am_13634 | 124 | 102 | 29 | 0.314 | -0.007 | 0.014 | 0.607 | 97 | 89 | 17 | 0.303 | 0.029 | 0.023 | 0.207 |
| rs1881668 | *SULT1E1* | 4:70760045 | am_13635 | 150 | 86 | 19 | 0.243 | 0.028 | 0.014 | 0.052 | 93 | 90 | 20 | 0.320 | -0.010 | 0.021 | 0.617 |
| rs3736599 | *SULT1E1* | 4:70760410 | am_13637 | 208 | 41 | 6 | 0.104 | 0.025 | 0.020 | 0.214 | 144 | 58 | 1 | 0.148 | -0.001 | 0.029 | 0.970 |
| rs3756067 | *ALB* | 4:74488509 | am_13647 | 103 | 119 | 33 | 0.363 | 0.007 | 0.013 | 0.576 | 77 | 97 | 29 | 0.382 | 0.035 | 0.020 | 0.080 |
| rs2231142 | *ABCG2* | 4:89271347 | am_13688 | 211 | 40 | 4 | 0.094 | -0.005 | 0.020 | 0.798 | 167 | 35 | 1 | 0.091 | -0.032 | 0.032 | 0.310 |
| rs272893 | *SLC22A4* | 5:131690961 | am_13894 | 82 | 132 | 41 | 0.420 | 0.013 | 0.013 | 0.299 | 72 | 95 | 36 | 0.411 | 0.019 | 0.021 | 0.369 |
| rs272879 | *SLC22A4* | 5:131698445 | am_13896 | 82 | 129 | 44 | 0.425 | 0.014 | 0.013 | 0.287 | 72 | 95 | 36 | 0.411 | 0.019 | 0.021 | 0.369 |
| rs1050152 | *SLC22A4* | 5:131704219 | am_13903 | 88 | 138 | 29 | 0.384 | -0.016 | 0.014 | 0.246 | 64 | 100 | 39 | 0.438 | 0.017 | 0.021 | 0.424 |
| rs274558 | *SLC22A5* | 5:131749073 | am_13934 | 82 | 130 | 43 | 0.424 | 0.012 | 0.013 | 0.359 | 72 | 95 | 36 | 0.411 | 0.019 | 0.021 | 0.369 |
| rs1045020 | *SLC22A5* | 5:131757910 | am_13958 | 206 | 47 | 2 | 0.100 | -0.024 | 0.022 | 0.284 | 160 | 41 | 2 | 0.111 | 0.016 | 0.033 | 0.620 |
| rs274548 | *SLC22A5* | 5:131758706 | am_13959 | 177 | 71 | 7 | 0.167 | 0.003 | 0.017 | 0.871 | 151 | 47 | 5 | 0.140 | -0.055 | 0.029 | 0.057 |
| rs6196 | *NR3C1* | 5:142641683 | am_13963 | 189 | 62 | 4 | 0.137 | 0.025 | 0.019 | 0.189 | 137 | 61 | 5 | 0.175 | -0.033 | 0.027 | 0.232 |
| rs5909 | *HMGCR* | 5:74691931 | am_13871 | 206 | 47 | 2 | 0.100 | -0.016 | 0.023 | 0.473 | 166 | 33 | 4 | 0.101 | 0.028 | 0.030 | 0.349 |
| rs1867351 | *SLC22A1* | 6:160463113 | am_14347 | 152 | 98 | 4 | 0.209 | 0.028 | 0.017 | 0.100 | 130 | 66 | 6 | 0.193 | -0.027 | 0.025 | 0.273 |
| rs683369 | *SLC22A1* | 6:160471194 | am_14351 | 156 | 87 | 12 | 0.218 | 0.010 | 0.015 | 0.535 | 127 | 70 | 6 | 0.202 | -0.002 | 0.025 | 0.942 |
| rs628031 | *SLC22A1* | 6:160480835 | am_14363 | 109 | 107 | 39 | 0.363 | -0.004 | 0.013 | 0.774 | 65 | 110 | 28 | 0.409 | 0.009 | 0.023 | 0.705 |
| rs72552763 | *SLC22A1* | 6:160480873 | am_14368 | 169 | 75 | 10 | 0.187 | 0.032 | 0.016 | 0.048 | 123 | 72 | 8 | 0.217 | 0.028 | 0.026 | 0.287 |
| rs316003 | *SLC22A2* | 6:160565822 | am_14386 | 150 | 95 | 10 | 0.225 | 0.012 | 0.016 | 0.475 | 130 | 64 | 9 | 0.202 | -0.011 | 0.025 | 0.661 |
| rs316019 | *SLC22A2* | 6:160590272 | am_14395 | 200 | 51 | 4 | 0.116 | 0.014 | 0.020 | 0.483 | 160 | 40 | 3 | 0.113 | -0.036 | 0.034 | 0.299 |
| rs624249 | *SLC22A2* | 6:160599390 | am_14401 | 92 | 129 | 34 | 0.386 | -0.001 | 0.014 | 0.965 | 52 | 101 | 50 | 0.495 | -0.020 | 0.020 | 0.317 |
| rs668871 | *SLC22A3* | 6:160689801 | am_14410 | 69 | 136 | 47 | 0.456 | 0.003 | 0.014 | 0.826 | 42 | 108 | 53 | 0.527 | -0.013 | 0.020 | 0.506 |
| rs2292334 | *SLC22A3* | 6:160778178 | am_14444 | 92 | 129 | 34 | 0.386 | -0.001 | 0.014 | 0.949 | 93 | 95 | 15 | 0.308 | 0.005 | 0.022 | 0.817 |
| rs2842934 | *TPMT* | 6:18247193 | am_13979 | 155 | 82 | 17 | 0.228 | -0.006 | 0.015 | 0.685 | 106 | 80 | 17 | 0.281 | 0.029 | 0.022 | 0.182 |
| rs2267665 | *PPARD* | 6:35477469 | am_14131 | 177 | 72 | 5 | 0.161 | -0.013 | 0.019 | 0.507 | 142 | 56 | 5 | 0.163 | -0.048 | 0.032 | 0.130 |
| rs1883322 | *PPARD* | 6:35477784 | am_14132 | 132 | 106 | 17 | 0.275 | -0.012 | 0.021 | 0.552 | 119 | 69 | 15 | 0.244 | -0.025 | 0.049 | 0.612 |
| rs7751481 | *PPARD* | 6:35479731 | am_14134 | 132 | 106 | 17 | 0.275 | -0.012 | 0.021 | 0.552 | 119 | 69 | 15 | 0.244 | -0.025 | 0.049 | 0.612 |
| rs2267667 | *PPARD* | 6:35480502 | am_14135 | 132 | 105 | 17 | 0.274 | -0.012 | 0.021 | 0.549 | 119 | 69 | 15 | 0.244 | -0.025 | 0.049 | 0.612 |
| rs2038067 | *PPARD* | 6:35482444 | am_14136 | 177 | 73 | 5 | 0.163 | -0.011 | 0.019 | 0.567 | 142 | 56 | 5 | 0.163 | -0.048 | 0.032 | 0.130 |
| rs1003973 | *PPARD* | 6:35485279 | am_14145 | 175 | 75 | 5 | 0.167 | -0.011 | 0.019 | 0.561 | 142 | 56 | 5 | 0.163 | -0.048 | 0.032 | 0.130 |
| rs2267668 | *PPARD* | 6:35485900 | am_14146 | 177 | 73 | 5 | 0.163 | -0.011 | 0.019 | 0.567 | 142 | 56 | 5 | 0.163 | -0.048 | 0.032 | 0.130 |
| rs2267669 | *PPARD* | 6:35486102 | am_14147 | 177 | 73 | 5 | 0.163 | -0.011 | 0.019 | 0.567 | 142 | 56 | 5 | 0.163 | -0.048 | 0.032 | 0.130 |
| rs2076167 | *PPARD* | 6:35499765 | am_14149 | 133 | 106 | 16 | 0.271 | -0.009 | 0.021 | 0.677 | 125 | 67 | 11 | 0.219 | 0.008 | 0.046 | 0.870 |
| rs2270860 | *SLC22A7* | 6:43378129 | am_14163 | 114 | 123 | 17 | 0.309 | -0.010 | 0.015 | 0.471 | 104 | 88 | 11 | 0.271 | -0.022 | 0.022 | 0.325 |
| rs2242416 | *SLC22A7* | 6:43381582 | am_14166 | 87 | 117 | 51 | 0.429 | 0.001 | 0.012 | 0.919 | 73 | 105 | 25 | 0.382 | -0.036 | 0.020 | 0.067 |
| rs7761731 | *CYP39A1* | 6:46671776 | am_14196 | 139 | 104 | 12 | 0.251 | 0.002 | 0.015 | 0.911 | 130 | 68 | 5 | 0.192 | -0.006 | 0.024 | 0.809 |
| rs2277119 | *CYP39A1* | 6:46717864 | am_14216 | 130 | 109 | 14 | 0.271 | -0.014 | 0.014 | 0.330 | 118 | 73 | 12 | 0.239 | 0.018 | 0.025 | 0.454 |
| rs9381468 | *CYP39A1* | 6:46733233 | am_14226 | 53 | 131 | 67 | 0.528 | 0.003 | 0.014 | 0.826 | 58 | 90 | 48 | 0.474 | -0.009 | 0.021 | 0.659 |
| rs953062 | *CYP39A1* | 6:46734312 | am_14227 | 109 | 111 | 35 | 0.355 | 0.002 | 0.013 | 0.861 | 112 | 74 | 16 | 0.262 | -0.004 | 0.022 | 0.857 |
| rs2180314 | *GSTA2* | 6:52725690 | am_14240 | 99 | 114 | 42 | 0.388 | 0.017 | 0.013 | 0.181 | 69 | 109 | 25 | 0.392 | 0.016 | 0.019 | 0.389 |
| rs4715332 | *GSTA1* | 6:52777144 | am_14286 | 87 | 120 | 48 | 0.424 | 0.008 | 0.013 | 0.523 | 81 | 96 | 26 | 0.365 | 0.023 | 0.018 | 0.204 |
| rs4715333 | *GSTA1* | 6:52777208 | am_14287 | 103 | 104 | 37 | 0.365 | -0.007 | 0.014 | 0.616 | 77 | 89 | 27 | 0.370 | -0.007 | 0.019 | 0.725 |
| rs4715354 | *GSTA5* | 6:52816756 | am_14296 | 51 | 132 | 71 | 0.539 | -0.004 | 0.014 | 0.787 | 47 | 115 | 40 | 0.483 | 0.012 | 0.018 | 0.522 |
| rs512795 | *GSTA3* | 6:52874942 | am_14306 | 221 | 33 | 1 | 0.069 | -0.050 | 0.024 | 0.041 | 173 | 29 | 1 | 0.076 | 0.054 | 0.035 | 0.121 |
| rs405729 | *GSTA4* | 6:52950740 | am_14321 | 91 | 120 | 43 | 0.406 | -0.006 | 0.013 | 0.670 | 52 | 95 | 56 | 0.510 | 0.044 | 0.019 | 0.021 |
| rs7496 | *GSTA4* | 6:52950798 | am_14322 | 201 | 49 | 4 | 0.112 | -0.013 | 0.020 | 0.510 | 165 | 35 | 3 | 0.101 | -0.010 | 0.034 | 0.776 |
| rs367836 | *GSTA4* | 6:52951090 | am_14324 | 104 | 122 | 29 | 0.353 | -0.008 | 0.013 | 0.553 | 71 | 90 | 42 | 0.429 | 0.026 | 0.020 | 0.196 |
| rs13197674 | *GSTA4* | 6:52968366 | am_14336 | 110 | 123 | 22 | 0.327 | -0.015 | 0.014 | 0.295 | 84 | 93 | 25 | 0.354 | 0.040 | 0.021 | 0.059 |
| rs6962039 | *SLC13A1* | 7:122541594 | am_14871 | 124 | 113 | 17 | 0.289 | 0.000 | 0.014 | 0.977 | 105 | 80 | 18 | 0.286 | -0.015 | 0.022 | 0.506 |
| rs2140516 | *SLC13A1* | 7:122596470 | am_14896 | 131 | 102 | 22 | 0.286 | 0.002 | 0.014 | 0.895 | 99 | 84 | 19 | 0.302 | 0.008 | 0.023 | 0.726 |
| rs2204295 | *SLC13A1* | 7:122625326 | am_14912 | 98 | 123 | 33 | 0.372 | -0.001 | 0.014 | 0.932 | 84 | 99 | 20 | 0.342 | -0.020 | 0.021 | 0.323 |
| rs1880179 | *SLC13A1* | 7:122625869 | am_14913 | 98 | 122 | 35 | 0.376 | -0.001 | 0.013 | 0.968 | 83 | 100 | 20 | 0.345 | -0.010 | 0.021 | 0.615 |
| rs10893 | *ABP1* | 7:150186848 | am_14950 | 126 | 106 | 23 | 0.298 | 0.021 | 0.014 | 0.126 | 89 | 93 | 21 | 0.333 | 0.025 | 0.020 | 0.226 |
| rs4725373 | *ABP1* | 7:150188555 | am_14953 | 126 | 106 | 23 | 0.298 | 0.021 | 0.014 | 0.126 | 89 | 93 | 21 | 0.333 | 0.025 | 0.020 | 0.226 |
| rs1049793 | *ABP1* | 7:150188598 | am_14954 | 126 | 105 | 23 | 0.297 | 0.021 | 0.014 | 0.130 | 89 | 93 | 21 | 0.333 | 0.025 | 0.020 | 0.226 |
| rs12539 | *ABP1* | 7:150189299 | am_14955 | 147 | 95 | 13 | 0.237 | -0.027 | 0.015 | 0.071 | 139 | 56 | 8 | 0.177 | -0.014 | 0.027 | 0.608 |
| rs10249788 | *AHR* | 7:17304672 | am_14464 | 188 | 62 | 5 | 0.141 | 0.008 | 0.018 | 0.655 | 161 | 41 | 1 | 0.106 | -0.087 | 0.036 | 0.016 |
| rs6965343 | *POR* | 7:75430861 | am_14490 | 90 | 118 | 47 | 0.416 | 0.024 | 0.013 | 0.062 | 82 | 91 | 30 | 0.372 | -0.031 | 0.020 | 0.133 |
| rs17685 | *POR* | 7:75454041 | am_14518 | 128 | 113 | 14 | 0.276 | 0.003 | 0.016 | 0.858 | 89 | 95 | 19 | 0.328 | -0.015 | 0.021 | 0.497 |
| rs31651 | *CROT* | 7:86867223 | am_14526 | 198 | 49 | 7 | 0.124 | 0.021 | 0.019 | 0.249 | 166 | 34 | 3 | 0.099 | -0.011 | 0.035 | 0.755 |
| rs31652 | *CROT* | 7:86867623 | am_14527 | 191 | 54 | 9 | 0.142 | 0.021 | 0.018 | 0.242 | 161 | 38 | 4 | 0.113 | -0.042 | 0.033 | 0.211 |
| rs2097937 | *CROT* | 7:86868839 | am_14528 | 165 | 75 | 15 | 0.206 | 0.016 | 0.016 | 0.311 | 144 | 50 | 9 | 0.167 | 0.013 | 0.029 | 0.643 |
| rs2109505 | *ABCB4* | 7:86917342 | am_14553 | 155 | 87 | 12 | 0.219 | -0.011 | 0.016 | 0.477 | 141 | 56 | 6 | 0.167 | 0.000 | 0.028 | 0.988 |
| rs1202283 | *ABCB4* | 7:86920228 | am_14556 | 60 | 130 | 65 | 0.510 | -0.004 | 0.013 | 0.788 | 45 | 102 | 56 | 0.527 | 0.001 | 0.021 | 0.950 |
| rs2302387 | *ABCB4* | 7:86930121 | am_14562 | 176 | 69 | 10 | 0.175 | -0.010 | 0.016 | 0.522 | 152 | 49 | 2 | 0.131 | -0.013 | 0.030 | 0.671 |
| rs4148808 | *ABCB4* | 7:86943731 | am_14567 | 182 | 63 | 10 | 0.163 | -0.016 | 0.016 | 0.324 | 152 | 49 | 2 | 0.131 | -0.013 | 0.030 | 0.671 |
| rs4148805 | *ABCB4* | 7:86944301 | am_14570 | 179 | 65 | 10 | 0.167 | -0.016 | 0.016 | 0.338 | 152 | 49 | 2 | 0.131 | -0.013 | 0.030 | 0.671 |
| rs3842 | *ABCB1* | 7:86971302 | am_14575 | 176 | 68 | 10 | 0.173 | -0.013 | 0.016 | 0.430 | 155 | 46 | 2 | 0.123 | -0.018 | 0.031 | 0.562 |
| rs17064 | *ABCB1* | 7:86971406 | am_14577 | 220 | 34 | 1 | 0.071 | -0.010 | 0.024 | 0.690 | 174 | 28 | 1 | 0.074 | 0.001 | 0.040 | 0.986 |
| rs1045642 | *ABCB1* | 7:86976581 | am_14581 | 67 | 140 | 48 | 0.463 | 0.010 | 0.014 | 0.472 | 51 | 107 | 45 | 0.485 | 0.018 | 0.022 | 0.427 |
| rs2235040 | *ABCB1* | 7:87003686 | am_14598 | 187 | 66 | 2 | 0.137 | 0.023 | 0.020 | 0.244 | 164 | 37 | 2 | 0.101 | 0.051 | 0.031 | 0.105 |
| rs2235013 | *ABCB1* | 7:87016562 | am_14605 | 74 | 131 | 50 | 0.453 | 0.016 | 0.014 | 0.253 | 43 | 109 | 51 | 0.520 | 0.009 | 0.022 | 0.677 |
| rs2235033 | *ABCB1* | 7:87017079 | am_14609 | 74 | 131 | 50 | 0.453 | 0.016 | 0.014 | 0.253 | 43 | 108 | 51 | 0.520 | 0.008 | 0.022 | 0.704 |
| rs2032588 | *ABCB1* | 7:87017379 | am_14610 | 226 | 27 | 2 | 0.061 | 0.019 | 0.024 | 0.433 | 174 | 28 | 1 | 0.074 | -0.005 | 0.041 | 0.895 |
| rs1128503 | *ABCB1* | 7:87017537 | am_14612 | 69 | 135 | 51 | 0.465 | -0.019 | 0.014 | 0.177 | 77 | 97 | 29 | 0.382 | 0.000 | 0.024 | 0.995 |
| rs10276036 | *ABCB1* | 7:87018134 | am_14617 | 66 | 137 | 51 | 0.470 | -0.018 | 0.015 | 0.228 | 77 | 97 | 29 | 0.382 | 0.000 | 0.024 | 0.995 |
| rs2235015 | *ABCB1* | 7:87037500 | am_14624 | 157 | 90 | 6 | 0.202 | 0.031 | 0.017 | 0.067 | 139 | 51 | 13 | 0.190 | 0.032 | 0.026 | 0.205 |
| rs7785971 | *AKAP9* | 7:91574730 | am_14661 | 88 | 105 | 62 | 0.449 | 0.011 | 0.012 | 0.328 | 59 | 106 | 38 | 0.448 | -0.019 | 0.020 | 0.345 |
| rs2049900 | *AKAP9* | 7:91576724 | am_14662 | 215 | 35 | 5 | 0.088 | 0.007 | 0.021 | 0.739 | 171 | 31 | 1 | 0.081 | 0.069 | 0.036 | 0.058 |
| rs7793861 | *CYP51A1* | 7:91580664 | am_14666 | 87 | 103 | 61 | 0.448 | 0.014 | 0.012 | 0.235 | 59 | 106 | 38 | 0.448 | -0.019 | 0.020 | 0.345 |
| rs7797834 | *CYP51A1* | 7:91581086 | am_14667 | 90 | 103 | 62 | 0.445 | 0.012 | 0.012 | 0.325 | 59 | 106 | 38 | 0.448 | -0.019 | 0.020 | 0.345 |
| rs854560 | *PON1* | 7:94784020 | am_14681 | 99 | 142 | 14 | 0.333 | 0.021 | 0.015 | 0.178 | 77 | 99 | 27 | 0.377 | 0.014 | 0.020 | 0.490 |
| rs13226149 | *PON3* | 7:94863536 | am_14702 | 132 | 102 | 21 | 0.282 | -0.008 | 0.014 | 0.539 | 110 | 78 | 15 | 0.266 | 0.009 | 0.023 | 0.691 |
| rs2072200 | *PON3* | 7:94864096 | am_14704 | 172 | 76 | 7 | 0.176 | 0.019 | 0.017 | 0.260 | 120 | 77 | 6 | 0.219 | 0.039 | 0.024 | 0.114 |
| rs11764079 | *PON3* | 7:94864165 | am_14705 | 130 | 104 | 21 | 0.286 | -0.008 | 0.014 | 0.572 | 110 | 78 | 15 | 0.266 | 0.009 | 0.023 | 0.691 |
| rs11770903 | *PON3* | 7:94864263 | am_14706 | 130 | 104 | 21 | 0.286 | -0.008 | 0.014 | 0.572 | 109 | 78 | 15 | 0.267 | 0.008 | 0.023 | 0.726 |
| rs17882539 | *PON3* | 7:94864344 | am_14707 | 129 | 104 | 21 | 0.287 | -0.005 | 0.014 | 0.726 | 110 | 78 | 15 | 0.266 | 0.009 | 0.023 | 0.691 |
| rs533486 | *CYP3A43* | 7:99278630 | am_14856 | 85 | 110 | 59 | 0.449 | 0.012 | 0.012 | 0.340 | 71 | 87 | 45 | 0.436 | -0.007 | 0.020 | 0.718 |
| rs4736312 | *CYP11B1* | 8:143950939 | am_15210 | 104 | 106 | 45 | 0.384 | -0.016 | 0.012 | 0.206 | 71 | 93 | 39 | 0.421 | -0.025 | 0.020 | 0.211 |
| rs1134095 | *CYP11B1* | 8:143951292 | am_15211 | 104 | 106 | 45 | 0.384 | -0.016 | 0.012 | 0.206 | 69 | 108 | 26 | 0.394 | -0.007 | 0.020 | 0.723 |
| rs7003319 | *CYP11B1* | 8:143951749 | am_15212 | 104 | 106 | 45 | 0.384 | -0.016 | 0.012 | 0.206 | 69 | 108 | 26 | 0.394 | -0.007 | 0.020 | 0.723 |
| rs5303 | *CYP11B1* | 8:143952097 | am_15217 | 104 | 106 | 45 | 0.384 | -0.016 | 0.012 | 0.206 | 69 | 108 | 26 | 0.394 | -0.007 | 0.020 | 0.723 |
| rs5297 | *CYP11B1* | 8:143952659 | am_15226 | 216 | 36 | 2 | 0.079 | -0.009 | 0.025 | 0.732 | 174 | 27 | 2 | 0.076 | -0.015 | 0.036 | 0.671 |
| rs4543 | *CYP11B2* | 8:143992745 | am_15259 | 217 | 36 | 2 | 0.078 | -0.016 | 0.025 | 0.531 | 174 | 27 | 2 | 0.076 | -0.015 | 0.036 | 0.671 |
| rs4986993 | *NAT1* | 8:18125027 | am_14993 | 131 | 94 | 30 | 0.302 | -0.001 | 0.013 | 0.941 | 124 | 72 | 7 | 0.212 | 0.037 | 0.025 | 0.139 |
| rs1041983 | *NAT2* | 8:18302075 | am_15000 | 123 | 105 | 27 | 0.312 | -0.005 | 0.014 | 0.695 | 92 | 88 | 23 | 0.330 | -0.003 | 0.022 | 0.882 |
| rs1801280 | *NAT2* | 8:18302134 | am_15001 | 78 | 120 | 57 | 0.459 | 0.000 | 0.013 | 0.992 | 65 | 94 | 44 | 0.448 | -0.026 | 0.020 | 0.193 |
| rs1799929 | *NAT2* | 8:18302274 | am_15006 | 89 | 115 | 51 | 0.425 | 0.000 | 0.012 | 0.994 | 65 | 97 | 41 | 0.441 | -0.022 | 0.020 | 0.274 |
| rs1799930 | *NAT2* | 8:18302383 | am_15008 | 135 | 102 | 18 | 0.271 | -0.004 | 0.014 | 0.799 | 99 | 87 | 17 | 0.298 | -0.003 | 0.023 | 0.898 |
| rs1208 | *NAT2* | 8:18302596 | am_15010 | 81 | 122 | 52 | 0.443 | 0.007 | 0.013 | 0.576 | 69 | 108 | 26 | 0.394 | -0.007 | 0.020 | 0.723 |
| rs41507953 | *EPHX2* | 8:27414422 | am_15017 | 209 | 36 | 10 | 0.110 | 0.010 | 0.019 | 0.613 | 165 | 34 | 3 | 0.099 | 0.020 | 0.032 | 0.533 |
| rs8192879 | *CYP7A1* | 8:59566130 | am_15037 | 81 | 117 | 57 | 0.453 | 0.020 | 0.014 | 0.159 | 68 | 96 | 38 | 0.426 | -0.038 | 0.023 | 0.095 |
| rs3808607 | *CYP7A1* | 8:59575478 | am_15047 | 114 | 105 | 36 | 0.347 | -0.031 | 0.015 | 0.036 | 89 | 92 | 22 | 0.335 | 0.087 | 0.020 | 0.000 |
| rs12542233 | *CYP7A1* | 8:59576955 | am_15048 | 108 | 109 | 38 | 0.363 | -0.031 | 0.015 | 0.036 | 85 | 95 | 23 | 0.347 | 0.083 | 0.019 | 0.000 |
| rs13251066 | *CYP7A1* | 8:59580307 | am_15050 | 78 | 120 | 56 | 0.457 | 0.027 | 0.014 | 0.055 | 69 | 95 | 39 | 0.426 | -0.046 | 0.023 | 0.044 |
| rs13265049 | *CYP7B1* | 8:65864694 | am_15085 | 198 | 53 | 3 | 0.116 | 0.001 | 0.020 | 0.969 | 148 | 52 | 2 | 0.139 | 0.014 | 0.032 | 0.659 |
| rs6987861 | *CYP7B1* | 8:65872021 | am_15086 | 168 | 81 | 6 | 0.182 | -0.017 | 0.016 | 0.295 | 123 | 78 | 2 | 0.202 | 0.006 | 0.028 | 0.843 |
| rs6980478 | *CYP7B1* | 8:65883288 | am_15090 | 200 | 52 | 3 | 0.114 | 0.001 | 0.020 | 0.973 | 146 | 55 | 2 | 0.145 | 0.020 | 0.032 | 0.530 |
| rs1138541 | *SLCO5A1* | 8:70747182 | am_15094 | 135 | 107 | 13 | 0.261 | -0.027 | 0.016 | 0.099 | 112 | 76 | 15 | 0.261 | -0.018 | 0.023 | 0.439 |
| rs16936279 | *SLCO5A1* | 8:70747363 | am_15095 | 171 | 80 | 4 | 0.173 | -0.009 | 0.018 | 0.630 | 146 | 52 | 5 | 0.153 | -0.060 | 0.029 | 0.035 |
| rs2380563 | *SLCO5A1* | 8:70767626 | am_15113 | 217 | 36 | 2 | 0.078 | -0.017 | 0.025 | 0.490 | 179 | 22 | 2 | 0.064 | -0.020 | 0.036 | 0.581 |
| rs3750266 | *SLCO5A1* | 8:70907366 | am_15200 | 82 | 130 | 41 | 0.419 | -0.026 | 0.013 | 0.052 | 66 | 108 | 29 | 0.409 | -0.031 | 0.022 | 0.147 |
| rs3750268 | *SLCO5A1* | 8:70907725 | am_15202 | 99 | 137 | 17 | 0.338 | -0.022 | 0.015 | 0.127 | 100 | 86 | 17 | 0.296 | -0.007 | 0.023 | 0.776 |
| rs1805343 | *RXRA* | 9:136468107 | am_15349 | 123 | 103 | 28 | 0.313 | 0.006 | 0.013 | 0.634 | 62 | 96 | 45 | 0.458 | 0.019 | 0.020 | 0.351 |
| rs13959 | *ALDH1A1* | 9:74735702 | am_15285 | 55 | 133 | 67 | 0.524 | -0.006 | 0.013 | 0.642 | 147 | 51 | 5 | 0.150 | -0.019 | 0.026 | 0.463 |
| rs7853758 | *SLC28A3* | 9:86090746 | am_15297 | 184 | 61 | 10 | 0.159 | -0.025 | 0.017 | 0.132 | 95 | 91 | 17 | 0.308 | 0.011 | 0.020 | 0.591 |
| rs7867504 | *SLC28A3* | 9:86110056 | am_15307 | 124 | 103 | 28 | 0.312 | -0.016 | 0.013 | 0.238 | 76 | 103 | 24 | 0.372 | -0.004 | 0.022 | 0.841 |
| rs717620 | *ABCC2* | 10:101532568 | am_10143 | 162 | 83 | 10 | 0.202 | -0.003 | 0.016 | 0.833 | 119 | 73 | 11 | 0.234 | -0.012 | 0.023 | 0.614 |
| rs2273697 | *ABCC2* | 10:101553805 | am_10152 | 178 | 64 | 13 | 0.176 | 0.010 | 0.016 | 0.538 | 145 | 54 | 4 | 0.153 | -0.004 | 0.026 | 0.869 |
| rs3740066 | *ABCC2* | 10:101594197 | am_10183 | 104 | 123 | 28 | 0.351 | 0.009 | 0.014 | 0.501 | 81 | 98 | 24 | 0.360 | -0.007 | 0.020 | 0.731 |
| rs6163 | *CYP17A1* | 10:104586914 | am_10201 | 86 | 132 | 37 | 0.404 | 0.015 | 0.014 | 0.281 | 76 | 99 | 28 | 0.382 | 0.009 | 0.020 | 0.669 |
| rs6162 | *CYP17A1* | 10:104586971 | am_10202 | 85 | 127 | 43 | 0.418 | 0.010 | 0.013 | 0.462 | 70 | 102 | 31 | 0.404 | 0.010 | 0.020 | 0.606 |
| rs743572 | *CYP17A1* | 10:104587142 | am_10206 | 86 | 132 | 37 | 0.404 | 0.015 | 0.014 | 0.281 | 76 | 99 | 28 | 0.382 | 0.009 | 0.020 | 0.669 |
| rs4925 | *GSTO1* | 10:106012779 | am_10221 | 95 | 128 | 32 | 0.376 | -0.001 | 0.014 | 0.929 | 101 | 87 | 15 | 0.288 | -0.006 | 0.021 | 0.755 |
| rs2070673 | *CYP2E1* | 10:135190557 | am_10243 | 167 | 76 | 12 | 0.196 | -0.021 | 0.016 | 0.183 | 136 | 62 | 5 | 0.177 | 0.009 | 0.028 | 0.742 |
| rs2515641 | *CYP2E1* | 10:135201352 | am_10258 | 187 | 62 | 6 | 0.145 | -0.019 | 0.018 | 0.293 | 145 | 56 | 2 | 0.148 | 0.018 | 0.030 | 0.556 |
| rs4148943 | *CHST3* | 10:73439513 | am_10003 | 71 | 145 | 38 | 0.435 | -0.002 | 0.016 | 0.890 | 49 | 100 | 54 | 0.512 | 0.026 | 0.023 | 0.255 |
| rs4148945 | *CHST3* | 10:73439596 | am_10005 | 75 | 142 | 38 | 0.427 | 0.002 | 0.016 | 0.893 | 49 | 100 | 54 | 0.512 | 0.026 | 0.023 | 0.255 |
| rs4148946 | *CHST3* | 10:73440079 | am_10006 | 63 | 147 | 45 | 0.465 | -0.011 | 0.015 | 0.460 | 78 | 92 | 33 | 0.389 | -0.021 | 0.023 | 0.356 |
| rs4148949 | *CHST3* | 10:73440657 | am_10010 | 63 | 147 | 45 | 0.465 | -0.011 | 0.015 | 0.460 | 78 | 92 | 33 | 0.389 | -0.021 | 0.023 | 0.356 |
| rs4148950 | *CHST3* | 10:73441712 | am_10011 | 73 | 144 | 38 | 0.431 | 0.003 | 0.016 | 0.856 | 48 | 101 | 54 | 0.515 | 0.019 | 0.022 | 0.408 |
| rs1871450 | *CHST3* | 10:73442020 | am_10013 | 73 | 143 | 38 | 0.431 | 0.003 | 0.016 | 0.861 | 48 | 101 | 54 | 0.515 | 0.019 | 0.022 | 0.408 |
| rs731027 | *CHST3* | 10:73442342 | am_10014 | 71 | 146 | 38 | 0.435 | -0.002 | 0.016 | 0.885 | 48 | 101 | 54 | 0.515 | 0.019 | 0.022 | 0.408 |
| rs730720 | *CHST3* | 10:73442768 | am_10016 | 71 | 146 | 38 | 0.435 | -0.002 | 0.016 | 0.885 | 48 | 101 | 54 | 0.515 | 0.019 | 0.022 | 0.408 |
| rs7087728 | *MAT1A* | 10:82023450 | am_10020 | 159 | 81 | 15 | 0.218 | -0.026 | 0.015 | 0.081 | 125 | 71 | 7 | 0.209 | -0.028 | 0.025 | 0.263 |
| rs9285726 | *MAT1A* | 10:82025130 | am_10023 | 118 | 115 | 22 | 0.312 | 0.007 | 0.014 | 0.624 | 98 | 89 | 16 | 0.298 | -0.019 | 0.020 | 0.361 |
| rs17102596 | *MAT1A* | 10:82025153 | am_10024 | 159 | 81 | 15 | 0.218 | -0.026 | 0.015 | 0.081 | 125 | 71 | 7 | 0.209 | -0.028 | 0.025 | 0.263 |
| rs4934027 | *MAT1A* | 10:82025540 | am_10025 | 163 | 78 | 14 | 0.208 | 0.005 | 0.016 | 0.755 | 122 | 76 | 5 | 0.212 | 0.014 | 0.025 | 0.571 |
| rs4418728 | *CYP26A1* | 10:94829714 | am_10031 | 71 | 120 | 64 | 0.486 | -0.006 | 0.012 | 0.653 | 62 | 99 | 42 | 0.451 | -0.015 | 0.019 | 0.431 |
| rs7905939 | *CYP26A1* | 10:94831097 | am_10033 | 178 | 67 | 10 | 0.171 | 0.023 | 0.016 | 0.157 | 137 | 56 | 10 | 0.187 | 0.053 | 0.027 | 0.056 |
| rs10882140 | *CYP26A1* | 10:94832203 | am_10035 | 89 | 106 | 60 | 0.443 | 0.020 | 0.012 | 0.094 | 61 | 100 | 42 | 0.453 | 0.010 | 0.019 | 0.572 |
| rs2281891 | *CYP2C18* | 10:96483048 | am_10047 | 201 | 51 | 3 | 0.112 | -0.003 | 0.020 | 0.884 | 120 | 73 | 10 | 0.229 | -0.006 | 0.021 | 0.781 |
| rs2860840 | *CYP2C18* | 10:96485222 | am_10048 | 110 | 107 | 38 | 0.359 | 0.022 | 0.013 | 0.079 | 79 | 100 | 24 | 0.365 | 0.021 | 0.020 | 0.302 |
| rs12248560 | *CYP2C19* | 10:96511647 | am_10053 | 142 | 92 | 21 | 0.263 | -0.028 | 0.014 | 0.045 | 128 | 63 | 12 | 0.214 | -0.013 | 0.024 | 0.605 |
| rs4244285 | *CYP2C19* | 10:96531606 | am_10070 | 201 | 51 | 3 | 0.112 | -0.003 | 0.020 | 0.884 | 123 | 68 | 12 | 0.227 | -0.003 | 0.021 | 0.880 |
| rs1799853 | *CYP2C9* | 10:96692037 | am_10100 | 182 | 69 | 4 | 0.151 | 0.017 | 0.019 | 0.363 | 148 | 50 | 4 | 0.144 | -0.006 | 0.021 | 0.784 |
| rs2852425 | *NNMT* | 11:113687717 | am_10473 | 144 | 89 | 22 | 0.261 | 0.014 | 0.014 | 0.301 | 120 | 71 | 12 | 0.234 | 0.005 | 0.023 | 0.836 |
| rs757110 | *ABCC8* | 11:17375053 | am_10270 | 110 | 95 | 50 | 0.382 | 0.012 | 0.012 | 0.348 | 80 | 100 | 23 | 0.360 | 0.014 | 0.020 | 0.465 |
| rs895729 | *CHST1* | 11:45635773 | am_10321 | 97 | 118 | 40 | 0.388 | -0.003 | 0.013 | 0.823 | 71 | 101 | 31 | 0.401 | 0.019 | 0.021 | 0.368 |
| rs2028985 | *CHST1* | 11:45651938 | am_10330 | 234 | 18 | 3 | 0.047 | -0.045 | 0.025 | 0.077 | 170 | 31 | 2 | 0.086 | -0.002 | 0.038 | 0.968 |
| rs9787901 | *CHST1* | 11:45652729 | am_10331 | 193 | 57 | 5 | 0.131 | 0.012 | 0.018 | 0.526 | 161 | 35 | 6 | 0.116 | 0.002 | 0.028 | 0.942 |
| rs750398 | *CHST1* | 11:45653648 | am_10333 | 95 | 122 | 38 | 0.388 | -0.008 | 0.014 | 0.545 | 59 | 107 | 37 | 0.446 | 0.013 | 0.021 | 0.519 |
| rs2276299 | *SLC22A8* | 11:62523007 | am_10361 | 175 | 77 | 3 | 0.163 | -0.005 | 0.019 | 0.786 | 149 | 52 | 2 | 0.138 | -0.056 | 0.034 | 0.102 |
| rs1783811 | *SLC22A11* | 11:64089872 | am_10389 | 119 | 114 | 22 | 0.310 | -0.002 | 0.014 | 0.863 | 90 | 89 | 24 | 0.337 | 0.001 | 0.022 | 0.969 |
| rs2078267 | *SLC22A11* | 11:64090690 | am_10390 | 58 | 131 | 66 | 0.516 | 0.003 | 0.013 | 0.782 | 55 | 92 | 56 | 0.502 | 0.000 | 0.021 | 0.989 |
| rs11231825 | *SLC22A12* | 11:64116850 | am_10401 | 119 | 112 | 24 | 0.314 | -0.011 | 0.014 | 0.427 | 96 | 88 | 19 | 0.310 | 0.006 | 0.021 | 0.779 |
| rs1695 | *GSTP1* | 11:67109265 | am_10440 | 103 | 109 | 43 | 0.382 | 0.027 | 0.013 | 0.040 | 101 | 79 | 23 | 0.308 | 0.013 | 0.020 | 0.515 |
| rs1138272 | *GSTP1* | 11:67110155 | am_10442 | 219 | 34 | 2 | 0.075 | 0.018 | 0.024 | 0.459 | 176 | 26 | 1 | 0.069 | -0.054 | 0.042 | 0.194 |
| rs2468110 | *CHST11* | 12:103676521 | am_10573 | 117 | 121 | 17 | 0.304 | 0.030 | 0.014 | 0.033 | 96 | 95 | 12 | 0.293 | -0.023 | 0.023 | 0.326 |
| rs2463018 | *CHST11* | 12:103677127 | am_10575 | 122 | 120 | 13 | 0.286 | 0.033 | 0.014 | 0.022 | 101 | 93 | 9 | 0.273 | -0.016 | 0.024 | 0.507 |
| rs903247 | *CHST11* | 12:103677700 | am_10576 | 115 | 120 | 20 | 0.314 | 0.028 | 0.014 | 0.043 | 95 | 93 | 14 | 0.300 | -0.013 | 0.023 | 0.573 |
| rs2463437 | *CHST11* | 12:103678217 | am_10577 | 121 | 119 | 15 | 0.292 | 0.028 | 0.014 | 0.051 | 99 | 94 | 10 | 0.281 | -0.014 | 0.023 | 0.556 |
| rs7847 | *CHST11* | 12:103679735 | am_10579 | 122 | 118 | 15 | 0.290 | 0.028 | 0.014 | 0.048 | 100 | 92 | 10 | 0.277 | -0.012 | 0.024 | 0.600 |
| rs886205 | *ALDH2* | 12:110688810 | am_10581 | 182 | 67 | 6 | 0.155 | 0.016 | 0.017 | 0.346 | 137 | 56 | 10 | 0.187 | -0.004 | 0.025 | 0.882 |
| rs4149117 | *SLCO1B3* | 12:20902747 | am_10481 | 207 | 47 | 1 | 0.096 | 0.002 | 0.023 | 0.939 | 137 | 58 | 8 | 0.182 | 0.006 | 0.024 | 0.803 |
| rs7311358 | *SLCO1B3* | 12:20907027 | am_10482 | 207 | 47 | 1 | 0.096 | 0.002 | 0.023 | 0.939 | 137 | 58 | 8 | 0.182 | 0.006 | 0.024 | 0.803 |
| rs2053098 | *SLCO1B3* | 12:20927678 | am_10487 | 207 | 47 | 1 | 0.096 | 0.002 | 0.023 | 0.939 | 137 | 58 | 8 | 0.182 | 0.006 | 0.024 | 0.803 |
| rs2306283 | *SLCO1B1* | 12:21221005 | am_10496 | 94 | 110 | 51 | 0.416 | 0.013 | 0.013 | 0.319 | 70 | 102 | 31 | 0.404 | 0.049 | 0.020 | 0.013 |
| rs11045819 | *SLCO1B1* | 12:21221080 | am_10498 | 192 | 56 | 7 | 0.137 | 0.047 | 0.018 | 0.007 | 153 | 48 | 2 | 0.128 | 0.024 | 0.028 | 0.394 |
| rs4149056 | *SLCO1B1* | 12:21222816 | am_10500 | 178 | 61 | 15 | 0.179 | 0.005 | 0.016 | 0.769 | 148 | 48 | 6 | 0.149 | -0.011 | 0.029 | 0.703 |
| rs4149057 | *SLCO1B1* | 12:21222866 | am_10501 | 104 | 102 | 49 | 0.392 | -0.014 | 0.012 | 0.263 | 73 | 99 | 31 | 0.397 | 0.022 | 0.020 | 0.275 |
| rs2291075 | *SLCO1B1* | 12:21222892 | am_10503 | 87 | 123 | 45 | 0.418 | 0.021 | 0.014 | 0.121 | 82 | 92 | 29 | 0.369 | 0.036 | 0.020 | 0.073 |
| rs4078 | *SLCO1A2* | 12:21368250 | am_10539 | 184 | 67 | 4 | 0.147 | -0.017 | 0.019 | 0.369 | 145 | 56 | 2 | 0.148 | -0.015 | 0.028 | 0.588 |
| rs7957203 | *SLCO1A2* | 12:21368729 | am_10540 | 116 | 122 | 17 | 0.306 | 0.012 | 0.015 | 0.435 | 88 | 91 | 24 | 0.342 | 0.008 | 0.019 | 0.687 |
| rs3803258 | *SLC10A2* | 13:102494988 | am_10666 | 179 | 63 | 13 | 0.175 | 0.015 | 0.017 | 0.374 | 126 | 69 | 8 | 0.209 | 0.036 | 0.023 | 0.121 |
| rs2301159 | *SLC10A2* | 13:102495729 | am_10671 | 132 | 109 | 14 | 0.269 | -0.015 | 0.015 | 0.318 | 111 | 79 | 13 | 0.259 | -0.002 | 0.022 | 0.915 |
| rs279942 | *SLC10A2* | 13:102496111 | am_10672 | 147 | 96 | 12 | 0.235 | -0.016 | 0.015 | 0.292 | 116 | 76 | 11 | 0.241 | -0.009 | 0.022 | 0.676 |
| rs279941 | *SLC10A2* | 13:102496169 | am_10673 | 209 | 43 | 3 | 0.096 | -0.011 | 0.021 | 0.600 | 155 | 46 | 2 | 0.123 | 0.019 | 0.032 | 0.561 |
| rs2301157 | *SLC10A2* | 13:102496364 | am_10675 | 66 | 133 | 56 | 0.480 | -0.016 | 0.013 | 0.219 | 60 | 100 | 43 | 0.458 | -0.004 | 0.019 | 0.818 |
| rs188096 | *SLC10A2* | 13:102503045 | am_10678 | 209 | 43 | 3 | 0.096 | -0.011 | 0.021 | 0.600 | 155 | 46 | 2 | 0.123 | 0.019 | 0.032 | 0.561 |
| rs7987433 | *SLC10A2* | 13:102517057 | am_10686 | 172 | 78 | 4 | 0.169 | -0.004 | 0.018 | 0.803 | 153 | 49 | 1 | 0.126 | -0.037 | 0.029 | 0.191 |
| rs1051332 | *ATP7B* | 13:51405721 | am_10588 | 56 | 128 | 71 | 0.529 | -0.004 | 0.013 | 0.784 | 57 | 113 | 33 | 0.441 | -0.034 | 0.020 | 0.096 |
| rs1801249 | *ATP7B* | 13:51413355 | am_10590 | 113 | 109 | 33 | 0.343 | 0.003 | 0.013 | 0.843 | 70 | 104 | 29 | 0.399 | 0.026 | 0.020 | 0.179 |
| rs732774 | *ATP7B* | 13:51421809 | am_10592 | 113 | 109 | 33 | 0.343 | 0.003 | 0.013 | 0.843 | 70 | 104 | 29 | 0.399 | 0.026 | 0.020 | 0.179 |
| rs1061472 | *ATP7B* | 13:51422489 | am_10593 | 108 | 109 | 37 | 0.360 | 0.005 | 0.013 | 0.680 | 69 | 103 | 31 | 0.406 | 0.037 | 0.020 | 0.059 |
| rs1801244 | *ATP7B* | 13:51442806 | am_10595 | 52 | 127 | 76 | 0.547 | 0.003 | 0.013 | 0.819 | 53 | 112 | 38 | 0.463 | -0.036 | 0.020 | 0.068 |
| rs1801243 | *ATP7B* | 13:51446141 | am_10596 | 77 | 127 | 51 | 0.449 | 0.002 | 0.013 | 0.898 | 38 | 114 | 51 | 0.532 | 0.036 | 0.020 | 0.070 |
| rs2277448 | *ATP7B* | 13:51483549 | am_10597 | 147 | 84 | 24 | 0.259 | 0.008 | 0.014 | 0.570 | 106 | 83 | 14 | 0.273 | 0.039 | 0.021 | 0.062 |
| rs1059751 | *ABCC4* | 13:94470951 | am_10601 | 87 | 131 | 37 | 0.402 | -0.002 | 0.014 | 0.897 | 65 | 98 | 40 | 0.438 | -0.005 | 0.019 | 0.808 |
| rs4148553 | *ABCC4* | 13:94471136 | am_10602 | 87 | 131 | 37 | 0.402 | -0.002 | 0.014 | 0.897 | 65 | 98 | 40 | 0.438 | -0.005 | 0.019 | 0.808 |
| rs4148551 | *ABCC4* | 13:94471519 | am_10603 | 80 | 128 | 47 | 0.435 | -0.001 | 0.013 | 0.911 | 74 | 86 | 43 | 0.424 | -0.016 | 0.020 | 0.404 |
| rs3742106 | *ABCC4* | 13:94471792 | am_10604 | 80 | 128 | 47 | 0.435 | -0.001 | 0.013 | 0.911 | 74 | 86 | 43 | 0.424 | -0.016 | 0.020 | 0.404 |
| rs1751034 | *ABCC4* | 13:94512977 | am_10611 | 165 | 79 | 11 | 0.198 | 0.025 | 0.017 | 0.141 | 140 | 52 | 11 | 0.182 | 0.031 | 0.026 | 0.224 |
| rs2274405 | *ABCC4* | 13:94656979 | am_10632 | 119 | 104 | 32 | 0.329 | -0.038 | 0.013 | 0.002 | 89 | 97 | 17 | 0.323 | 0.008 | 0.021 | 0.717 |
| rs2274406 | *ABCC4* | 13:94656997 | am_10633 | 115 | 108 | 32 | 0.337 | -0.034 | 0.013 | 0.007 | 89 | 97 | 17 | 0.323 | 0.008 | 0.021 | 0.717 |
| rs1339067 | *SLC15A1* | 13:98154613 | am_10650 | 99 | 108 | 48 | 0.400 | 0.026 | 0.012 | 0.027 | 75 | 101 | 25 | 0.376 | -0.041 | 0.021 | 0.052 |
| rs2297322 | *SLC15A1* | 13:98174182 | am_10659 | 197 | 54 | 4 | 0.122 | -0.022 | 0.020 | 0.275 | 162 | 40 | 1 | 0.103 | -0.079 | 0.031 | 0.012 |
| rs1061040 | *SLC7A7* | 14:22312668 | am_10687 | 212 | 41 | 2 | 0.088 | -0.008 | 0.022 | 0.700 | 164 | 36 | 3 | 0.103 | -0.044 | 0.033 | 0.184 |
| rs1805061 | *SLC7A7* | 14:22317952 | am_10691 | 195 | 56 | 4 | 0.125 | -0.013 | 0.019 | 0.496 | 161 | 41 | 1 | 0.106 | -0.006 | 0.037 | 0.866 |
| rs8018462 | *SLC7A7* | 14:22351950 | am_10692 | 61 | 151 | 43 | 0.465 | 0.022 | 0.014 | 0.113 | 64 | 99 | 39 | 0.438 | 0.023 | 0.020 | 0.247 |
| rs1805059 | *SLC7A7* | 14:22352289 | am_10696 | 89 | 141 | 25 | 0.375 | 0.023 | 0.014 | 0.120 | 83 | 97 | 23 | 0.352 | 0.029 | 0.021 | 0.163 |
| rs2281677 | *SLC7A7* | 14:22354412 | am_10697 | 79 | 147 | 29 | 0.402 | 0.019 | 0.015 | 0.187 | 80 | 97 | 26 | 0.367 | 0.030 | 0.021 | 0.140 |
| rs2236135 | *SLC7A8* | 14:22665561 | am_10698 | 204 | 50 | 1 | 0.102 | -0.004 | 0.021 | 0.847 | 144 | 54 | 5 | 0.158 | 0.004 | 0.028 | 0.878 |
| rs2268877 | *SLC7A8* | 14:22706597 | am_10710 | 156 | 94 | 5 | 0.204 | -0.007 | 0.017 | 0.671 | 127 | 70 | 6 | 0.202 | -0.007 | 0.025 | 0.778 |
| rs2268873 | *SLC7A8* | 14:22710634 | am_10711 | 148 | 98 | 9 | 0.227 | -0.006 | 0.016 | 0.722 | 130 | 66 | 7 | 0.197 | -0.008 | 0.025 | 0.740 |
| rs8013529 | *SLC7A8* | 14:22719632 | am_10712 | 190 | 57 | 8 | 0.143 | 0.005 | 0.018 | 0.789 | 163 | 37 | 2 | 0.101 | -0.016 | 0.037 | 0.658 |
| rs1884545 | *SLC7A8* | 14:22721844 | am_10713 | 188 | 62 | 5 | 0.141 | 0.014 | 0.018 | 0.432 | 162 | 39 | 2 | 0.106 | -0.008 | 0.035 | 0.818 |
| rs7141505 | *SLC7A8* | 14:22723028 | am_10714 | 116 | 114 | 25 | 0.322 | 0.017 | 0.014 | 0.243 | 110 | 82 | 11 | 0.256 | -0.048 | 0.022 | 0.027 |
| rs3177427 | *GSTZ1* | 14:76862960 | am_10724 | 111 | 122 | 22 | 0.325 | -0.012 | 0.015 | 0.440 | 95 | 84 | 24 | 0.325 | -0.023 | 0.021 | 0.295 |
| rs1046428 | *GSTZ1* | 14:76864036 | am_10726 | 168 | 81 | 6 | 0.182 | 0.000 | 0.018 | 0.991 | 117 | 74 | 12 | 0.241 | 0.041 | 0.022 | 0.066 |
| rs1060896 | *SLC28A2* | 15:43341559 | am_10730 | 111 | 113 | 31 | 0.343 | 0.003 | 0.014 | 0.805 | 89 | 88 | 26 | 0.345 | 0.030 | 0.020 | 0.142 |
| rs4646 | *CYP19A1* | 15:49290136 | am_10742 | 134 | 102 | 19 | 0.275 | -0.001 | 0.015 | 0.965 | 112 | 75 | 16 | 0.264 | -0.021 | 0.022 | 0.352 |
| rs10046 | *CYP19A1* | 15:49290278 | am_10744 | 72 | 124 | 59 | 0.475 | 0.008 | 0.013 | 0.543 | 63 | 107 | 33 | 0.426 | 0.026 | 0.020 | 0.204 |
| rs700518 | *CYP19A1* | 15:49316404 | am_10748 | 68 | 120 | 66 | 0.496 | 0.001 | 0.013 | 0.927 | 61 | 107 | 35 | 0.436 | 0.023 | 0.021 | 0.260 |
| rs1062033 | *CYP19A1* | 15:49335230 | am_10750 | 79 | 129 | 47 | 0.437 | 0.004 | 0.013 | 0.764 | 39 | 118 | 46 | 0.517 | -0.023 | 0.021 | 0.270 |
| rs762551 | *CYP1A2* | 15:72828970 | am_10785 | 134 | 98 | 23 | 0.282 | -0.001 | 0.014 | 0.961 | 98 | 86 | 19 | 0.305 | 0.017 | 0.022 | 0.451 |
| rs2470890 | *CYP1A2* | 15:72834479 | am_10807 | 109 | 116 | 30 | 0.345 | -0.007 | 0.013 | 0.596 | 82 | 98 | 23 | 0.355 | 0.006 | 0.021 | 0.780 |
| rs2290272 | *SLC28A1* | 15:83248435 | am_10816 | 103 | 117 | 35 | 0.367 | -0.007 | 0.013 | 0.619 | 91 | 88 | 24 | 0.335 | 0.003 | 0.020 | 0.895 |
| rs8187758 | *SLC28A1* | 15:83249879 | am_10820 | 156 | 85 | 14 | 0.222 | -0.001 | 0.016 | 0.971 | 129 | 62 | 12 | 0.212 | 0.012 | 0.024 | 0.610 |
| rs2305367 | *SLC28A1* | 15:83277445 | am_10825 | 118 | 100 | 37 | 0.341 | -0.005 | 0.013 | 0.683 | 82 | 105 | 16 | 0.337 | 0.025 | 0.022 | 0.255 |
| rs2242048 | *SLC28A1* | 15:83279414 | am_10829 | 204 | 48 | 3 | 0.106 | 0.044 | 0.020 | 0.031 | 158 | 42 | 3 | 0.118 | -0.047 | 0.032 | 0.134 |
| rs2242046 | *SLC28A1* | 15:83279733 | am_10832 | 58 | 116 | 81 | 0.545 | -0.012 | 0.012 | 0.324 | 42 | 104 | 57 | 0.537 | -0.002 | 0.020 | 0.939 |
| rs8025045 | *SLC28A1* | 15:83289576 | am_10839 | 201 | 51 | 3 | 0.112 | 0.041 | 0.020 | 0.038 | 158 | 42 | 3 | 0.118 | -0.047 | 0.032 | 0.134 |
| rs1517618 | *SLCO3A1* | 15:90448649 | am_10890 | 172 | 72 | 11 | 0.184 | -0.001 | 0.016 | 0.951 | 135 | 65 | 3 | 0.175 | 0.005 | 0.029 | 0.863 |
| rs2190748 | *SLCO3A1* | 15:90486082 | am_10899 | 86 | 122 | 47 | 0.424 | -0.007 | 0.013 | 0.589 | 64 | 98 | 41 | 0.443 | 0.005 | 0.021 | 0.809 |
| rs2283458 | *SLCO3A1* | 15:90490116 | am_10900 | 109 | 113 | 33 | 0.351 | 0.003 | 0.013 | 0.812 | 104 | 81 | 18 | 0.288 | 0.034 | 0.022 | 0.123 |
| rs960440 | *SLCO3A1* | 15:90499647 | am_10902 | 156 | 91 | 8 | 0.210 | 0.013 | 0.016 | 0.435 | 140 | 56 | 7 | 0.172 | 0.034 | 0.028 | 0.224 |
| rs3743369 | *SLCO3A1* | 15:90508573 | am_10903 | 86 | 129 | 40 | 0.410 | 0.005 | 0.014 | 0.722 | 74 | 105 | 24 | 0.377 | 0.050 | 0.023 | 0.027 |
| rs909921 | *TPSG1* | 16:1213804 | am_10913 | 83 | 126 | 46 | 0.427 | 0.001 | 0.013 | 0.911 | 62 | 101 | 40 | 0.446 | 0.031 | 0.019 | 0.103 |
| rs246221 | *ABCC1* | 16:16045823 | am_10920 | 112 | 113 | 30 | 0.339 | 0.001 | 0.013 | 0.941 | 105 | 80 | 18 | 0.286 | 0.009 | 0.020 | 0.636 |
| rs8187858 | *ABCC1* | 16:16069540 | am_10928 | 205 | 46 | 4 | 0.106 | -0.020 | 0.021 | 0.345 | 164 | 37 | 2 | 0.101 | -0.001 | 0.036 | 0.970 |
| rs212090 | *ABCC1* | 16:16143505 | am_10944 | 87 | 120 | 48 | 0.424 | -0.008 | 0.013 | 0.550 | 55 | 97 | 50 | 0.488 | 0.035 | 0.019 | 0.068 |
| rs4148380 | *ABCC1* | 16:16143932 | am_10945 | 229 | 24 | 2 | 0.055 | 0.039 | 0.027 | 0.154 | 178 | 22 | 2 | 0.064 | 0.038 | 0.040 | 0.335 |
| rs212091 | *ABCC1* | 16:16144151 | am_10947 | 193 | 51 | 11 | 0.143 | 0.011 | 0.017 | 0.527 | 142 | 49 | 12 | 0.180 | -0.015 | 0.024 | 0.535 |
| rs2238472 | *ABCC6* | 16:16159100 | am_10959 | 141 | 93 | 21 | 0.265 | -0.023 | 0.014 | 0.096 | 110 | 83 | 10 | 0.254 | 0.040 | 0.022 | 0.073 |
| rs2856585 | *ABCC6* | 16:16171164 | am_10964 | 215 | 38 | 2 | 0.082 | 0.002 | 0.024 | 0.936 | 182 | 19 | 1 | 0.052 | -0.023 | 0.043 | 0.585 |
| rs8058694 | *ABCC6* | 16:16186364 | am_10977 | 85 | 111 | 59 | 0.449 | -0.016 | 0.012 | 0.191 | 51 | 103 | 49 | 0.495 | 0.018 | 0.020 | 0.368 |
| rs8058696 | *ABCC6* | 16:16186370 | am_10978 | 85 | 111 | 59 | 0.449 | -0.016 | 0.012 | 0.191 | 51 | 103 | 49 | 0.495 | 0.018 | 0.020 | 0.368 |
| rs11401 | *SULT1A2* | 16:28510492 | am_10985 | 180 | 71 | 4 | 0.155 | -0.001 | 0.019 | 0.944 | 128 | 68 | 4 | 0.190 | 0.020 | 0.024 | 0.416 |
| rs9282861 | *SULT1A1* | 16:28525015 | am_11005 | 124 | 103 | 27 | 0.309 | 0.007 | 0.014 | 0.595 | 109 | 83 | 11 | 0.259 | 0.000 | 0.022 | 0.989 |
| rs11859842 | *SPN* | 16:29568718 | am_11022 | 66 | 142 | 47 | 0.463 | 0.007 | 0.014 | 0.590 | 59 | 103 | 41 | 0.456 | 0.006 | 0.018 | 0.742 |
| rs11150564 | *SPN* | 16:29574237 | am_11023 | 71 | 145 | 38 | 0.435 | 0.009 | 0.015 | 0.549 | 61 | 103 | 39 | 0.446 | 0.035 | 0.018 | 0.052 |
| rs13331798 | *QPRT* | 16:29654792 | am_11026 | 177 | 68 | 9 | 0.169 | -0.005 | 0.016 | 0.755 | 122 | 69 | 12 | 0.229 | 0.009 | 0.023 | 0.704 |
| rs7294 | *VKORC1* | 16:31009822 | am_11034 | 103 | 122 | 30 | 0.357 | 0.006 | 0.013 | 0.668 | 67 | 102 | 34 | 0.419 | -0.010 | 0.019 | 0.593 |
| rs2359612 | *VKORC1* | 16:31011297 | am_11040 | 76 | 131 | 47 | 0.443 | -0.004 | 0.014 | 0.744 | 84 | 95 | 23 | 0.349 | 0.008 | 0.020 | 0.701 |
| rs8050894 | *VKORC1* | 16:31012010 | am_11043 | 74 | 134 | 47 | 0.447 | -0.010 | 0.014 | 0.488 | 84 | 95 | 24 | 0.352 | 0.014 | 0.020 | 0.472 |
| rs9934438 | *VKORC1* | 16:31012379 | am_11045 | 76 | 132 | 47 | 0.443 | -0.004 | 0.014 | 0.744 | 86 | 94 | 23 | 0.345 | 0.016 | 0.020 | 0.438 |
| rs17708472 | *VKORC1* | 16:31012854 | am_11047 | 162 | 90 | 3 | 0.188 | 0.002 | 0.017 | 0.901 | 119 | 75 | 9 | 0.229 | -0.005 | 0.024 | 0.831 |
| rs2884737 | *VKORC1* | 16:31013055 | am_11049 | 118 | 103 | 34 | 0.335 | -0.006 | 0.014 | 0.670 | 114 | 77 | 12 | 0.249 | 0.026 | 0.021 | 0.219 |
| rs9923231 | *VKORC1* | 16:31015190 | am_11054 | 75 | 132 | 47 | 0.445 | -0.005 | 0.014 | 0.712 | 85 | 94 | 23 | 0.347 | 0.016 | 0.020 | 0.437 |
| rs4783745 | *CES2* | 16:65528476 | am_11066 | 182 | 63 | 10 | 0.163 | 0.016 | 0.017 | 0.354 | 139 | 58 | 5 | 0.168 | -0.017 | 0.027 | 0.525 |
| rs10517 | *NQO1* | 16:68301261 | am_11078 | 196 | 55 | 4 | 0.124 | -0.024 | 0.020 | 0.241 | 160 | 34 | 8 | 0.124 | -0.003 | 0.029 | 0.932 |
| rs1800566 | *NQO1* | 16:68302646 | am_11084 | 176 | 73 | 6 | 0.167 | -0.012 | 0.018 | 0.519 | 125 | 72 | 6 | 0.207 | -0.021 | 0.026 | 0.430 |
| rs3784932 | *CHST5* | 16:74121788 | am_11128 | 157 | 86 | 11 | 0.213 | -0.013 | 0.015 | 0.404 | 145 | 46 | 10 | 0.164 | 0.034 | 0.025 | 0.169 |
| rs2738792 | *CHST5* | 16:74122785 | am_11130 | 215 | 38 | 2 | 0.082 | 0.045 | 0.022 | 0.044 | 172 | 30 | 1 | 0.079 | -0.047 | 0.035 | 0.182 |
| rs2641806 | *CHST5* | 16:74122921 | am_11131 | 132 | 97 | 26 | 0.292 | 0.007 | 0.013 | 0.622 | 125 | 60 | 18 | 0.236 | 0.001 | 0.022 | 0.971 |
| rs1060253 | *SLC7A5* | 16:86423639 | am_11135 | 145 | 99 | 11 | 0.237 | 0.005 | 0.016 | 0.775 | 103 | 76 | 24 | 0.305 | 0.002 | 0.020 | 0.902 |
| rs2292954 | *SPG7* | 16:88140624 | am_11147 | 172 | 75 | 8 | 0.178 | 0.034 | 0.017 | 0.043 | 130 | 66 | 7 | 0.197 | 0.036 | 0.024 | 0.127 |
| rs12960 | *SPG7* | 16:88147829 | am_11156 | 172 | 75 | 8 | 0.178 | 0.034 | 0.017 | 0.043 | 131 | 65 | 7 | 0.195 | 0.040 | 0.024 | 0.096 |
| rs9930567 | *RPL13* | 16:88155574 | am_11168 | 157 | 85 | 13 | 0.218 | -0.004 | 0.015 | 0.820 | 122 | 71 | 10 | 0.224 | -0.010 | 0.024 | 0.666 |
| rs2072330 | *ALDH3A1* | 17:19585064 | am_11203 | 108 | 97 | 45 | 0.374 | 0.008 | 0.012 | 0.515 | 79 | 83 | 27 | 0.362 | -0.028 | 0.023 | 0.211 |
| rs887241 | *ALDH3A1* | 17:19586530 | am_11205 | 113 | 113 | 29 | 0.335 | 0.012 | 0.014 | 0.395 | 91 | 96 | 16 | 0.315 | -0.012 | 0.024 | 0.608 |
| rs2952151 | *PNMT* | 17:35082022 | am_11219 | 122 | 96 | 37 | 0.333 | -0.005 | 0.013 | 0.685 | 73 | 99 | 31 | 0.397 | -0.029 | 0.021 | 0.169 |
| rs2277624 | *ABCC3* | 17:46116104 | am_11236 | 161 | 74 | 20 | 0.224 | 0.019 | 0.014 | 0.169 | 124 | 70 | 9 | 0.217 | 0.006 | 0.024 | 0.815 |
| rs1051640 | *ABCC3* | 17:46123485 | am_11243 | 171 | 67 | 17 | 0.198 | 0.010 | 0.015 | 0.528 | 139 | 58 | 6 | 0.172 | -0.006 | 0.027 | 0.827 |
| rs10898 | *RALBP1* | 18:9526249 | am_11253 | 138 | 80 | 33 | 0.291 | -0.005 | 0.012 | 0.665 | 122 | 59 | 20 | 0.246 | 0.019 | 0.020 | 0.342 |
| rs3322 | *RALBP1* | 18:9526691 | am_11254 | 205 | 46 | 4 | 0.106 | 0.035 | 0.022 | 0.106 | 146 | 55 | 2 | 0.145 | -0.010 | 0.028 | 0.728 |
| rs12680 | *RALBP1* | 18:9527835 | am_11255 | 231 | 21 | 1 | 0.045 | 0.043 | 0.028 | 0.128 | 176 | 25 | 2 | 0.071 | -0.011 | 0.035 | 0.745 |
| rs4808326 | *CYP4F8* | 19:15587147 | am_11263 | 205 | 47 | 3 | 0.104 | 0.007 | 0.020 | 0.744 | 172 | 29 | 2 | 0.081 | -0.044 | 0.035 | 0.201 |
| rs4646523 | *CYP4F8* | 19:15587487 | am_11264 | 110 | 105 | 40 | 0.363 | -0.008 | 0.013 | 0.510 | 105 | 80 | 18 | 0.286 | -0.048 | 0.021 | 0.021 |
| rs2056822 | *CYP4F8* | 19:15600597 | am_11268 | 131 | 105 | 19 | 0.280 | 0.008 | 0.015 | 0.590 | 98 | 91 | 14 | 0.293 | -0.043 | 0.020 | 0.031 |
| rs4239614 | *CYP4F8* | 19:15601220 | am_11269 | 131 | 105 | 19 | 0.280 | 0.008 | 0.015 | 0.590 | 98 | 91 | 14 | 0.293 | -0.043 | 0.020 | 0.031 |
| rs1805041 | *CYP4F3* | 19:15624691 | am_11279 | 123 | 101 | 17 | 0.280 | 0.007 | 0.015 | 0.644 | 113 | 66 | 11 | 0.232 | 0.074 | 0.021 | 0.000 |
| rs4646904 | *CYP4F3* | 19:15624721 | am_11280 | 122 | 81 | 48 | 0.353 | -0.015 | 0.012 | 0.229 | 83 | 93 | 26 | 0.359 | -0.041 | 0.018 | 0.023 |
| rs688755 | *CYP4F12* | 19:15668305 | am_11291 | 150 | 85 | 20 | 0.245 | 0.011 | 0.014 | 0.435 | 119 | 72 | 12 | 0.236 | -0.004 | 0.022 | 0.840 |
| rs593421 | *CYP4F12* | 19:15668830 | am_11296 | 148 | 87 | 20 | 0.249 | 0.010 | 0.014 | 0.461 | 119 | 72 | 12 | 0.236 | -0.004 | 0.022 | 0.840 |
| rs2108622 | *CYP4F2* | 19:15851431 | am_11299 | 140 | 94 | 21 | 0.267 | 0.002 | 0.014 | 0.879 | 100 | 92 | 11 | 0.281 | -0.006 | 0.022 | 0.796 |
| rs2074900 | *CYP4F2* | 19:15857820 | am_11300 | 132 | 106 | 16 | 0.272 | -0.015 | 0.015 | 0.312 | 90 | 90 | 21 | 0.328 | -0.008 | 0.020 | 0.675 |
| rs3093106 | *CYP4F2* | 19:15869257 | am_11307 | 189 | 61 | 5 | 0.139 | -0.001 | 0.018 | 0.945 | 140 | 59 | 4 | 0.165 | 0.000 | 0.024 | 0.988 |
| rs3093105 | *CYP4F2* | 19:15869388 | am_11310 | 179 | 61 | 6 | 0.148 | 0.005 | 0.017 | 0.752 | 135 | 54 | 1 | 0.147 | 0.010 | 0.021 | 0.647 |
| rs1060463 | *CYP4F11* | 19:15886176 | am_11313 | 90 | 124 | 41 | 0.404 | 0.008 | 0.013 | 0.512 | 87 | 92 | 24 | 0.345 | 0.009 | 0.019 | 0.648 |
| rs8104361 | *CYP4F11* | 19:15895714 | am_11314 | 155 | 83 | 17 | 0.229 | 0.027 | 0.014 | 0.057 | 139 | 59 | 5 | 0.170 | 0.010 | 0.026 | 0.690 |
| rs3765070 | *CYP4F11* | 19:15901292 | am_11317 | 90 | 122 | 43 | 0.408 | 0.007 | 0.013 | 0.582 | 87 | 93 | 23 | 0.342 | 0.011 | 0.019 | 0.584 |
| rs2305801 | *CYP4F11* | 19:15906141 | am_11318 | 141 | 97 | 17 | 0.257 | 0.024 | 0.014 | 0.084 | 136 | 62 | 5 | 0.177 | 0.005 | 0.025 | 0.844 |
| rs1064349 | *CHST8* | 19:38956194 | am_11325 | 191 | 59 | 5 | 0.135 | -0.034 | 0.019 | 0.071 | 159 | 43 | 1 | 0.111 | -0.049 | 0.029 | 0.091 |
| rs8192729 | *CYP2A6* | 19:46042836 | am_11334 | 226 | 26 | 1 | 0.055 | -0.001 | 0.026 | 0.958 | 176 | 25 | 1 | 0.067 | -0.024 | 0.043 | 0.582 |
| rs4079369 | *CYP2A6* | 19:46044602 | am_11342 | 227 | 26 | 1 | 0.055 | -0.001 | 0.027 | 0.964 | 176 | 25 | 1 | 0.067 | -0.024 | 0.043 | 0.575 |
| rs1137115 | *CYP2A6* | 19:46048121 | am_11358 | 123 | 118 | 14 | 0.286 | -0.053 | 0.015 | 0.000 | 125 | 69 | 9 | 0.214 | -0.068 | 0.023 | 0.003 |
| rs4803381 | *CYP2A6* | 19:46049184 | am_11364 | 90 | 138 | 23 | 0.367 | -0.054 | 0.014 | 0.000 | 95 | 86 | 20 | 0.313 | -0.070 | 0.020 | 0.000 |
| rs3869579 | *CYP2A7* | 19:46075639 | am_11376 | 66 | 131 | 57 | 0.482 | 0.009 | 0.013 | 0.479 | 52 | 105 | 46 | 0.485 | -0.005 | 0.020 | 0.799 |
| rs4803418 | *CYP2B6* | 19:46203643 | am_11407 | 121 | 114 | 20 | 0.302 | -0.036 | 0.014 | 0.010 | 92 | 89 | 21 | 0.324 | -0.003 | 0.021 | 0.868 |
| rs4803419 | *CYP2B6* | 19:46204632 | am_11408 | 117 | 117 | 20 | 0.309 | -0.037 | 0.014 | 0.008 | 91 | 91 | 20 | 0.324 | 0.000 | 0.021 | 0.998 |
| rs3745274 | *CYP2B6* | 19:46204681 | am_11411 | 149 | 94 | 9 | 0.222 | 0.031 | 0.016 | 0.046 | 108 | 77 | 17 | 0.275 | 0.005 | 0.023 | 0.832 |
| rs2279343 | *CYP2B6* | 19:46207103 | am_11415 | 139 | 99 | 14 | 0.252 | 0.037 | 0.015 | 0.012 | 101 | 78 | 23 | 0.307 | -0.010 | 0.022 | 0.640 |
| rs2279344 | *CYP2B6* | 19:46207323 | am_11416 | 78 | 141 | 36 | 0.418 | 0.001 | 0.014 | 0.932 | 91 | 81 | 31 | 0.352 | -0.009 | 0.020 | 0.628 |
| rs8192719 | *CYP2B6* | 19:46210613 | am_11423 | 150 | 91 | 14 | 0.233 | 0.033 | 0.015 | 0.027 | 109 | 74 | 20 | 0.281 | 0.000 | 0.023 | 0.997 |
| rs1709082 | *CYP2A13* | 19:46293449 | am_11449 | 211 | 43 | 1 | 0.088 | 0.023 | 0.022 | 0.313 | 155 | 47 | 1 | 0.121 | -0.030 | 0.036 | 0.401 |
| rs305968 | *CYP2F1* | 19:46314029 | am_11456 | 133 | 109 | 13 | 0.265 | 0.004 | 0.015 | 0.772 | 89 | 98 | 16 | 0.320 | -0.021 | 0.022 | 0.333 |
| rs296365 | *SULT2A1* | 19:53066363 | am_11474 | 139 | 90 | 25 | 0.276 | -0.009 | 0.014 | 0.490 | 106 | 80 | 17 | 0.281 | 0.014 | 0.023 | 0.528 |
| rs2544794 | *SULT2B1* | 19:53771058 | am_11482 | 192 | 60 | 2 | 0.126 | -0.020 | 0.020 | 0.319 | 147 | 53 | 3 | 0.145 | -0.004 | 0.030 | 0.890 |
| rs2302948 | *SULT2B1* | 19:53787877 | am_11487 | 161 | 80 | 14 | 0.212 | -0.010 | 0.015 | 0.496 | 103 | 86 | 14 | 0.281 | -0.042 | 0.022 | 0.050 |
| rs1132054 | *SULT2B1* | 19:53794211 | am_11490 | 76 | 135 | 44 | 0.437 | -0.014 | 0.013 | 0.296 | 39 | 118 | 46 | 0.517 | -0.049 | 0.022 | 0.025 |
| rs5629 | *PTGIS* | 20:47563113 | am_12126 | 144 | 91 | 20 | 0.257 | 0.000 | 0.014 | 0.984 | 111 | 84 | 8 | 0.246 | 0.018 | 0.023 | 0.434 |
| rs4809957 | *CYP24A1* | 20:52204578 | am_12137 | 150 | 90 | 13 | 0.229 | -0.035 | 0.016 | 0.030 | 127 | 64 | 12 | 0.217 | -0.012 | 0.024 | 0.611 |
| rs2762934 | *CYP24A1* | 20:52204668 | am_12140 | 196 | 53 | 6 | 0.127 | 0.010 | 0.019 | 0.588 | 149 | 50 | 4 | 0.143 | -0.007 | 0.026 | 0.795 |
| rs6068816 | *CYP24A1* | 20:52214498 | am_12145 | 195 | 56 | 4 | 0.125 | -0.017 | 0.019 | 0.389 | 174 | 27 | 2 | 0.076 | 0.037 | 0.035 | 0.285 |
| rs2296241 | *CYP24A1* | 20:52219626 | am_12147 | 66 | 133 | 56 | 0.480 | -0.003 | 0.013 | 0.826 | 52 | 108 | 43 | 0.478 | 0.031 | 0.021 | 0.149 |
| rs2236553 | *SLCO4A1* | 20:60760188 | am_12160 | 153 | 85 | 16 | 0.230 | 0.000 | 0.015 | 0.998 | 135 | 60 | 8 | 0.187 | -0.003 | 0.024 | 0.889 |
| rs3787537 | *SLCO4A1* | 20:60774187 | am_12165 | 148 | 96 | 11 | 0.231 | 0.011 | 0.016 | 0.477 | 128 | 68 | 7 | 0.202 | 0.005 | 0.024 | 0.850 |
| rs1005695 | *CBR1* | 21:36365435 | am_12169 | 82 | 124 | 49 | 0.435 | 0.021 | 0.013 | 0.122 | 58 | 104 | 41 | 0.458 | 0.003 | 0.020 | 0.882 |
| rs3787728 | *CBR1* | 21:36365763 | am_12170 | 119 | 108 | 28 | 0.322 | 0.002 | 0.014 | 0.878 | 91 | 90 | 22 | 0.330 | -0.010 | 0.020 | 0.617 |
| rs2835265 | *CBR1* | 21:36366566 | am_12171 | 203 | 47 | 1 | 0.098 | 0.056 | 0.022 | 0.012 | 163 | 38 | 2 | 0.103 | 0.022 | 0.033 | 0.497 |
| rs998383 | *CBR1* | 21:36367609 | am_12172 | 104 | 122 | 29 | 0.353 | 0.011 | 0.014 | 0.457 | 82 | 96 | 25 | 0.360 | 0.004 | 0.021 | 0.848 |
| rs8133052 | *CBR3* | 21:36429371 | am_12174 | 67 | 128 | 55 | 0.476 | -0.002 | 0.013 | 0.872 | 48 | 108 | 46 | 0.495 | -0.009 | 0.019 | 0.646 |
| rs2835286 | *CBR3* | 21:36440232 | am_12178 | 172 | 70 | 13 | 0.188 | -0.011 | 0.016 | 0.479 | 127 | 72 | 4 | 0.197 | -0.020 | 0.028 | 0.488 |
| rs1056892 | *CBR3* | 21:36440576 | am_12181 | 104 | 126 | 25 | 0.345 | 0.005 | 0.014 | 0.745 | 101 | 78 | 24 | 0.310 | 0.023 | 0.020 | 0.264 |
| rs492338 | *ABCG1* | 21:42575046 | am_12204 | 71 | 127 | 57 | 0.473 | 0.004 | 0.013 | 0.726 | 52 | 96 | 55 | 0.507 | 0.008 | 0.018 | 0.655 |
| rs3788007 | *ABCG1* | 21:42579845 | am_12205 | 164 | 81 | 10 | 0.198 | 0.039 | 0.016 | 0.012 | 144 | 56 | 3 | 0.153 | 0.013 | 0.027 | 0.626 |
| rs425215 | *ABCG1* | 21:42580170 | am_12206 | 107 | 108 | 40 | 0.369 | 0.003 | 0.013 | 0.800 | 79 | 89 | 35 | 0.392 | -0.005 | 0.018 | 0.770 |
| rs914189 | *ABCG1* | 21:42583978 | am_12207 | 159 | 88 | 8 | 0.204 | -0.034 | 0.017 | 0.040 | 107 | 81 | 15 | 0.273 | -0.003 | 0.022 | 0.884 |
| rs3788010 | *ABCG1* | 21:42589091 | am_12208 | 95 | 123 | 37 | 0.386 | 0.017 | 0.014 | 0.240 | 73 | 108 | 22 | 0.374 | 0.018 | 0.022 | 0.402 |
| rs1044317 | *ABCG1* | 21:42589970 | am_12209 | 90 | 127 | 38 | 0.398 | 0.016 | 0.014 | 0.260 | 69 | 107 | 27 | 0.397 | 0.028 | 0.021 | 0.195 |
| rs1541290 | *ABCG1* | 21:42591552 | am_12210 | 68 | 145 | 42 | 0.449 | 0.002 | 0.015 | 0.919 | 51 | 109 | 43 | 0.480 | 0.009 | 0.020 | 0.643 |
| rs12659 | *SLC19A1* | 21:45775984 | am_12214 | 83 | 104 | 35 | 0.392 | -0.001 | 0.013 | 0.916 | 65 | 91 | 36 | 0.424 | -0.020 | 0.019 | 0.295 |
| rs1051266 | *SLC19A1* | 21:45782222 | am_12216 | 74 | 113 | 66 | 0.484 | -0.017 | 0.013 | 0.166 | 60 | 94 | 49 | 0.473 | -0.020 | 0.019 | 0.280 |
| rs4633 | *COMT* | 22:18330235 | am_12220 | 55 | 140 | 60 | 0.510 | 0.014 | 0.013 | 0.285 | 59 | 100 | 44 | 0.463 | 0.020 | 0.019 | 0.286 |
| rs4680 | *COMT* | 22:18331271 | am_12229 | 57 | 131 | 63 | 0.512 | 0.013 | 0.013 | 0.299 | 59 | 94 | 46 | 0.467 | 0.004 | 0.014 | 0.754 |
| rs1135840 | *CYP2D6* | 22:40852557 | am_12247 | 89 | 122 | 44 | 0.412 | -0.007 | 0.013 | 0.574 | 80 | 95 | 28 | 0.372 | 0.002 | 0.019 | 0.916 |
| rs28371725 | *CYP2D6* | 22:40853749 | am_12257 | 206 | 44 | 5 | 0.106 | -0.048 | 0.019 | 0.011 | 166 | 36 | 1 | 0.094 | -0.005 | 0.035 | 0.888 |
| rs16947 | *CYP2D6* | 22:40853887 | am_12261 | 113 | 112 | 30 | 0.337 | 0.021 | 0.013 | 0.124 | 88 | 101 | 14 | 0.318 | 0.028 | 0.022 | 0.203 |
| rs3892097 | *CYP2D6* | 22:40854891 | am_12274 | 147 | 101 | 7 | 0.225 | -0.009 | 0.017 | 0.602 | 107 | 79 | 17 | 0.278 | -0.022 | 0.021 | 0.296 |
| rs1058164 | *CYP2D6* | 22:40855076 | am_12277 | 88 | 123 | 43 | 0.411 | -0.009 | 0.013 | 0.503 | 81 | 93 | 29 | 0.372 | 0.005 | 0.019 | 0.793 |
| rs1065852 | *CYP2D6* | 22:40856638 | am_12285 | 138 | 105 | 12 | 0.253 | -0.017 | 0.016 | 0.298 | 96 | 88 | 19 | 0.310 | -0.025 | 0.020 | 0.206 |
| rs1080985 | *CYP2D6* | 22:40858326 | am_12291 | 157 | 81 | 17 | 0.225 | 0.051 | 0.014 | 0.000 | 122 | 71 | 10 | 0.224 | 0.029 | 0.021 | 0.178 |
| rs1080983 | *CYP2D6* | 22:40858512 | am_15502 | 119 | 104 | 30 | 0.324 | 0.017 | 0.013 | 0.200 | 92 | 93 | 14 | 0.304 | 0.030 | 0.021 | 0.159 |
| rs28360521 | *CYP2D6* | 22:40858920 | am_15506 | 138 | 105 | 12 | 0.253 | -0.017 | 0.016 | 0.298 | 96 | 88 | 19 | 0.310 | -0.025 | 0.020 | 0.206 |
| rs138056 | *SULT4A1* | 22:42552006 | am_12303 | 154 | 87 | 14 | 0.225 | 0.015 | 0.016 | 0.350 | 106 | 85 | 12 | 0.268 | 0.020 | 0.022 | 0.360 |
| rs138057 | *SULT4A1* | 22:42552580 | am_12304 | 154 | 87 | 14 | 0.225 | 0.015 | 0.016 | 0.350 | 108 | 83 | 12 | 0.264 | 0.028 | 0.022 | 0.216 |
| rs743616 | *ARSA* | 22:49410905 | am_12316 | 64 | 115 | 76 | 0.524 | 0.013 | 0.013 | 0.299 | 65 | 93 | 45 | 0.451 | 0.016 | 0.020 | 0.413 |
| rs131713 | *ARSA* | 22:49415426 | am_12322 | 121 | 103 | 31 | 0.324 | -0.010 | 0.013 | 0.456 | 80 | 92 | 30 | 0.376 | 0.004 | 0.021 | 0.836 |
| rs1137070 | *MAOA* | 23:43488335 | am_15380 | 138 | 77 | 40 | 0.308 | 0.003 | 0.012 | 0.806 | 128 | 43 | 32 | 0.264 | -0.004 | 0.018 | 0.807 |
| rs1799836 | *MAOB* | 23:43512943 | am_15390 | 103 | 98 | 54 | 0.404 | -0.016 | 0.012 | 0.166 | 82 | 64 | 57 | 0.438 | 0.003 | 0.015 | 0.843 |
| rs6521128 | *CHST7* | 23:46330604 | am_15403 | 175 | 50 | 30 | 0.216 | 0.013 | 0.013 | 0.316 | 132 | 42 | 29 | 0.246 | 0.009 | 0.018 | 0.632 |
| rs11796837 | *CHST7* | 23:46334608 | am_15404 | 206 | 36 | 13 | 0.122 | -0.006 | 0.017 | 0.727 | 164 | 22 | 17 | 0.138 | -0.022 | 0.021 | 0.285 |
| rs735716 | *CHST7* | 23:46342730 | am_15407 | 92 | 76 | 87 | 0.490 | -0.010 | 0.011 | 0.359 | 82 | 53 | 68 | 0.466 | -0.005 | 0.016 | 0.743 |
| rs732316 | *CHST7* | 23:46343567 | am_15408 | 94 | 76 | 85 | 0.482 | -0.010 | 0.011 | 0.340 | 82 | 53 | 68 | 0.466 | -0.005 | 0.016 | 0.743 |
| rs2227291 | *ATP7A* | 23:77155158 | am_15454 | 186 | 52 | 17 | 0.169 | 0.002 | 0.015 | 0.894 | 133 | 43 | 27 | 0.239 | -0.011 | 0.018 | 0.551 |

^a^Reflects meta-PACT results for N=247 and N=202; counts are from N=255 and N=203 individuals. For SNPs with multiple rsIDs based on Affymetrix DMET Plus^™^ annotation, consulted with dbSNP (build 138), and for the following rsIDs: rs41397848|rs7512729, kept rs7512729; rs2842934|rs41332952, kept rs2842934; rs1799853|rs28371674|rs41400645, rs1799853; rs13003|rs9930567, kept rs9930567; rs2279343|rs28399497, kept rs2279343; rs1800716|rs3892097, kept rs3892097. For rs11859842 and rs11150564, associated gene was modified to *SPN*; for rs13331798, associated gene was modified to *QPRT*.
