# Supplementary material for: Drug Metabolizing Enzyme and Transporter Gene Variation, Nicotine Metabolism, Prospective Abstinence, and Cigarette Consumption
Source: PLoS One. 2015 Jul 1;10(7):e0126113. doi: 10.1371/journal.pone.0126113 (PMC4488893; doi:10.1371/journal.pone.0126113)
Supplement: S2 Table — (DOCX) [file pone.0126113.s002.docx]

**Supplementary Table 2.** PKFAM DMET^™^ Plus SNP Meta-analysis Results.

| **rsID** | **Gene** | **Chr:Coor** | **Probe ID** | **Z-score** | ***P*** | ***P*_ACT_** | ***P*_Bonferroni_** |
| --- | --- | --- | --- | --- | --- | --- | --- |
| rs4803381 | *CYP2A6* | 19:46049184 | am_11364 | -5.275 | 1.33E-07 | 4.53E-05 | 6.73E-05 |
| rs1137115 | *CYP2A6* | 19:46048121 | am_11358 | -4.657 | 3.21E-06 | 1.20E-03 | 1.63E-03 |
| rs1080985 | *CYP2D6* | 22:40858326 | am_12291 | 3.768 | 1.65E-04 | N.S. | N.S. |
| rs1884725 | *XDH* | 2:31425290 | am_12370 | 2.768 | 0.0056 | N.S. | N.S. |
| rs11045819 | *SLCO1B1* | 12:21221080 | am_10498 | 2.752 | 0.0059 | N.S. | N.S. |
| rs12960 | *SPG7* | 16:88147829 | am_11156 | 2.617 | 0.0089 | N.S. | N.S. |
| rs3856650 | *CHST13* | 3:127700000 | am_13323 | 2.607 | 0.0091 | N.S. | N.S. |
| rs2292954 | *SPG7* | 16:88140624 | am_11147 | 2.538 | 0.0111 | N.S. | N.S. |
| rs1805041 | *CYP4F3* | 19:15624691 | am_11279 | 2.456 | 0.0140 | N.S. | N.S. |
| rs2835265 | *CBR1* | 21:36366566 | am_12171 | 2.447 | 0.0144 | N.S. | N.S. |
| rs2274405 | *ABCC4* | 13:94656979 | am_10632 | -2.443 | 0.0146 | N.S. | N.S. |
| rs1064349 | *CHST8* | 19:38956194 | am_11325 | -2.435 | 0.0149 | N.S. | N.S. |
| rs3788007 | *ABCG1* | 21:42579845 | am_12205 | 2.428 | 0.0152 | N.S. | N.S. |
| rs3750266 | *SLCO5A1* | 8:70907366 | am_15200 | -2.417 | 0.0156 | N.S. | N.S. |
| rs3822172 | *SULT1E1* | 4:70758429 | am_13632 | 2.356 | 0.0185 | N.S. | N.S. |
| rs28371725 | *CYP2D6* | 22:40853749 | am_12257 | -2.299 | 0.0215 | N.S. | N.S. |
| rs2291075 | *SLCO1B1* | 12:21222892 | am_10503 | 2.289 | 0.0221 | N.S. | N.S. |
| rs2297322 | *SLC15A1* | 13:98174182 | am_10659 | -2.284 | 0.0224 | N.S. | N.S. |
| rs1805042 | *CYP4F3* | 19:15624721 | am_11280 | -2.272 | 0.0231 | N.S. | N.S. |
| rs72552763 | *SLC22A1* | 6:160500000 | am_14368 | 2.238 | 0.0252 | N.S. | N.S. |
| rs2235015 | *ABCB1* | 7:87037500 | am_14624 | 2.228 | 0.0259 | N.S. | N.S. |
| rs4803418 | *CYP2B6* | 19:46203643 | am_11407 | -2.225 | 0.0261 | N.S. | N.S. |
| rs4803419 | *CYP2B6* | 19:46204632 | am_11408 | -2.199 | 0.0279 | N.S. | N.S. |
| rs7905939 | *CYP26A1* | 10:94831097 | am_10033 | 2.190 | 0.0285 | N.S. | N.S. |
| rs2306283 | *SLCO1B1* | 12:21221005 | am_10496 | 2.178 | 0.0294 | N.S. | N.S. |
| rs2274406 | *ABCC4* | 13:94656997 | am_10633 | -2.147 | 0.0318 | N.S. | N.S. |
| rs10002894 | *ADH6* | 4:100400000 | am_13759 | 2.117 | 0.0343 | N.S. | N.S. |
| rs3755740 | *CHST2* | 3:144300000 | am_13337 | -2.099 | 0.0358 | N.S. | N.S. |
| rs3755739 | *CHST2* | 3:144300000 | am_13338 | -2.087 | 0.0369 | N.S. | N.S. |
| rs4809957 | *CYP24A1* | 20:52204578 | am_12137 | -2.083 | 0.0373 | N.S. | N.S. |
| rs1695 | *GSTP1* | 11:67109265 | am_10440 | 2.078 | 0.0377 | N.S. | N.S. |
| rs1805059 | *SLC7A7* | 14:22352289 | am_10696 | 2.073 | 0.0382 | N.S. | N.S. |
| rs7087728 | *MAT1A* | 10:82023450 | am_10020 | -2.070 | 0.0385 | N.S. | N.S. |
| rs17102596 | *MAT1A* | 10:82025153 | am_10024 | -2.070 | 0.0385 | N.S. | N.S. |
| rs1132054 | *SULT2B1* | 19:53794211 | am_11490 | -2.051 | 0.0403 | N.S. | N.S. |
| rs2860840 | *CYP2C18* | 10:96485222 | am_10048 | 2.036 | 0.0418 | N.S. | N.S. |
| rs6830685 | *ADH6* | 4:100400000 | am_13761 | 2.005 | 0.0450 | N.S. | N.S. |
| rs12248560 | *CYP2C19* | 10:96511647 | am_10053 | -1.993 | 0.0463 | N.S. | N.S. |
| rs10929303 | *UGT1A1* | 2:234300000 | am_13067 | -1.988 | 0.0468 | N.S. | N.S. |
| rs16947 | *CYP2D6* | 22:40853887 | am_12261 | 1.979 | 0.0478 | N.S. | N.S. |
| rs8018462 | *SLC7A7* | 14:22351950 | am_10692 | 1.960 | 0.0500 | N.S. | N.S. |
| rs10893 | *ABP1* | 7:150200000 | am_14950 | 1.946 | 0.0517 | N.S. | N.S. |
| rs4725373 | *ABP1* | 7:150200000 | am_14953 | 1.946 | 0.0517 | N.S. | N.S. |
| rs11249460 | *SULT1B1* | 4:70640615 | am_13602 | 1.946 | 0.0517 | N.S. | N.S. |
| rs1049793 | *ABP1* | 7:150200000 | am_14954 | 1.933 | 0.0532 | N.S. | N.S. |
| rs2281677 | *SLC7A7* | 14:22354412 | am_10697 | 1.930 | 0.0536 | N.S. | N.S. |
| rs1826909 | *ADH1A* | 4:100400000 | am_13775 | -1.927 | 0.0540 | N.S. | N.S. |
| rs1402467 | *SULT1C2* | 2:108400000 | am_12590 | -1.921 | 0.0547 | N.S. | N.S. |
| rs1751034 | *ABCC4* | 13:94512977 | am_10611 | 1.901 | 0.0573 | N.S. | N.S. |
| rs7668258 | *UGT2B7* | 4:69996667 | am_13459 | -1.889 | 0.0589 | N.S. | N.S. |
| rs7662029 | *UGT2B7* | 4:69996501 | am_13458 | -1.881 | 0.0600 | N.S. | N.S. |
| rs8104361 | *CYP4F11* | 19:15895714 | am_11314 | 1.859 | 0.0630 | N.S. | N.S. |
| rs2235040 | *ABCB1* | 7:87003686 | am_14598 | 1.852 | 0.0640 | N.S. | N.S. |
| rs8192719 | *CYP2B6* | 19:46210613 | am_11423 | 1.847 | 0.0647 | N.S. | N.S. |
| rs7439366 | *UGT2B7* | 4:69998927 | am_13465 | -1.837 | 0.0662 | N.S. | N.S. |
| rs7438284 | *UGT2B7* | 4:69998926 | am_13464 | -1.837 | 0.0662 | N.S. | N.S. |
| rs2072200 | *PON3* | 7:94864096 | am_14704 | 1.829 | 0.0674 | N.S. | N.S. |
| rs12539 | *ABP1* | 7:150200000 | am_14955 | -1.829 | 0.0674 | N.S. | N.S. |
| rs1080983 | *CYP2D6* | 22:40858512 | am_15502 | 1.828 | 0.0675 | N.S. | N.S. |
| rs2279343 | *CYP2B6* | 19:46207103 | am_11415 | 1.813 | 0.0698 | N.S. | N.S. |
| rs1138541 | *SLCO5A1* | 8:70747182 | am_15094 | -1.796 | 0.0725 | N.S. | N.S. |
| rs1042640 | *UGT1A1* | 2:234300000 | am_13068 | -1.776 | 0.0757 | N.S. | N.S. |
| rs3745274 | *CYP2B6* | 19:46204681 | am_11411 | 1.770 | 0.0767 | N.S. | N.S. |
| rs4646523 | *CYP4F8* | 19:15587487 | am_11264 | -1.768 | 0.0771 | N.S. | N.S. |
| rs1051266 | *SLC19A1* | 21:45782222 | am_12216 | -1.752 | 0.0798 | N.S. | N.S. |
| rs6811453 | *ADH1A* | 4:100400000 | am_13763 | -1.745 | 0.0810 | N.S. | N.S. |
| rs818202 | *CDA* | 1:20789378 | am_11500 | 1.728 | 0.0840 | N.S. | N.S. |
| rs914189 | *ABCG1* | 21:42583978 | am_12207 | -1.721 | 0.0853 | N.S. | N.S. |
| rs4148380 | *ABCC1* | 16:16143932 | am_10945 | 1.720 | 0.0854 | N.S. | N.S. |
| rs10882140 | *CYP26A1* | 10:94832203 | am_10035 | 1.714 | 0.0865 | N.S. | N.S. |
| rs11150564 | *SPN* | 16:29574237 | am_11023 | 1.694 | 0.0903 | N.S. | N.S. |
| rs2302948 | *SULT2B1* | 19:53787877 | am_11487 | -1.689 | 0.0912 | N.S. | N.S. |
| rs1902023 | *UGT2B15* | 4:69218679 | am_13439 | -1.678 | 0.0933 | N.S. | N.S. |
| rs7853758 | *SLC28A3* | 9:86090746 | am_15297 | -1.663 | 0.0963 | N.S. | N.S. |
| rs1044317 | *ABCG1* | 21:42589970 | am_12209 | 1.659 | 0.0971 | N.S. | N.S. |
| rs735320 | *CYP8B1* | 3:42890882 | am_13182 | 1.638 | 0.1014 | N.S. | N.S. |
| rs3803258 | *SLC10A2* | 13:102500000 | am_10666 | 1.633 | 0.1025 | N.S. | N.S. |
| rs6664 | *CHST2* | 3:144300000 | am_13346 | -1.627 | 0.1037 | N.S. | N.S. |
| rs1065852 | *CYP2D6* | 22:40856638 | am_12285 | -1.607 | 0.1081 | N.S. | N.S. |
| rs28360521 | *CYP2D6* | 22:40858920 | am_15506 | -1.607 | 0.1081 | N.S. | N.S. |
| rs2305801 | *CYP4F11* | 19:15906141 | am_11318 | 1.606 | 0.1083 | N.S. | N.S. |
| rs2463018 | *CHST11* | 12:103700000 | am_10575 | 1.606 | 0.1083 | N.S. | N.S. |
| rs2180314 | *GSTA2* | 6:52725690 | am_14240 | 1.590 | 0.1118 | N.S. | N.S. |
| rs28365062 | *UGT2B7* | 4:69998860 | am_13463 | -1.558 | 0.1192 | N.S. | N.S. |
| rs16936279 | *SLCO5A1* | 8:70747363 | am_15095 | -1.525 | 0.1273 | N.S. | N.S. |
| rs8330 | *UGT1A1* | 2:234300000 | am_13070 | -1.507 | 0.1318 | N.S. | N.S. |
| rs2028985 | *CHST1* | 11:45651938 | am_10330 | -1.501 | 0.1334 | N.S. | N.S. |
| rs2277448 | *ATP7B* | 13:51483549 | am_10597 | 1.501 | 0.1334 | N.S. | N.S. |
| rs4633 | *COMT* | 22:18330235 | am_12220 | 1.490 | 0.1362 | N.S. | N.S. |
| rs854560 | *PON1* | 7:94784020 | am_14681 | 1.486 | 0.1373 | N.S. | N.S. |
| rs138057 | *SULT4A1* | 22:42552580 | am_12304 | 1.475 | 0.1402 | N.S. | N.S. |
| rs4683739 | *CHST2* | 3:144300000 | am_13339 | -1.474 | 0.1405 | N.S. | N.S. |
| rs2072671 | *CDA* | 1:20788288 | am_11499 | -1.455 | 0.1457 | N.S. | N.S. |
| rs3750268 | *SLCO5A1* | 8:70907725 | am_15202 | -1.446 | 0.1482 | N.S. | N.S. |
| rs3743369 | *SLCO3A1* | 15:90508573 | am_10903 | 1.445 | 0.1485 | N.S. | N.S. |
| rs3788010 | *ABCG1* | 21:42589091 | am_12208 | 1.442 | 0.1493 | N.S. | N.S. |
| rs1789915 | *ADH1C* | 4:100500000 | am_13817 | -1.434 | 0.1516 | N.S. | N.S. |
| rs3756067 | *ALB* | 4:74488509 | am_13647 | 1.431 | 0.1524 | N.S. | N.S. |
| rs903247 | *CHST11* | 12:103700000 | am_10576 | 1.426 | 0.1539 | N.S. | N.S. |
| rs7847 | *CHST11* | 12:103700000 | am_10579 | 1.425 | 0.1542 | N.S. | N.S. |
| rs1061472 | *ATP7B* | 13:51422489 | am_10593 | 1.389 | 0.1648 | N.S. | N.S. |
| rs2020863 | *FMO2* | 1:169400000 | am_11942 | -1.381 | 0.1673 | N.S. | N.S. |
| rs272879 | *SLC22A4* | 5:131700000 | am_13896 | 1.375 | 0.1691 | N.S. | N.S. |
| rs324420 | *FAAH* | 1:46643348 | am_11525 | 1.373 | 0.1698 | N.S. | N.S. |
| rs1005695 | *CBR1* | 21:36365435 | am_12169 | 1.365 | 0.1723 | N.S. | N.S. |
| rs2463437 | *CHST11* | 12:103700000 | am_10577 | 1.363 | 0.1729 | N.S. | N.S. |
| rs272893 | *SLC22A4* | 5:131700000 | am_13894 | 1.356 | 0.1751 | N.S. | N.S. |
| rs4926802 | *CYP4Z1* | 1:47344489 | am_11609 | -1.346 | 0.1783 | N.S. | N.S. |
| rs2267665 | *PPARD* | 6:35477469 | am_14131 | -1.345 | 0.1786 | N.S. | N.S. |
| rs7512785 | *FMO2* | 1:169400000 | am_11968 | -1.343 | 0.1793 | N.S. | N.S. |
| rs7515157 | *FMO2* | 1:169400000 | am_11970 | -1.343 | 0.1793 | N.S. | N.S. |
| rs1530031 | *CHST10* | 2:100400000 | am_12523 | -1.343 | 0.1793 | N.S. | N.S. |
| rs2468110 | *CHST11* | 12:103700000 | am_10573 | 1.322 | 0.1862 | N.S. | N.S. |
| rs592792 | *GSTM4* | 1:110000000 | am_11695 | -1.322 | 0.1862 | N.S. | N.S. |
| rs1881668 | *SULT1E1* | 4:70760045 | am_13635 | 1.321 | 0.1865 | N.S. | N.S. |
| rs743616 | *ARSA* | 22:49410905 | am_12316 | 1.316 | 0.1882 | N.S. | N.S. |
| rs2277624 | *ABCC3* | 17:46116104 | am_11236 | 1.306 | 0.1916 | N.S. | N.S. |
| rs138056 | *SULT4A1* | 22:42552006 | am_12303 | 1.291 | 0.1967 | N.S. | N.S. |
| rs960440 | *SLCO3A1* | 15:90499647 | am_10902 | 1.288 | 0.1977 | N.S. | N.S. |
| rs1003973 | *PPARD* | 6:35485279 | am_14145 | -1.277 | 0.2016 | N.S. | N.S. |
| rs2267668 | *PPARD* | 6:35485900 | am_14146 | -1.266 | 0.2055 | N.S. | N.S. |
| rs2267669 | *PPARD* | 6:35486102 | am_14147 | -1.266 | 0.2055 | N.S. | N.S. |
| rs2038067 | *PPARD* | 6:35482444 | am_14136 | -1.266 | 0.2055 | N.S. | N.S. |
| rs3748930 | *CHST10* | 2:100400000 | am_12532 | -1.266 | 0.2055 | N.S. | N.S. |
| rs4715332 | *GSTA1* | 6:52777144 | am_14286 | 1.263 | 0.2066 | N.S. | N.S. |
| rs4736312 | *CYP11B1* | 8:144000000 | am_15210 | -1.261 | 0.2073 | N.S. | N.S. |
| rs1134095 | *CYP11B1* | 8:144000000 | am_15211 | -1.261 | 0.2073 | N.S. | N.S. |
| rs7003319 | *CYP11B1* | 8:144000000 | am_15212 | -1.261 | 0.2073 | N.S. | N.S. |
| rs5303 | *CYP11B1* | 8:144000000 | am_15217 | -1.261 | 0.2073 | N.S. | N.S. |
| rs2049900 | *AKAP9* | 7:91576724 | am_14662 | 1.253 | 0.2102 | N.S. | N.S. |
| rs274558 | *SLC22A5* | 5:131700000 | am_13934 | 1.250 | 0.2113 | N.S. | N.S. |
| rs3177427 | *GSTZ1* | 14:76862960 | am_10724 | -1.235 | 0.2168 | N.S. | N.S. |
| rs4148304 | *UGT2A1* | 4:70494917 | am_13535 | 1.232 | 0.2179 | N.S. | N.S. |
| rs2020861 | *FMO2* | 1:169400000 | am_11938 | -1.228 | 0.2194 | N.S. | N.S. |
| rs1604741 | *SULT1B1* | 4:70645708 | am_13603 | -1.222 | 0.2217 | N.S. | N.S. |
| rs11584174 | *NR1I3* | 1:159500000 | am_11833 | -1.214 | 0.2247 | N.S. | N.S. |
| rs2297595 | *DPYD* | 1:97937679 | am_11667 | 1.200 | 0.2301 | N.S. | N.S. |
| rs2235013 | *ABCB1* | 7:87016562 | am_14605 | 1.190 | 0.2340 | N.S. | N.S. |
| rs10046 | *CYP19A1* | 15:49290278 | am_10744 | 1.185 | 0.2360 | N.S. | N.S. |
| rs757110 | *ABCC8* | 11:17375053 | am_10270 | 1.183 | 0.2368 | N.S. | N.S. |
| rs7483 | *GSTM3* | 1:110100000 | am_11730 | -1.176 | 0.2396 | N.S. | N.S. |
| rs2235033 | *ABCB1* | 7:87017079 | am_14609 | 1.171 | 0.2416 | N.S. | N.S. |
| rs1056836 | *CYP1B1* | 2:38151707 | am_12461 | 1.170 | 0.2420 | N.S. | N.S. |
| rs1128503 | *ABCB1* | 7:87017537 | am_14612 | -1.163 | 0.2448 | N.S. | N.S. |
| rs9429505 | *SLC16A1* | 1:113300000 | am_11740 | 1.150 | 0.2501 | N.S. | N.S. |
| rs2301157 | *SLC10A2* | 13:102500000 | am_10675 | -1.145 | 0.2522 | N.S. | N.S. |
| rs2270860 | *SLC22A7* | 6:43378129 | am_14163 | -1.144 | 0.2526 | N.S. | N.S. |
| rs11249454 | *UGT2A1* | 4:70533823 | am_13558 | 1.144 | 0.2526 | N.S. | N.S. |
| rs1046428 | *GSTZ1* | 14:76864036 | am_10726 | 1.144 | 0.2526 | N.S. | N.S. |
| rs743572 | *CYP17A1* | 10:104600000 | am_10206 | 1.133 | 0.2572 | N.S. | N.S. |
| rs6163 | *CYP17A1* | 10:104600000 | am_10201 | 1.133 | 0.2572 | N.S. | N.S. |
| rs7697037 | *UGT2B11* | 4:70114564 | am_13478 | -1.122 | 0.2619 | N.S. | N.S. |
| rs4148946 | *CHST3* | 10:73440079 | am_10006 | -1.121 | 0.2623 | N.S. | N.S. |
| rs4148949 | *CHST3* | 10:73440657 | am_10010 | -1.121 | 0.2623 | N.S. | N.S. |
| rs1051332 | *ATP7B* | 13:51405721 | am_10588 | -1.117 | 0.2640 | N.S. | N.S. |
| rs2097937 | *CROT* | 7:86868839 | am_14528 | 1.110 | 0.2670 | N.S. | N.S. |
| rs279942 | *SLC10A2* | 13:102500000 | am_10672 | -1.105 | 0.2692 | N.S. | N.S. |
| rs1126671 | *ADH4* | 4:100300000 | am_13729 | 1.104 | 0.2696 | N.S. | N.S. |
| rs1126670 | *ADH4* | 4:100300000 | am_13730 | 1.104 | 0.2696 | N.S. | N.S. |
| rs1056837 | *CYP1B1* | 2:38151654 | am_12455 | 1.095 | 0.2735 | N.S. | N.S. |
| rs6521128 | *CHST7* | X:46330604 | am_15403 | 1.092 | 0.2748 | N.S. | N.S. |
| rs1873397 | *CHST13* | 3:127700000 | am_13326 | 1.089 | 0.2762 | N.S. | N.S. |
| rs1801243 | *ATP7B* | 13:51446141 | am_10596 | 1.089 | 0.2762 | N.S. | N.S. |
| rs3322 | *RALBP1* | 18:9526691 | am_11254 | 1.073 | 0.2833 | N.S. | N.S. |
| rs4148808 | *ABCB4* | 7:86943731 | am_14567 | -1.070 | 0.2846 | N.S. | N.S. |
| rs2074900 | *CYP4F2* | 19:15857820 | am_11300 | -1.065 | 0.2869 | N.S. | N.S. |
| rs3892097 | *CYP2D6* | 22:40854891 | am_12274 | -1.062 | 0.2882 | N.S. | N.S. |
| rs562 | *ABCC5* | 3:185100000 | am_13365 | 1.060 | 0.2891 | N.S. | N.S. |
| rs2952151 | *PNMT* | 17:35082022 | am_11219 | -1.057 | 0.2905 | N.S. | N.S. |
| rs1061040 | *SLC7A7* | 14:22312668 | am_10687 | -1.054 | 0.2919 | N.S. | N.S. |
| rs1105880 | *UGT1A9* | 2:234300000 | am_12973 | -1.049 | 0.2942 | N.S. | N.S. |
| rs4078 | *SLCO1A2* | 12:21368250 | am_10539 | -1.047 | 0.2951 | N.S. | N.S. |
| rs2276299 | *SLC22A8* | 11:62523007 | am_10361 | -1.046 | 0.2956 | N.S. | N.S. |
| rs4148805 | *ABCB4* | 7:86944301 | am_14570 | -1.045 | 0.2960 | N.S. | N.S. |
| rs3749442 | *ABCC5* | 3:185100000 | am_13377 | -1.041 | 0.2979 | N.S. | N.S. |
| rs1045642 | *ABCB1* | 7:86976581 | am_14581 | 1.033 | 0.3016 | N.S. | N.S. |
| rs10276036 | *ABCB1* | 7:87018134 | am_14617 | -1.032 | 0.3021 | N.S. | N.S. |
| rs3731722 | *AOX1* | 2:201200000 | am_12843 | -1.028 | 0.3039 | N.S. | N.S. |
| rs1060896 | *SLC28A2* | 15:43341559 | am_10730 | 1.028 | 0.3039 | N.S. | N.S. |
| rs2242048 | *SLC28A1* | 15:83279414 | am_10829 | 1.022 | 0.3068 | N.S. | N.S. |
| rs2288741 | *UGT2A1* | 4:70490067 | am_13534 | -1.021 | 0.3073 | N.S. | N.S. |
| rs2228099 | *ARNT* | 1:149100000 | am_11804 | 1.020 | 0.3077 | N.S. | N.S. |
| rs11731028 | *SULT1B1* | 4:70637429 | am_13600 | -1.016 | 0.3096 | N.S. | N.S. |
| rs10517 | *NQO1* | 16:68301261 | am_11078 | -1.014 | 0.3106 | N.S. | N.S. |
| rs2479390 | *GSTM5* | 1:110100000 | am_11719 | 1.011 | 0.3120 | N.S. | N.S. |
| rs2070673 | *CYP2E1* | 10:135200000 | am_10243 | -1.008 | 0.3135 | N.S. | N.S. |
| rs909921 | *TPSG1* | 16:1213804 | am_10913 | 1.004 | 0.3154 | N.S. | N.S. |
| rs55802895 | *NR1I3* | 1:159500000 | am_11835 | -1.004 | 0.3154 | N.S. | N.S. |
| rs6759892 | *UGT1A9* | 2:234300000 | am_12969 | -0.998 | 0.3183 | N.S. | N.S. |
| rs2283458 | *SLCO3A1* | 15:90490116 | am_10900 | 0.998 | 0.3183 | N.S. | N.S. |
| rs3736599 | *SULT1E1* | 4:70760410 | am_13637 | 0.996 | 0.3193 | N.S. | N.S. |
| rs2852425 | *NNMT* | 11:113700000 | am_10473 | 0.994 | 0.3202 | N.S. | N.S. |
| rs2738792 | *CHST5* | 16:74122785 | am_11130 | 0.991 | 0.3217 | N.S. | N.S. |
| rs1800566 | *NQO1* | 16:68302646 | am_11084 | -0.978 | 0.3281 | N.S. | N.S. |
| rs1799836 | *MAOB* | X:43512943 | am_15390 | -0.976 | 0.3291 | N.S. | N.S. |
| rs732316 | *CHST7* | X:46343567 | am_15408 | -0.976 | 0.3291 | N.S. | N.S. |
| rs6783962 | *CHST13* | 3:127700000 | am_13325 | 0.976 | 0.3291 | N.S. | N.S. |
| rs4680 | *COMT* | 22:18331271 | am_12229 | 0.974 | 0.3301 | N.S. | N.S. |
| rs1339067 | *SLC15A1* | 13:98154613 | am_10650 | 0.971 | 0.3315 | N.S. | N.S. |
| rs12680 | *RALBP1* | 18:9527835 | am_11255 | 0.970 | 0.3320 | N.S. | N.S. |
| rs3842 | *ABCB1* | 7:86971302 | am_14575 | -0.969 | 0.3325 | N.S. | N.S. |
| rs8025045 | *SLC28A1* | 15:83289576 | am_10839 | 0.963 | 0.3355 | N.S. | N.S. |
| rs2835286 | *CBR3* | 21:36440232 | am_12178 | -0.955 | 0.3396 | N.S. | N.S. |
| rs2287622 | *ABCB11* | 2:169500000 | am_12726 | -0.954 | 0.3401 | N.S. | N.S. |
| rs11796837 | *CHST7* | X:46334608 | am_15404 | -0.949 | 0.3426 | N.S. | N.S. |
| rs405729 | *GSTA4* | 6:52950740 | am_14321 | 0.945 | 0.3447 | N.S. | N.S. |
| rs735716 | *CHST7* | X:46342730 | am_15407 | -0.944 | 0.3452 | N.S. | N.S. |
| rs12542233 | *CYP7A1* | 8:59576955 | am_15048 | 0.923 | 0.3560 | N.S. | N.S. |
| rs732774 | *ATP7B* | 13:51421809 | am_10592 | 0.919 | 0.3581 | N.S. | N.S. |
| rs1801249 | *ATP7B* | 13:51413355 | am_10590 | 0.919 | 0.3581 | N.S. | N.S. |
| rs3808607 | *CYP7A1* | 8:59575478 | am_15047 | 0.912 | 0.3618 | N.S. | N.S. |
| rs2544794 | *SULT2B1* | 19:53771058 | am_11482 | -0.910 | 0.3628 | N.S. | N.S. |
| rs1056892 | *CBR3* | 21:36440576 | am_12181 | 0.908 | 0.3639 | N.S. | N.S. |
| rs7987433 | *SLC10A2* | 13:102500000 | am_10686 | -0.903 | 0.3665 | N.S. | N.S. |
| rs3832043 | *UGT1A9* | 2:234200000 | am_12947 | 0.903 | 0.3665 | N.S. | N.S. |
| rs6162 | *CYP17A1* | 10:104600000 | am_10202 | 0.898 | 0.3692 | N.S. | N.S. |
| rs1395 | *SLC5A6* | 2:27278140 | am_12327 | 0.894 | 0.3713 | N.S. | N.S. |
| rs9036 | *SLC6A6* | 3:14505725 | am_13153 | 0.893 | 0.3719 | N.S. | N.S. |
| rs2242416 | *SLC22A7* | 6:43381582 | am_14166 | -0.888 | 0.3745 | N.S. | N.S. |
| rs2301159 | *SLC10A2* | 13:102500000 | am_10671 | -0.887 | 0.3751 | N.S. | N.S. |
| rs2380563 | *SLCO5A1* | 8:70767626 | am_15113 | -0.882 | 0.3778 | N.S. | N.S. |
| rs2242046 | *SLC28A1* | 15:83279733 | am_10832 | -0.881 | 0.3783 | N.S. | N.S. |
| rs12727968 | *SLC16A1* | 1:113300000 | am_11739 | -0.876 | 0.3810 | N.S. | N.S. |
| rs12954 | *FMO1* | 1:169500000 | am_12028 | -0.874 | 0.3821 | N.S. | N.S. |
| rs31651 | *CROT* | 7:86867223 | am_14526 | 0.869 | 0.3848 | N.S. | N.S. |
| rs7957203 | *SLCO1A2* | 12:21368729 | am_10540 | 0.864 | 0.3876 | N.S. | N.S. |
| rs3828193 | *CHST10* | 2:100400000 | am_12535 | -0.853 | 0.3937 | N.S. | N.S. |
| rs7877 | *FMO1* | 1:169500000 | am_12031 | -0.852 | 0.3942 | N.S. | N.S. |
| rs2056822 | *CYP4F8* | 19:15600597 | am_11268 | -0.834 | 0.4043 | N.S. | N.S. |
| rs4239614 | *CYP4F8* | 19:15601220 | am_11269 | -0.834 | 0.4043 | N.S. | N.S. |
| rs8187858 | *ABCC1* | 16:16069540 | am_10928 | -0.833 | 0.4048 | N.S. | N.S. |
| rs274548 | *SLC22A5* | 5:131800000 | am_13959 | -0.832 | 0.4054 | N.S. | N.S. |
| rs13142440 | *UGT2B4* | 4:70381153 | am_13511 | -0.829 | 0.4071 | N.S. | N.S. |
| rs13119049 | *UGT2B4* | 4:70381154 | am_13513 | -0.829 | 0.4071 | N.S. | N.S. |
| rs1530030 | *CHST10* | 2:100400000 | am_12526 | -0.824 | 0.4099 | N.S. | N.S. |
| rs4646333 | *HNMT* | 2:138500000 | am_12647 | -0.816 | 0.4145 | N.S. | N.S. |
| rs1050891 | *HNMT* | 2:138500000 | am_12643 | -0.816 | 0.4145 | N.S. | N.S. |
| rs4245861 | *HNMT* | 2:138500000 | am_12646 | -0.816 | 0.4145 | N.S. | N.S. |
| rs4418728 | *CYP26A1* | 10:94829714 | am_10031 | -0.811 | 0.4174 | N.S. | N.S. |
| rs6987861 | *CYP7B1* | 8:65872021 | am_15086 | -0.801 | 0.4231 | N.S. | N.S. |
| rs1060463 | *CYP4F11* | 19:15886176 | am_11313 | 0.799 | 0.4243 | N.S. | N.S. |
| rs512795 | *GSTA3* | 6:52874942 | am_14306 | -0.797 | 0.4255 | N.S. | N.S. |
| rs1801244 | *ATP7B* | 13:51442806 | am_10595 | -0.791 | 0.4289 | N.S. | N.S. |
| rs11678615 | *AOX1* | 2:201200000 | am_12849 | -0.786 | 0.4319 | N.S. | N.S. |
| rs6965343 | *POR* | 7:75430861 | am_14490 | 0.772 | 0.4401 | N.S. | N.S. |
| rs3755320 | *UGT1A6* | 2:234300000 | am_12982 | 0.766 | 0.4437 | N.S. | N.S. |
| rs2231142 | *ABCG2* | 4:89271347 | am_13688 | -0.765 | 0.4443 | N.S. | N.S. |
| rs2302387 | *ABCB4* | 7:86930121 | am_14562 | -0.765 | 0.4443 | N.S. | N.S. |
| rs3765070 | *CYP4F11* | 19:15901292 | am_11317 | 0.760 | 0.4473 | N.S. | N.S. |
| rs2266782 | *FMO3* | 1:169300000 | am_11875 | -0.758 | 0.4485 | N.S. | N.S. |
| rs4543 | *CYP11B2* | 8:144000000 | am_15259 | -0.757 | 0.4490 | N.S. | N.S. |
| rs4148945 | *CHST3* | 10:73439596 | am_10005 | 0.756 | 0.4496 | N.S. | N.S. |
| rs41507953 | *EPHX2* | 8:27414422 | am_15017 | 0.754 | 0.4508 | N.S. | N.S. |
| rs2267667 | *PPARD* | 6:35480502 | am_14135 | -0.750 | 0.4533 | N.S. | N.S. |
| rs1867351 | *SLC22A1* | 6:160500000 | am_14347 | 0.748 | 0.4545 | N.S. | N.S. |
| rs7751481 | *PPARD* | 6:35479731 | am_14134 | -0.745 | 0.4563 | N.S. | N.S. |
| rs1883322 | *PPARD* | 6:35477784 | am_14132 | -0.745 | 0.4563 | N.S. | N.S. |
| rs1296954 | *GSTM5* | 1:110100000 | am_11710 | 0.734 | 0.4629 | N.S. | N.S. |
| rs998383 | *CBR1* | 21:36367609 | am_12172 | 0.719 | 0.4721 | N.S. | N.S. |
| rs4305381 | *CHST13* | 3:127700000 | am_13324 | -0.712 | 0.4765 | N.S. | N.S. |
| rs1801280 | *NAT2* | 8:18302134 | am_15001 | -0.712 | 0.4765 | N.S. | N.S. |
| rs7496 | *GSTA4* | 6:52950798 | am_14322 | -0.711 | 0.4771 | N.S. | N.S. |
| rs11684227 | *AOX1* | 2:201200000 | am_12848 | 0.708 | 0.4789 | N.S. | N.S. |
| rs3787537 | *SLCO4A1* | 20:60774187 | am_12165 | 0.702 | 0.4827 | N.S. | N.S. |
| rs12659 | *SLC19A1* | 21:45775984 | am_12214 | -0.693 | 0.4883 | N.S. | N.S. |
| rs10249788 | *AHR* | 7:17304672 | am_14464 | -0.690 | 0.4902 | N.S. | N.S. |
| rs886205 | *ALDH2* | 12:110700000 | am_10581 | 0.685 | 0.4933 | N.S. | N.S. |
| rs1805061 | *SLC7A7* | 14:22317952 | am_10691 | -0.681 | 0.4959 | N.S. | N.S. |
| rs7867504 | *SLC28A3* | 9:86110056 | am_15307 | -0.673 | 0.5009 | N.S. | N.S. |
| rs700518 | *CYP19A1* | 15:49316404 | am_10748 | 0.665 | 0.5061 | N.S. | N.S. |
| rs183574 | *SLC22A14* | 3:38338841 | am_13176 | -0.655 | 0.5125 | N.S. | N.S. |
| rs3814055 | *NR1I2* | 3:121000000 | am_13207 | 0.653 | 0.5138 | N.S. | N.S. |
| rs8058696 | *ABCC6* | 16:16186370 | am_10978 | -0.633 | 0.5267 | N.S. | N.S. |
| rs8058694 | *ABCC6* | 16:16186364 | am_10977 | -0.633 | 0.5267 | N.S. | N.S. |
| rs6729738 | *AOX1* | 2:201300000 | am_12852 | -0.631 | 0.5280 | N.S. | N.S. |
| rs4986993 | *NAT1* | 8:18125027 | am_14993 | 0.630 | 0.5287 | N.S. | N.S. |
| rs11859842 | *SPN* | 16:29568718 | am_11022 | 0.628 | 0.5300 | N.S. | N.S. |
| rs4148950 | *CHST3* | 10:73441712 | am_10011 | 0.624 | 0.5326 | N.S. | N.S. |
| rs7636910 | *ABCC5* | 3:185200000 | am_13400 | 0.620 | 0.5353 | N.S. | N.S. |
| rs1871450 | *CHST3* | 10:73442020 | am_10013 | 0.619 | 0.5359 | N.S. | N.S. |
| rs1045020 | *SLC22A5* | 5:131800000 | am_13958 | -0.618 | 0.5366 | N.S. | N.S. |
| rs533486 | *CYP3A43* | 7:99278630 | am_14856 | 0.618 | 0.5366 | N.S. | N.S. |
| rs2204295 | *SLC13A1* | 7:122600000 | am_14912 | -0.617 | 0.5372 | N.S. | N.S. |
| rs1126672 | *ADH4* | 4:100300000 | am_13727 | 0.614 | 0.5392 | N.S. | N.S. |
| rs4715333 | *GSTA1* | 6:52777208 | am_14287 | -0.613 | 0.5399 | N.S. | N.S. |
| rs1049434 | *SLC16A1* | 1:113300000 | am_11744 | -0.613 | 0.5399 | N.S. | N.S. |
| rs7169 | *SLC16A1* | 1:113300000 | am_11742 | -0.613 | 0.5399 | N.S. | N.S. |
| rs2109505 | *ABCB4* | 7:86917342 | am_14553 | -0.611 | 0.5412 | N.S. | N.S. |
| rs2032588 | *ABCB1* | 7:87017379 | am_14610 | 0.605 | 0.5452 | N.S. | N.S. |
| rs2297809 | *CYP4B1* | 1:47055359 | am_11554 | -0.603 | 0.5465 | N.S. | N.S. |
| rs624249 | *SLC22A2* | 6:160600000 | am_14401 | -0.602 | 0.5472 | N.S. | N.S. |
| rs2515641 | *CYP2E1* | 10:135200000 | am_10258 | -0.597 | 0.5505 | N.S. | N.S. |
| rs1799929 | *NAT2* | 8:18302274 | am_15006 | -0.592 | 0.5539 | N.S. | N.S. |
| rs1884545 | *SLC7A8* | 14:22721844 | am_10713 | 0.591 | 0.5545 | N.S. | N.S. |
| rs7563682 | *AOX1* | 2:201200000 | am_12847 | 0.580 | 0.5619 | N.S. | N.S. |
| rs2296241 | *CYP24A1* | 20:52219626 | am_12147 | 0.578 | 0.5633 | N.S. | N.S. |
| rs13251066 | *CYP7A1* | 8:59580307 | am_15050 | 0.578 | 0.5633 | N.S. | N.S. |
| rs4934027 | *MAT1A* | 10:82025540 | am_10025 | 0.574 | 0.5660 | N.S. | N.S. |
| rs9787901 | *CHST1* | 11:45652729 | am_10331 | 0.568 | 0.5700 | N.S. | N.S. |
| rs2066534 | *FMO3* | 1:169300000 | am_11880 | -0.563 | 0.5734 | N.S. | N.S. |
| rs4148551 | *ABCC4* | 13:94471519 | am_10603 | -0.554 | 0.5796 | N.S. | N.S. |
| rs3742106 | *ABCC4* | 13:94471792 | am_10604 | -0.554 | 0.5796 | N.S. | N.S. |
| rs7512729 | *CYP4Z1* | 1:47351058 | am_11611 | -0.548 | 0.5837 | N.S. | N.S. |
| rs4646 | *CYP19A1* | 15:49290136 | am_10742 | -0.545 | 0.5858 | N.S. | N.S. |
| rs4148943 | *CHST3* | 10:73439513 | am_10003 | 0.544 | 0.5864 | N.S. | N.S. |
| rs492338 | *ABCG1* | 21:42575046 | am_12204 | 0.542 | 0.5878 | N.S. | N.S. |
| rs688755 | *CYP4F12* | 19:15668305 | am_11291 | 0.541 | 0.5885 | N.S. | N.S. |
| rs7793861 | *CYP51A1* | 7:91580664 | am_14666 | 0.541 | 0.5885 | N.S. | N.S. |
| rs1050152 | *SLC22A4* | 5:131700000 | am_13903 | -0.537 | 0.5913 | N.S. | N.S. |
| rs1048977 | *CDA* | 1:20817642 | am_11520 | -0.534 | 0.5933 | N.S. | N.S. |
| rs3093105 | *CYP4F2* | 19:15869388 | am_11310 | 0.534 | 0.5933 | N.S. | N.S. |
| rs131713 | *ARSA* | 22:49415426 | am_12322 | -0.527 | 0.5982 | N.S. | N.S. |
| rs5297 | *CYP11B1* | 8:144000000 | am_15226 | -0.526 | 0.5989 | N.S. | N.S. |
| rs3100 | *UGT2B15* | 4:69547273 | am_13442 | -0.524 | 0.6003 | N.S. | N.S. |
| rs1056522 | *CHST13* | 3:127700000 | am_13333 | 0.510 | 0.6101 | N.S. | N.S. |
| rs2268877 | *SLC7A8* | 14:22706597 | am_10710 | -0.510 | 0.6101 | N.S. | N.S. |
| rs212090 | *ABCC1* | 16:16143505 | am_10944 | 0.508 | 0.6115 | N.S. | N.S. |
| rs6785049 | *NR1I2* | 3:121000000 | am_13253 | -0.506 | 0.6129 | N.S. | N.S. |
| rs593421 | *CYP4F12* | 19:15668830 | am_11296 | 0.505 | 0.6136 | N.S. | N.S. |
| rs11231825 | *SLC22A12* | 11:64116850 | am_10401 | -0.499 | 0.6178 | N.S. | N.S. |
| rs2238472 | *ABCC6* | 16:16159100 | am_10959 | -0.494 | 0.6213 | N.S. | N.S. |
| rs1154400 | *ADH5* | 4:100200000 | am_13717 | 0.492 | 0.6227 | N.S. | N.S. |
| rs683369 | *SLC22A1* | 6:160500000 | am_14351 | 0.489 | 0.6248 | N.S. | N.S. |
| rs1799853 | *CYP2C9* | 10:96692037 | am_10100 | 0.483 | 0.6291 | N.S. | N.S. |
| rs1801265 | *DPYD* | 1:98121473 | am_11669 | 0.483 | 0.6291 | N.S. | N.S. |
| rs887241 | *ALDH3A1* | 17:19586530 | am_11205 | 0.481 | 0.6305 | N.S. | N.S. |
| rs2268873 | *SLC7A8* | 14:22710634 | am_10711 | -0.479 | 0.6319 | N.S. | N.S. |
| rs2223477 | *FMO4* | 1:169600000 | am_12058 | 0.474 | 0.6355 | N.S. | N.S. |
| rs3869579 | *CYP2A7* | 19:46075639 | am_11376 | 0.467 | 0.6405 | N.S. | N.S. |
| rs2297810 | *CYP4B1* | 1:47053446 | am_11549 | -0.466 | 0.6412 | N.S. | N.S. |
| rs11807 | *GSTM5* | 1:110100000 | am_11724 | 0.465 | 0.6419 | N.S. | N.S. |
| rs717620 | *ABCC2* | 10:101500000 | am_10143 | -0.461 | 0.6448 | N.S. | N.S. |
| rs2277119 | *CYP39A1* | 6:46717864 | am_14216 | -0.460 | 0.6455 | N.S. | N.S. |
| rs149738 | *SLC22A14* | 3:38342988 | am_13180 | -0.459 | 0.6462 | N.S. | N.S. |
| rs31652 | *CROT* | 7:86867623 | am_14527 | 0.456 | 0.6484 | N.S. | N.S. |
| rs4668115 | *ABCB11* | 2:169600000 | am_12783 | -0.455 | 0.6491 | N.S. | N.S. |
| rs171248 | *SLC22A14* | 3:38338397 | am_13175 | -0.451 | 0.6520 | N.S. | N.S. |
| rs9282861 | *SULT1A1* | 16:28525015 | am_11005 | 0.449 | 0.6534 | N.S. | N.S. |
| rs11401 | *SULT1A2_A3* | 16:28510492 | am_10985 | 0.448 | 0.6542 | N.S. | N.S. |
| rs2273697 | *ABCC2* | 10:101600000 | am_10152 | 0.443 | 0.6578 | N.S. | N.S. |
| rs1051640 | *ABCC3* | 17:46123485 | am_11243 | 0.442 | 0.6585 | N.S. | N.S. |
| rs2641806 | *CHST5* | 16:74122921 | am_11131 | 0.442 | 0.6585 | N.S. | N.S. |
| rs4783745 | *CES2* | 16:65528476 | am_11066 | 0.438 | 0.6614 | N.S. | N.S. |
| rs9930567 | *RPL13* | 16:88155574 | am_11168 | -0.424 | 0.6716 | N.S. | N.S. |
| rs1709082 | *CYP2A13* | 19:46293449 | am_11449 | 0.417 | 0.6767 | N.S. | N.S. |
| rs7541966 | *FMO4* | 1:169600000 | am_12057 | 0.412 | 0.6803 | N.S. | N.S. |
| rs1041983 | *NAT2* | 8:18302075 | am_15000 | -0.412 | 0.6803 | N.S. | N.S. |
| rs2842934 | *TPMT* | 6:18247193 | am_13979 | 0.410 | 0.6818 | N.S. | N.S. |
| rs7586110 | *UGT1A1* | 2:234300000 | am_12956 | -0.409 | 0.6825 | N.S. | N.S. |
| rs1135840 | *CYP2D6* | 22:40852557 | am_12247 | -0.401 | 0.6884 | N.S. | N.S. |
| rs1058164 | *CYP2D6* | 22:40855076 | am_12277 | -0.400 | 0.6892 | N.S. | N.S. |
| rs6196 | *NR3C1* | 5:142600000 | am_13963 | 0.399 | 0.6899 | N.S. | N.S. |
| rs2295475 | *XDH* | 2:31443351 | am_12385 | 0.397 | 0.6914 | N.S. | N.S. |
| rs5629 | *PTGIS* | 20:47563113 | am_12126 | 0.397 | 0.6914 | N.S. | N.S. |
| rs2070959 | *UGT1A6* | 2:234300000 | am_12974 | -0.389 | 0.6973 | N.S. | N.S. |
| rs8133052 | *CBR3* | 21:36429371 | am_12174 | -0.386 | 0.6995 | N.S. | N.S. |
| rs496550 | *ABCB11* | 2:169500000 | am_12655 | 0.385 | 0.7002 | N.S. | N.S. |
| rs3821242 | *UGT1A9* | 2:234300000 | am_13005 | 0.385 | 0.7002 | N.S. | N.S. |
| rs4149057 | *SLCO1B1* | 12:21222866 | am_10501 | -0.382 | 0.7025 | N.S. | N.S. |
| rs1966151 | *UGT2B4* | 4:70380716 | am_13509 | -0.381 | 0.7032 | N.S. | N.S. |
| rs887829 | *UGT1A1* | 2:234300000 | am_13022 | -0.375 | 0.7077 | N.S. | N.S. |
| rs1976391 | *UGT1A1* | 2:234300000 | am_13020 | -0.375 | 0.7077 | N.S. | N.S. |
| rs7797834 | *CYP51A1* | 7:91581086 | am_14667 | 0.372 | 0.7099 | N.S. | N.S. |
| rs4808326 | *CYP4F8* | 19:15587147 | am_11263 | -0.370 | 0.7114 | N.S. | N.S. |
| rs6980478 | *CYP7B1* | 8:65883288 | am_15090 | 0.366 | 0.7144 | N.S. | N.S. |
| rs6706232 | *UGT1A3* | 2:234300000 | am_13007 | 0.365 | 0.7151 | N.S. | N.S. |
| rs1541290 | *ABCG1* | 21:42591552 | am_12210 | 0.365 | 0.7151 | N.S. | N.S. |
| rs7785971 | *AKAP9* | 7:91574730 | am_14661 | 0.363 | 0.7166 | N.S. | N.S. |
| rs731027 | *CHST3* | 10:73442342 | am_10014 | 0.361 | 0.7181 | N.S. | N.S. |
| rs730720 | *CHST3* | 10:73442768 | am_10016 | 0.361 | 0.7181 | N.S. | N.S. |
| rs762551 | *CYP1A2* | 15:72828970 | am_10785 | 0.359 | 0.7196 | N.S. | N.S. |
| rs3740066 | *ABCC2* | 10:101600000 | am_10183 | 0.358 | 0.7203 | N.S. | N.S. |
| rs7574296 | *UGT1A9* | 2:234300000 | am_13011 | 0.356 | 0.7218 | N.S. | N.S. |
| rs316003 | *SLC22A2* | 6:160600000 | am_14386 | 0.350 | 0.7263 | N.S. | N.S. |
| rs2290272 | *SLC28A1* | 15:83248435 | am_10816 | -0.345 | 0.7301 | N.S. | N.S. |
| rs2272797 | *FMO6* | 1:169400000 | am_11913 | 0.340 | 0.7339 | N.S. | N.S. |
| rs6962039 | *SLC13A1* | 7:122500000 | am_14871 | -0.339 | 0.7346 | N.S. | N.S. |
| rs17064 | *ABCB1* | 7:86971406 | am_14577 | -0.333 | 0.7391 | N.S. | N.S. |
| rs4079369 | *CYP2A6* | 19:46044602 | am_11342 | -0.332 | 0.7399 | N.S. | N.S. |
| rs8192729 | *CYP2A6* | 19:46042836 | am_11334 | -0.332 | 0.7399 | N.S. | N.S. |
| rs8192879 | *CYP7A1* | 8:59566130 | am_15037 | 0.331 | 0.7406 | N.S. | N.S. |
| rs34815109 | *UGT1A1* | 2:234300000 | am_13024 | -0.331 | 0.7406 | N.S. | N.S. |
| rs13226149 | *PON3* | 7:94863536 | am_14702 | -0.326 | 0.7444 | N.S. | N.S. |
| rs246221 | *ABCC1* | 16:16045823 | am_10920 | 0.326 | 0.7444 | N.S. | N.S. |
| rs2190748 | *SLCO3A1* | 15:90486082 | am_10899 | -0.325 | 0.7452 | N.S. | N.S. |
| rs1062033 | *CYP19A1* | 15:49335230 | am_10750 | -0.319 | 0.7497 | N.S. | N.S. |
| rs1143671 | *SLC15A2* | 3:123100000 | am_13306 | 0.316 | 0.7520 | N.S. | N.S. |
| rs7886938 | *FMO6* | 1:169400000 | am_11915 | 0.314 | 0.7535 | N.S. | N.S. |
| rs7889839 | *FMO6* | 1:169400000 | am_11914 | 0.314 | 0.7535 | N.S. | N.S. |
| rs2076167 | *PPARD* | 6:35499765 | am_14149 | -0.310 | 0.7566 | N.S. | N.S. |
| rs3755319 | *UGT1A1* | 2:234300000 | am_13021 | 0.309 | 0.7573 | N.S. | N.S. |
| rs11770903 | *PON3* | 7:94864263 | am_14706 | -0.308 | 0.7581 | N.S. | N.S. |
| rs1880179 | *SLC13A1* | 7:122600000 | am_14913 | -0.307 | 0.7588 | N.S. | N.S. |
| rs2470890 | *CYP1A2* | 15:72834479 | am_10807 | -0.304 | 0.7611 | N.S. | N.S. |
| rs1805343 | *RXRA* | 9:136500000 | am_15349 | 0.304 | 0.7611 | N.S. | N.S. |
| rs305968 | *CYP2F1* | 19:46314029 | am_11456 | -0.302 | 0.7627 | N.S. | N.S. |
| rs1048013 | *CYP20A1* | 2:203900000 | am_12857 | 0.301 | 0.7634 | N.S. | N.S. |
| rs1060253 | *SLC7A5* | 16:86423639 | am_11135 | 0.301 | 0.7634 | N.S. | N.S. |
| rs895729 | *CHST1* | 11:45635773 | am_10321 | 0.300 | 0.7642 | N.S. | N.S. |
| rs2140516 | *SLC13A1* | 7:122600000 | am_14896 | 0.299 | 0.7649 | N.S. | N.S. |
| rs2281891 | *CYP2C18* | 10:96483048 | am_10047 | -0.297 | 0.7665 | N.S. | N.S. |
| rs495714 | *ABCB11* | 2:169500000 | am_12656 | 0.296 | 0.7672 | N.S. | N.S. |
| rs2884737 | *VKORC1* | 16:31013055 | am_11049 | 0.295 | 0.7680 | N.S. | N.S. |
| rs2227291 | *ATP7A* | X:77155158 | am_15454 | -0.292 | 0.7703 | N.S. | N.S. |
| rs2762934 | *CYP24A1* | 20:52204668 | am_12140 | 0.287 | 0.7741 | N.S. | N.S. |
| rs10008281 | *ADH6* | 4:100400000 | am_13760 | -0.286 | 0.7749 | N.S. | N.S. |
| rs2292566 | *EPHX1* | 1:224100000 | am_12093 | -0.285 | 0.7756 | N.S. | N.S. |
| rs1799930 | *NAT2* | 8:18302383 | am_15008 | -0.284 | 0.7764 | N.S. | N.S. |
| rs11764079 | *PON3* | 7:94864165 | am_14705 | -0.283 | 0.7772 | N.S. | N.S. |
| rs939336 | *ABCC5* | 3:185200000 | am_13393 | 0.281 | 0.7787 | N.S. | N.S. |
| rs4679028 | *SLC22A13* | 3:38302296 | am_13166 | 0.280 | 0.7795 | N.S. | N.S. |
| rs4124874 | *UGT1A1* | 2:234300000 | am_13018 | 0.279 | 0.7802 | N.S. | N.S. |
| rs296365 | *SULT2A1* | 19:53066363 | am_11474 | -0.270 | 0.7872 | N.S. | N.S. |
| rs13265049 | *CYP7B1* | 8:65864694 | am_15085 | 0.269 | 0.7879 | N.S. | N.S. |
| rs1126692 | *FMO1* | 1:169500000 | am_12023 | -0.266 | 0.7902 | N.S. | N.S. |
| rs742350 | *FMO1* | 1:169500000 | am_12017 | -0.266 | 0.7902 | N.S. | N.S. |
| rs17685 | *POR* | 7:75454041 | am_14518 | -0.255 | 0.7987 | N.S. | N.S. |
| rs8187758 | *SLC28A1* | 15:83249879 | am_10820 | 0.251 | 0.8018 | N.S. | N.S. |
| rs4148553 | *ABCC4* | 13:94471136 | am_10602 | -0.250 | 0.8026 | N.S. | N.S. |
| rs1059751 | *ABCC4* | 13:94470951 | am_10601 | -0.250 | 0.8026 | N.S. | N.S. |
| rs7141505 | *SLC7A8* | 14:22723028 | am_10714 | -0.248 | 0.8041 | N.S. | N.S. |
| rs4925 | *GSTO1* | 10:106000000 | am_10221 | -0.247 | 0.8049 | N.S. | N.S. |
| rs11211402 | *CYP4A11* | 1:47164641 | am_11564 | 0.241 | 0.8096 | N.S. | N.S. |
| rs2276707 | *NR1I2* | 3:121000000 | am_13256 | -0.240 | 0.8103 | N.S. | N.S. |
| rs909530 | *FMO3* | 1:169300000 | am_11888 | 0.236 | 0.8134 | N.S. | N.S. |
| rs6068816 | *CYP24A1* | 20:52214498 | am_12145 | -0.230 | 0.8181 | N.S. | N.S. |
| rs2078267 | *SLC22A11* | 11:64090690 | am_10390 | 0.230 | 0.8181 | N.S. | N.S. |
| rs2053098 | *SLCO1B3* | 12:20927678 | am_10487 | 0.229 | 0.8189 | N.S. | N.S. |
| rs7311358 | *SLCO1B3* | 12:20907027 | am_10482 | 0.229 | 0.8189 | N.S. | N.S. |
| rs4149117 | *SLCO1B3* | 12:20902747 | am_10481 | 0.229 | 0.8189 | N.S. | N.S. |
| rs367836 | *GSTA4* | 6:52951090 | am_14324 | 0.224 | 0.8228 | N.S. | N.S. |
| rs1143670 | *SLC15A2* | 3:123100000 | am_13301 | 0.222 | 0.8243 | N.S. | N.S. |
| rs2279344 | *CYP2B6* | 19:46207323 | am_11416 | -0.217 | 0.8282 | N.S. | N.S. |
| rs668871 | *SLC22A3* | 6:160700000 | am_14410 | -0.215 | 0.8298 | N.S. | N.S. |
| rs3215983 | *CYP4B1* | 1:47053334 | am_11546 | -0.212 | 0.8321 | N.S. | N.S. |
| rs4646491 | *CYP4B1* | 1:47053471 | am_11550 | -0.212 | 0.8321 | N.S. | N.S. |
| rs4244285 | *CYP2C19* | 10:96531606 | am_10070 | -0.210 | 0.8337 | N.S. | N.S. |
| rs1208 | *NAT2* | 8:18302596 | am_15010 | -0.206 | 0.8368 | N.S. | N.S. |
| rs2602836 | *ADH5* | 4:100200000 | am_13720 | 0.205 | 0.8376 | N.S. | N.S. |
| rs2293616 | *SLC15A2* | 3:123100000 | am_13298 | 0.204 | 0.8384 | N.S. | N.S. |
| rs3775770 | *SULT1E1* | 4:70758859 | am_13634 | 0.203 | 0.8391 | N.S. | N.S. |
| rs13197674 | *GSTA4* | 6:52968366 | am_14336 | 0.203 | 0.8391 | N.S. | N.S. |
| rs2305367 | *SLC28A1* | 15:83277445 | am_10825 | 0.203 | 0.8391 | N.S. | N.S. |
| rs2856585 | *ABCC6* | 16:16171164 | am_10964 | -0.200 | 0.8415 | N.S. | N.S. |
| rs1736565 | *FMO6* | 1:169400000 | am_11912 | -0.199 | 0.8423 | N.S. | N.S. |
| rs1143672 | *SLC15A2* | 3:123100000 | am_13307 | 0.198 | 0.8430 | N.S. | N.S. |
| rs2257212 | *SLC15A2* | 3:123100000 | am_13300 | 0.198 | 0.8430 | N.S. | N.S. |
| rs2266780 | *FMO3* | 1:169300000 | am_11891 | -0.196 | 0.8446 | N.S. | N.S. |
| rs1202283 | *ABCB4* | 7:86920228 | am_14556 | -0.194 | 0.8462 | N.S. | N.S. |
| rs2341970 | *SLC6A6* | 3:14449229 | am_13148 | 0.185 | 0.8532 | N.S. | N.S. |
| rs12512110 | *ADH1A* | 4:100400000 | am_13764 | -0.183 | 0.8548 | N.S. | N.S. |
| rs473351 | *ABCB11* | 2:169500000 | am_12658 | 0.168 | 0.8666 | N.S. | N.S. |
| rs4715354 | *GSTA5* | 6:52816756 | am_14296 | 0.166 | 0.8682 | N.S. | N.S. |
| rs8050894 | *VKORC1* | 16:31012010 | am_11043 | -0.163 | 0.8705 | N.S. | N.S. |
| rs9934438 | *VKORC1* | 16:31012379 | am_11045 | 0.163 | 0.8705 | N.S. | N.S. |
| rs3787728 | *CBR1* | 21:36365763 | am_12170 | -0.161 | 0.8721 | N.S. | N.S. |
| rs3770602 | *ABCB11* | 2:169600000 | am_12780 | -0.160 | 0.8729 | N.S. | N.S. |
| rs212091 | *ABCC1* | 16:16144151 | am_10947 | 0.156 | 0.8760 | N.S. | N.S. |
| rs750398 | *CHST1* | 11:45653648 | am_10333 | -0.154 | 0.8776 | N.S. | N.S. |
| rs3762894 | *ADH4* | 4:100300000 | am_13741 | -0.151 | 0.8800 | N.S. | N.S. |
| rs10898 | *RALBP1* | 18:9526249 | am_11253 | 0.130 | 0.8966 | N.S. | N.S. |
| rs9923231 | *VKORC1* | 16:31015190 | am_11054 | 0.128 | 0.8981 | N.S. | N.S. |
| rs1783811 | *SLC22A11* | 11:64089872 | am_10389 | -0.122 | 0.9029 | N.S. | N.S. |
| rs1799735 | *GSTM3* | 1:110100000 | am_11734 | 0.114 | 0.9092 | N.S. | N.S. |
| rs13959 | *ALDH1A1* | 9:74735702 | am_15285 | 0.112 | 0.9108 | N.S. | N.S. |
| rs279941 | *SLC10A2* | 13:102500000 | am_10673 | -0.111 | 0.9116 | N.S. | N.S. |
| rs188096 | *SLC10A2* | 13:102500000 | am_10678 | -0.111 | 0.9116 | N.S. | N.S. |
| rs9285726 | *MAT1A* | 10:82025130 | am_10023 | -0.104 | 0.9172 | N.S. | N.S. |
| rs7602171 | *ABCB11* | 2:169600000 | am_12778 | -0.104 | 0.9172 | N.S. | N.S. |
| rs1801282 | *PPARG* | 3:12368125 | am_13097 | -0.103 | 0.9180 | N.S. | N.S. |
| rs17882539 | *PON3* | 7:94864344 | am_14707 | -0.099 | 0.9211 | N.S. | N.S. |
| rs316019 | *SLC22A2* | 6:160600000 | am_14395 | 0.093 | 0.9259 | N.S. | N.S. |
| rs497692 | *ABCB11* | 2:169500000 | am_12674 | -0.079 | 0.9370 | N.S. | N.S. |
| rs1051740 | *EPHX1* | 1:224100000 | am_12092 | 0.077 | 0.9386 | N.S. | N.S. |
| rs4149056 | *SLCO1B1* | 12:21222816 | am_10500 | 0.077 | 0.9386 | N.S. | N.S. |
| rs2236553 | *SLCO4A1* | 20:60760188 | am_12160 | -0.075 | 0.9402 | N.S. | N.S. |
| rs2292334 | *SLC22A3* | 6:160800000 | am_14444 | 0.070 | 0.9442 | N.S. | N.S. |
| rs2501870 | *NR1I3* | 1:159500000 | am_11834 | 0.068 | 0.9458 | N.S. | N.S. |
| rs1137070 | *MAOA* | X:43488335 | am_15380 | 0.066 | 0.9474 | N.S. | N.S. |
| rs628031 | *SLC22A1* | 6:160500000 | am_14363 | -0.065 | 0.9482 | N.S. | N.S. |
| rs2236135 | *SLC7A8* | 14:22665561 | am_10698 | -0.063 | 0.9498 | N.S. | N.S. |
| rs953062 | *CYP39A1* | 6:46734312 | am_14227 | 0.059 | 0.9530 | N.S. | N.S. |
| rs890293 | *CYP2J2* | 1:60165082 | am_11637 | -0.058 | 0.9537 | N.S. | N.S. |
| rs2359612 | *VKORC1* | 16:31011297 | am_11040 | -0.057 | 0.9545 | N.S. | N.S. |
| rs9381468 | *CYP39A1* | 6:46733233 | am_14226 | -0.055 | 0.9561 | N.S. | N.S. |
| rs5085 | *NR1I3* | 1:159500000 | am_11809 | 0.054 | 0.9569 | N.S. | N.S. |
| rs8013529 | *SLC7A8* | 14:22719632 | am_10712 | 0.054 | 0.9569 | N.S. | N.S. |
| rs7294 | *VKORC1* | 16:31009822 | am_11034 | 0.049 | 0.9609 | N.S. | N.S. |
| rs3093106 | *CYP4F2* | 19:15869257 | am_11307 | -0.045 | 0.9641 | N.S. | N.S. |
| rs13331798 | *QPRT* | 16:29654792 | am_11026 | -0.036 | 0.9713 | N.S. | N.S. |
| rs7761731 | *CYP39A1* | 6:46671776 | am_14196 | -0.032 | 0.9745 | N.S. | N.S. |
| rs425215 | *ABCG1* | 21:42580170 | am_12206 | 0.031 | 0.9753 | N.S. | N.S. |
| rs1517618 | *SLCO3A1* | 15:90448649 | am_10890 | 0.029 | 0.9769 | N.S. | N.S. |
| rs1152003 | *PPARG* | 3:12452055 | am_13140 | -0.026 | 0.9793 | N.S. | N.S. |
| rs17708472 | *VKORC1* | 16:31012854 | am_11047 | -0.025 | 0.9801 | N.S. | N.S. |
| rs2072330 | *ALDH3A1* | 17:19585064 | am_11203 | -0.022 | 0.9824 | N.S. | N.S. |
| rs6771233 | *CYP8B1* | 3:42897701 | am_13191 | 0.020 | 0.9840 | N.S. | N.S. |
| rs6774801 | *CYP8B1* | 3:42898780 | am_13192 | 0.020 | 0.9840 | N.S. | N.S. |
| rs1131878 | *UGT2B4* | 4:70380493 | am_13507 | -0.019 | 0.9848 | N.S. | N.S. |
| rs1138272 | *GSTP1* | 11:67110155 | am_10442 | -0.016 | 0.9872 | N.S. | N.S. |
| rs10929302 | *UGT1A1* | 2:234300000 | am_13019 | 0.015 | 0.9880 | N.S. | N.S. |
| rs2108622 | *CYP4F2* | 19:15851431 | am_11299 | -0.011 | 0.9912 | N.S. | N.S. |
| rs5909 | *HMGCR* | 5:74691931 | am_13871 | -0.009 | 0.9928 | N.S. | N.S. |
| rs2298419 | *SLC22A13* | 3:38297630 | am_13163 | 0.005 | 0.9960 | N.S. | N.S. |
| rs3784932 | *CHST5* | 16:74121788 | am_11128 | 0.004 | 0.9968 | N.S. | N.S. |
| rs4148269 | *UGT2B15* | 4:69195442 | am_13437 | 0.002 | 0.9984 | N.S. | N.S. |
| rs4148269 | *UGT2B15* | 4:69547466 | am_13444 | -0.001 | 0.9992 | N.S. | N.S. |
