# Supplementary material for: Drug Metabolizing Enzyme and Transporter Gene Variation, Nicotine Metabolism, Prospective Abstinence, and Cigarette Consumption
Source: PLoS One. 2015 Jul 1;10(7):e0126113. doi: 10.1371/journal.pone.0126113 (PMC4488893; doi:10.1371/journal.pone.0126113)
Supplement: S3 Table — (DOCX) [file pone.0126113.s003.docx]

**Supplementary Table 3.** DMET SNP Meta-P_ACT_ Gene-wise Results in PKTWIN and SMOFAM.

| ***Gene*** | ***P*-value** | ***Gene*** | ***P*-value** | ***Gene*** | ***P*-value** | ***Gene*** | ***P*-value** |
| --- | --- | --- | --- | --- | --- | --- | --- |
| *CYP2A6* | 4.05E-07 | *CHST11* | 1.71E-01 | *CYP11B2* | 4.49E-01 | *UGT1A6* | 6.88E-01 |
| *CYP2D6* | 1.03E-03 | *UGT1A1* | 1.81E-01 | *EPHX2* | 4.51E-01 | *NR3C1* | 6.90E-01 |
| *SPG7* | 1.08E-02 | *GSTM4* | 1.86E-01 | *HNMT* | 4.62E-01 | *PTGIS* | 6.92E-01 |
| *XDH* | 1.11E-02 | *CYP8B1* | 1.95E-01 | *SLCO1A2* | 4.62E-01 | *UGT1A3* | 7.15E-01 |
| *CHST8* | 1.49E-02 | *UGT2B15* | 2.06E-01 | *CYP19A1* | 4.70E-01 | *GSTA4* | 7.29E-01 |
| *CHST13* | 2.46E-02 | *CYP4F8* | 2.07E-01 | *SLC22A5* | 4.84E-01 | *SLCO4A1* | 7.33E-01 |
| *SLCO1B1* | 2.46E-02 | *CDA* | 2.13E-01 | *AHR* | 4.90E-01 | *UGT2B4* | 7.33E-01 |
| *CYP4F3* | 2.66E-02 | *ABCC8* | 2.37E-01 | *ALDH2* | 4.94E-01 | *RXRA* | 7.61E-01 |
| *SLC15A1* | 4.42E-02 | *ABCC3* | 2.59E-01 | *SLCO3A1* | 4.95E-01 | *CYP2F1* | 7.63E-01 |
| *CBR1* | 4.76E-02 | *CHST10* | 2.60E-01 | *ADH4* | 5.15E-01 | *CYP20A1* | 7.63E-01 |
| *SULT1C2* | 5.47E-02 | *UGT2B11* | 2.62E-01 | *UGT2A1* | 5.16E-01 | *SLC7A5* | 7.64E-01 |
| *SULT1E1* | 5.84E-02 | *CYP1B1* | 2.65E-01 | *NQO1* | 5.24E-01 | *ABCB11* | 7.65E-01 |
| *ABCC4* | 6.11E-02 | *CYP4Z1* | 2.67E-01 | *NAT1* | 5.29E-01 | *SLC28A1* | 7.69E-01 |
| *CYP26A1* | 6.80E-02 | *SLC22A4* | 2.82E-01 | *CYP3A43* | 5.37E-01 | *ATP7A* | 7.71E-01 |
| *ADH6* | 7.11E-02 | *PNMT* | 2.91E-01 | *NR1I3* | 5.40E-01 | *SULT2A1* | 7.87E-01 |
| *SLCO5A1* | 7.13E-02 | *ARSA* | 2.92E-01 | *SLC16A1* | 5.61E-01 | *FMO3* | 7.96E-01 |
| *GSTP1* | 7.14E-02 | *SULT1A2_A3* | 2.94E-01 | *ABCB4* | 5.64E-01 | *ADH5* | 7.98E-01 |
| *CHST2* | 7.67E-02 | *SLC22A8* | 2.96E-01 | *SLC22A14* | 5.83E-01 | *GSTO1* | 8.05E-01 |
| *CYP2C18* | 8.03E-02 | *SLC28A2* | 3.04E-01 | *CHST5* | 5.84E-01 | *CYP4A11* | 8.09E-01 |
| *ABCG1* | 8.42E-02 | *ARNT* | 3.08E-01 | *CHST7* | 5.93E-01 | *NAT2* | 8.18E-01 |
| *SLC22A1* | 8.90E-02 | *ATP7B* | 3.10E-01 | *ABCC5* | 5.96E-01 | *SLC15A2* | 8.40E-01 |
| *CYP2C19* | 8.97E-02 | *CYP17A1* | 3.14E-01 | *SLC6A6* | 6.05E-01 | *ALDH3A1* | 8.44E-01 |
| *CYP2B6* | 8.99E-02 | *TPSG1* | 3.15E-01 | *UGT1A9* | 6.10E-01 | *CYP1A2* | 8.59E-01 |
| *ABP1* | 1.07E-01 | *NNMT* | 3.20E-01 | *CBR3* | 6.15E-01 | *NR1I2* | 8.61E-01 |
| *SULT1B1* | 1.09E-01 | *GSTA1* | 3.29E-01 | *CYP4F2* | 6.17E-01 | *FMO4* | 8.62E-01 |
| *SULT2B1* | 1.09E-01 | *MAOB* | 3.29E-01 | *CYP7A1* | 6.18E-01 | *GSTA5* | 8.68E-01 |
| *MAT1A* | 1.11E-01 | *PPARD* | 3.41E-01 | *SLC22A12* | 6.18E-01 | *SLC22A2* | 8.79E-01 |
| *GSTA2* | 1.12E-01 | *ABCC1* | 3.51E-01 | *CYP7B1* | 6.25E-01 | *SLCO1B3* | 8.89E-01 |
| *ADH1A* | 1.15E-01 | *AKAP9* | 3.71E-01 | *CYP2C9* | 6.29E-01 | *ABCC6* | 8.96E-01 |
| *SLC19A1* | 1.18E-01 | *SLC5A6* | 3.72E-01 | *RALBP1* | 6.31E-01 | *SLC13A1* | 9.10E-01 |
| *UGT2B7* | 1.20E-01 | *CHST1* | 3.73E-01 | *GSTM5* | 6.33E-01 | *ALDH1A1* | 9.11E-01 |
| *CYP4F11* | 1.33E-01 | *GSTZ1* | 3.78E-01 | *CYP51A1* | 6.33E-01 | *ABCC2* | 9.41E-01 |
| *CYP24A1* | 1.36E-01 | *SLC22A7* | 3.83E-01 | *CYP4F12* | 6.35E-01 | *FMO6* | 9.47E-01 |
| *PON1* | 1.37E-01 | *DPYD* | 3.84E-01 | *CYP2A7* | 6.41E-01 | *MAOA* | 9.47E-01 |
| *SLC7A7* | 1.43E-01 | *CYP11B1* | 3.87E-01 | *AOX1* | 6.42E-01 | *SLC22A13* | 9.48E-01 |
| *PON3* | 1.50E-01 | *FMO2* | 3.91E-01 | *POR* | 6.44E-01 | *EPHX1* | 9.49E-01 |
| *ADH1C* | 1.51E-01 | *SLC10A2* | 4.08E-01 | *SULT1A1* | 6.54E-01 | *CYP2J2* | 9.54E-01 |
| *ALB* | 1.52E-01 | *GSTM3* | 4.10E-01 | *CES2* | 6.62E-01 | *SLC22A11* | 9.55E-01 |
| *ABCB1* | 1.53E-01 | *CHST3* | 4.19E-01 | *CYP4B1* | 6.65E-01 | *SLC22A3* | 9.64E-01 |
| *SULT4A1* | 1.57E-01 | *CYP2E1* | 4.23E-01 | *RPL13* | 6.72E-01 | *CYP39A1* | 9.75E-01 |
| *SLC28A3* | 1.64E-01 | *GSTA3* | 4.26E-01 | *FMO1* | 6.73E-01 | *SLC7A8* | 9.79E-01 |
| *COMT* | 1.67E-01 | *ABCG2* | 4.44E-01 | *CYP2A13* | 6.77E-01 | *VKORC1* | 9.91E-01 |
| *FAAH* | 1.70E-01 | *CROT* | 4.48E-01 | *TPMT* | 6.82E-01 | *HMGCR* | 9.93E-01 |
|  |  |  |  |  |  | *PPARG* | 9.93E-01 |
