# Supplementary material for: Drug Metabolizing Enzyme and Transporter Gene Variation, Nicotine Metabolism, Prospective Abstinence, and Cigarette Consumption
Source: PLoS One. 2015 Jul 1;10(7):e0126113. doi: 10.1371/journal.pone.0126113 (PMC4488893; doi:10.1371/journal.pone.0126113)
Supplement: S4 Table — (DOCX) [file pone.0126113.s004.docx]

**S4 Table 4. SNPs Chosen for TaqMan^®^ SNP Genotyping Assay Genotyping.**

| DMET^™^ Probe | SNP | Gene | chr:coor^a^ | Strand | Genomic^b^ | Proteomic^b^ | CEU MAF^c^ | PKFAM MAF | TaqMan^®^ Assay |
| --- | --- | --- | --- | --- | --- | --- | --- | --- | --- |
| AM_12370 | rs1884725 | *XDH* | 2:31425290 | Minus | c.3030T>C | p.Phe1010= | 0.229 | 0.198 | C___3279863_30 |
| AM_13323 | rs3856650 | *CHST13* | 11:12779888 | Plus | c.97+3926T>A |  | 0.398 | 0.412 | C___1935611_10 |
| N/A | rs17329885^d^ | *SLCO1B1* | 12:21219832 | Plus | c.359+922T>C |  | 0.190 | N/A | C__33090816_10 |
| AM_10496 | rs2306283 | *SLCO1B1* | 12:21221005 | Plus | c.388A>G | p.Asn130Asp | 0.398 | 0.404 | C___1901697_20 |
| AM_10659 | rs2297322 | *SLC15A1* | 13:98174182 | Minus | c.350G>A | p.Ser117Asn | 0.093 | 0.103 | AHMSVVN |
| AM_11147 | rs2292954 | *SPG7* | 16:88140624 | Plus | c.1507A>G | p.Thr503Ala | 0.153 | 0.197 | C___3224490_30 |
| AM_11279 | rs1805041 | *CYP4F3* | 19:15624691 | Plus | c.1044G>A | p.Pro348= | 0.259 | 0.232 | C___7496795_10 |
| AM_11280 | rs1805042 | *CYP4F3* | 19:15624721 | Plus | c.1074G>A | p.Val358= | 0.297 | 0.359 | C___7496796_10 |
| AM_11325 | rs1064349 | *CHST8* | 19:38956194 | Plus | c.*386G>A |  | 0.093 | 0.111 | C___7613022_20 |
| N/A | rs28399435^f^ | *CYP2A6* | 19:46048086 | Minus | c.86G>A | p.Ser29Asn | 0.017 | N/A | C__30634234_10 |
| AM_11358 | rs1137115 | *CYP2A6* | 19:46048121 | Minus | c.51A>G^e^ | p.Val17= | 0.246 | 0.214 | C__26681694_20 |
| AM_11364 | rs4803381 | *CYP2A6* | 19:46049184 | Minus | c.-1013A>G^e^ |  | 0.280 | 0.367 | AHMSY6C |
| N/A | rs2835272^d^ | *CBR1* | 21:36374789 | Plus | 3’ flank, T>C |  | 0.106 | N/A | C___2440219_1_ |
| AM_12257 | rs28371725 | *CYP2D6* | 22:40853749 | Minus | c.985+39G>A |  | 0.085 | 0.094 | C__34816116_20 |
| AM_12261 | rs16947 | *CYP2D6* | 22:40853887 | Minus | c.886C>T | p.Arg296Cys | 0.297 | 0.318 | C__27102425_10 |
| AM_12291 | rs1080985 | *CYP2D6* | 22:40858326 | Minus | c.-1589G>C |  | 0.212 | 0.224 | C__32407252_30 |

^a^From NCBI36/hg18. ^b^HGVS annotation from dbSNP. ^c^From Affymetrix DMET^™^ Plus annotation, mean count N=59, including proxies, except rs17329885 and rs2835272, from dbSNP. ^d^rs17329885 is a proxy for rs11045819 (*r*^2^=1, CEU). rs2835272 is a proxy for rs2835265 (*r*^2^=1, CEU). ^e^”A” is minor allele. Reference genome sequence has “T” on + strand, i.e., the minor allele is the genome sequence reference. ^f^One *CYP2A6* SNP not interrogated by the DMET^™^ Plus array underwent genotyping, but failed quality control.
