# Supplementary material for: Drug Metabolizing Enzyme and Transporter Gene Variation, Nicotine Metabolism, Prospective Abstinence, and Cigarette Consumption
Source: PLoS One. 2015 Jul 1;10(7):e0126113. doi: 10.1371/journal.pone.0126113 (PMC4488893; doi:10.1371/journal.pone.0126113)
Supplement: S5 Table — (DOCX) [file pone.0126113.s005.docx]

**S5 Table.** TaqMan^®^ SNP Genotyping Assay Completion Rate, by RCT.

| NCTID | 00326781 | 00322205 | 00301145 | 00087880 | 00086385 | 01621009 | 01621022 | 00332644 |
| --- | --- | --- | --- | --- | --- | --- | --- | --- |
| N | 287 | 332 | 494 | 149 | 177 | 176 | 159 | 696 |
| rs1884725 | 99.7% | 99.4% | 100.0% | 100.0% | 99.4% | 100.0% | 99.4% | 98.8% |
| rs17329885 | 99.3% | 100.0% | 99.4% | 100.0% | 100.0% | 100.0% | 100.0% | 100.0% |
| rs2306283 | 99.7% | 98.5% | 99.0% | 100.0% | 100.0% | 98.9% | 100.0% | 99.3% |
| rs2297322 | 100.0% | 99.7% | 99.6% | 100.0% | 100.0% | 100.0% | 99.4% | 99.7% |
| rs2292954 | 99.7% | 98.8% | 99.8% | 98.0% | **94.9%** | 98.3% | 98.7% | 95.7% |
| rs1805041 | 99.3% | 98.5% | 99.6% | 98.7% | 100.0% | 100.0% | 99.4% | 99.6% |
| rs1805042 | 99.0% | 98.5% | 99.4% | 99.3% | 99.4% | 99.4% | 99.4% | 98.7% |
| rs1064349 | 98.6% | 97.3% | 100.0% | 96.0% | 96.0% | 98.3% | 99.4% | 92.5% |
| rs1137115 | 98.6% | 99.7% | 99.4% | 99.3% | 100.0% | 99.4% | 100.0% | 99.3% |
| rs4803381 | 95.5% | 98.8% | **94.4%** | 100.0% | 100.0% | 100.0% | 99.4% | 98.6% |
| rs2835272 | 99.3% | 100.0% | 99.8% | 100.0% | 100.0% | 100.0% | 100.0% | 100.0% |
| rs28371725 | 99.7% | 98.8% | 99.4% | 98.0% | 98.3% | **92.6%** | 97.5% | **90.8%** |
| rs16947 | 97.9% | 97.0% | 97.4% | 100.0% | 98.9% | 98.3% | 100.0% | **93.1%** |
| rs1080985 | 99.0% | 99.1% | 99.2% | 100.0% | 99.4% | 100.0% | 100.0% | 98.7% |
