# Supplementary material for: Drug Metabolizing Enzyme and Transporter Gene Variation, Nicotine Metabolism, Prospective Abstinence, and Cigarette Consumption
Source: PLoS One. 2015 Jul 1;10(7):e0126113. doi: 10.1371/journal.pone.0126113 (PMC4488893; doi:10.1371/journal.pone.0126113)
Supplement: S6 Table — (DOCX) [file pone.0126113.s006.docx]

**S6 Table. TaqMan^®^ SNP Genotyping Assay HWE Exact *p*-value, by RCT.**

| NCTID | 00326781 | 00322205 | 00301145 | 00087880 | 00086385 | 01621009 | 01621022 | 00332644 |
| --- | --- | --- | --- | --- | --- | --- | --- | --- |
| rs1884725 | 0.196 | 0.758 | 0.813 | 0.673 | 0.073 | 0.824 | 1.000 | **0.007** |
| rs17329885 | 0.468 | 0.828 | 0.750 | 0.777 | 1.000 | 0.261 | 0.772 | 0.073 |
| rs2306283 | 0.626 | 0.484 | 0.581 | 0.507 | 0.640 | 0.107 | 0.865 | 0.877 |
| rs2297322 | 0.586 | 0.240 | 0.785 | 0.728 | 0.397 | 0.324 | 0.399 | 0.830 |
| rs2292954 | 0.072 | 0.723 | 0.316 | **0.002** | **0.017** | 1.000 | 0.747 | **0.001** |
| rs1805041 | 0.316 | 0.592 | 0.403 | 0.302 | 0.653 | 0.839 | 1.000 | 0.261 |
| rs1805042 | 0.889 | 0.724 | 0.773 | 0.468 | 0.139 | 0.735 | **0.010** | 0.289 |
| rs1064349 | 0.291 | 0.807 | 0.657 | **0.012** | **<.0001** | 0.551 | 1.000 | **<.0001** |
| rs1137115 | 0.388 | 0.668 | 0.632 | 1.000 | 0.189 | 0.560 | 0.144 | 0.088 |
| rs4803381 | 0.245 | 0.909 | 1.000 | 0.155 | 1.000 | 0.524 | **0.032** | 0.627 |
| rs2835272 | 0.783 | 1.000 | 0.415 | 1.000 | 0.369 | 0.315 | 0.094 | 0.255 |
| rs28371725 | 0.551 | 0.104 | 0.377 | **0.008** | 0.598 | 0.638 | 0.628 | 0.288 |
| rs16947 | 0.426 | **0.003** | **0.001** | **0.041** | 0.062 | **0.005** | 0.271 | **<.0001** |
| rs1080985 | 0.864 | 0.067 | **0.001** | **0.045** | 0.109 | **0.006** | 0.518 | **0.002** |
