# Supplementary material for: Drug Metabolizing Enzyme and Transporter Gene Variation, Nicotine Metabolism, Prospective Abstinence, and Cigarette Consumption
Source: PLoS One. 2015 Jul 1;10(7):e0126113. doi: 10.1371/journal.pone.0126113 (PMC4488893; doi:10.1371/journal.pone.0126113)
Supplement: S7 Table — (DOCX) [file pone.0126113.s007.docx]

**S7 Table. DMET SNPs and Prospective Abstinence in Eight RCTs.**

| ABS* | N | Gene | SNP | OR | SE | *t* | P>\|t\| | 95% CI | |
| --- | --- | --- | --- | --- | --- | --- | --- | --- | --- |
| EOT | 2497 | *XDH* | rs1884725 | 0.976 | 0.068 | -0.35 | 0.730 | 0.851 | 1.119 |
|  | 2452 | *SLCO1B1* | rs2306283 | 1.053 | 0.065 | 0.84 | 0.402 | 0.933 | 1.190 |
|  | 2465 | *SLCO1B1* | rs17329885 | 1.152 | 0.096 | 1.70 | 0.089 | 0.979 | 1.356 |
|  | 2464 | *SLC15A1* | rs2297322 | 1.097 | 0.108 | 0.95 | 0.344 | 0.905 | 1.330 |
|  | 2455 | *CYP4F3* | rs1805041 | 0.878 | 0.062 | -1.86 | 0.063 | 0.764 | 1.007 |
|  | 2466 | *CYP4F3* | rs1805042 | 1.010 | 0.064 | 0.16 | 0.870 | 0.893 | 1.144 |
|  | 2455 | *CYP2A6* | rs1137115 | 1.033 | 0.072 | 0.47 | 0.638 | 0.902 | 1.183 |
|  | 2487 | *CYP2A6* | rs4803381 | 1.070 | 0.069 | 1.06 | 0.288 | 0.944 | 1.214 |
|  | 2467 | *CBR1* | rs2835272 | 0.858 | 0.081 | -1.62 | 0.105 | 0.714 | 1.033 |
|  | 2375 | *CYP2D6* | rs28371725 | 0.864 | 0.090 | -1.41 | 0.158 | 0.705 | 1.059 |
|  |  |  |  |  |  |  |  |  |  |
| 6MO | 2497 | *XDH* | rs1884725 | 1.003 | 0.074 | 0.04 | 0.968 | 0.868 | 1.159 |
|  | 2452 | *SLCO1B1* | rs2306283 | 1.026 | 0.067 | 0.40 | 0.691 | 0.903 | 1.166 |
|  | 2465 | *SLCO1B1* | rs17329885 | 1.090 | 0.095 | 0.99 | 0.323 | 0.919 | 1.292 |
|  | 2464 | *SLC15A1* | rs2297322 | 1.157 | 0.118 | 1.43 | 0.153 | 0.947 | 1.414 |
|  | 2455 | *CYP4F3* | rs1805041 | 0.957 | 0.071 | -0.60 | 0.550 | 0.828 | 1.106 |
|  | 2466 | *CYP4F3* | rs1805042 | 1.062 | 0.070 | 0.90 | 0.367 | 0.932 | 1.209 |
|  | 2455 | *CYP2A6* | rs1137115 | 0.994 | 0.072 | -0.08 | 0.938 | 0.863 | 1.146 |
|  | 2486 | *CYP2A6* | rs4803381 | 1.022 | 0.069 | 0.33 | 0.743 | 0.896 | 1.166 |
|  | 2467 | *CBR1* | rs2835272 | 1.019 | 0.099 | 0.19 | 0.850 | 0.841 | 1.233 |
|  | 2375 | *CYP2D6* | rs28371725 | 0.862 | 0.095 | -1.34 | 0.179 | 0.694 | 1.071 |

*EOT, end of treatment. 6MO, six months.
