# Supplementary material for: Drug Metabolizing Enzyme and Transporter Gene Variation, Nicotine Metabolism, Prospective Abstinence, and Cigarette Consumption
Source: PLoS One. 2015 Jul 1;10(7):e0126113. doi: 10.1371/journal.pone.0126113 (PMC4488893; doi:10.1371/journal.pone.0126113)
Supplement: S8 Table — (DOCX) [file pone.0126113.s008.docx]

**S8 Table. Power^a^ to Detect SNP Odds Ratios, Six Month Abstinence.**

| SNP | MAF^b^ | 1.05 | 1.10 | 1.20 | 1.40 |
| --- | --- | --- | --- | --- | --- |
| rs1884725 | 0.198 | 0.0989 | 0.2450 | 0.6871 | 0.9960 |
| rs2306283 | 0.404 | 0.1242 | 0.3404 | 0.8429 | 0.9998 |
| rs17329885 | 0.190^c^ | 0.0974 | 0.2390 | 0.6741 | 0.9947 |
| rs2297322 | 0.103 | 0.0783 | 0.1630 | 0.4688 | 0.9437 |
| rs1805041 | 0.232 | 0.1049 | 0.2684 | 0.7343 | 0.9979 |
| rs1805042 | 0.359 | 0.1210 | 0.3288 | 0.8289 | 0.9997 |
| rs1137115 | 0.214 | 0.1018 | 0.2564 | 0.7109 | 0.9969 |
| rs4803381 | 0.367 | 0.1213 | 0.3298 | 0.8301 | 0.9997 |
| rs2835272 | 0.106^c^ | 0.0790 | 0.1659 | 0.4782 | 0.9484 |
| rs28371725 | 0.094 | 0.0761 | 0.1540 | 0.2803 | 0.9265 |

^a^Using: SNP minor allele frequencies from PKTWIN and SMOFAM data, or from Utah residents with ancestry from northern and western Europe (CEU) [[1](#_ENREF_1)]; an additive model; the intent-to-treat six month abstinent and relapsed participant counts from eight clinical trials to define the case:control ratio (1:2.12); the mean six month abstinence rate from eighty placebo arms (13.8%) to define baseline abstinence rates [[2](#_ENREF_2)]; the clinical trial sample size tested with the individual DMET SNP; and a two sided test. ^b^Minor Allele Frequency of PKTWIN and SMOFAM. ^c^CEU MAF.

**References**

1. Coordinators NR (2014) Database resources of the National Center for Biotechnology Information. Nucleic Acids Res 42: D7-17.
2. Clinical Practice Guideline Treating Tobacco U, Dependence Update Panel L and Staff (2008) A clinical practice guideline for treating tobacco use and dependence: 2008 update. A U.S. Public Health Service report. American journal of preventive medicine 35: 158-176.
