# Supplementary material for: Drug Metabolizing Enzyme and Transporter Gene Variation, Nicotine Metabolism, Prospective Abstinence, and Cigarette Consumption
Source: PLoS One. 2015 Jul 1;10(7):e0126113. doi: 10.1371/journal.pone.0126113 (PMC4488893; doi:10.1371/journal.pone.0126113)
Supplement: S9 Table — (DOCX) [file pone.0126113.s009.docx]

**S9 Table. rs4803381 and rs1137115 and Measures of Nicotine Dependence.**

| rs4803381 | N | CPD | CPD (FTND) | TTFC | FTND |
| --- | --- | --- | --- | --- | --- |
| CC | 960 | 22.27 (9.18) | 1.38 (0.78) | 2.00 (0.92) | 5.29 (2.18) |
| TC | 1156 | 21.53 (8.80) | 1.34 (0.76) | 1.99 (0.90) | 5.23 (2.23) |
| TT | 302 | 20.22 (8.62) | 1.22 (0.76) | 2.01 (0.90) | 5.12 (2.11) |
| Total^a^ | 2418 | 21.66 (8.95) | 1.34 (0.77) | 1.99 (0.91) | 5.25 (2.19) |
| rs1137115 |  |  |  |  |  |
| CC | 1356 | 22.00 (8.98) | 1.36 (0.77) | 1.99 (0.90) | 5.25 (2.21) |
| CT | 898 | 21.48 (9.07) | 1.33 (0.77) | 1.99 (0.90) | 5.25 (2.21) |
| TT | 164 | 19.78 (7.74) | 1.21 (0.71) | 2.02 (0.86) | 5.06 (2.06) |
| Total^a^ | 2418 | 21.66 (8.95) | 1.34 (0.77) | 1.99 (0.91) | 5.25 (2.19) |

^a^N=2,418 individuals with both rs4803381 and rs1137115 genotypes.
