# Supplementary material for: Drug Metabolizing Enzyme and Transporter Gene Variation, Nicotine Metabolism, Prospective Abstinence, and Cigarette Consumption
Source: PLoS One. 2015 Jul 1;10(7):e0126113. doi: 10.1371/journal.pone.0126113 (PMC4488893; doi:10.1371/journal.pone.0126113)
Supplement: S10 Table — (DOCX) [file pone.0126113.s010.docx]

**S10 Table. rs4803381 and Abstinence by Pharmacotherapy Randomization.**

| ABS^a^ | PRG^b^ | N | OR | LCI | UCI | SE | *t* | P |
| --- | --- | --- | --- | --- | --- | --- | --- | --- |
| EOT | BUP | 557 | 0.907 | 0.687 | 1.198 | 0.129 | -0.69 | 0.493 |
|  | NRT+BUP | 326 | 1.024 | 0.714 | 1.469 | 0.189 | 0.13 | 0.898 |
|  | PLA | 451 | 0.989 | 0.713 | 1.373 | 0.165 | -0.06 | 0.948 |
|  | VAR | 469 | 1.147 | 0.871 | 1.511 | 0.161 | 0.98 | 0.328 |
|  | NRT | 684 | 1.215 | 0.962 | 1.535 | 0.145 | 1.63 | 0.103 |
|  | *NRT patch* | *339* | *1.348* | *0.961* | *1.890* | *0.232* | *1.73* | *0.084* |
|  | NRT lozenge | 191 | 1.194 | 0.771 | 1.848 | 0.266 | 0.79 | 0.427 |
|  | NRT spray | 154 | 0.854 | 0.458 | 1.594 | 0.272 | -0.50 | 0.620 |
|  |  |  |  |  |  |  |  |  |
| 6MO | BUP | 557 | 0.896 | 0.662 | 1.211 | 0.138 | -0.72 | 0.474 |
|  | **CBUP+NRT** | **66** | **0.291** | **0.101** | **0.842** | **0.158** | **-2.28** | **0.023** |
|  | CNRT+BUP | 99 | 0.660 | 0.324 | 1.348 | 0.240 | -1.14 | 0.255 |
|  | NFT | 161 | 0.650 | 0.321 | 1.315 | 0.234 | -1.20 | 0.231 |
|  | PLA | 451 | 1.131 | 0.790 | 1.618 | 0.207 | 0.67 | 0.502 |
|  | VAR | 469 | 0.920 | 0.699 | 1.210 | 0.129 | -0.60 | 0.550 |
|  | *NRT* | *684* | *1.252* | *0.976* | *1.606* | *0.158* | *1.77* | *0.077* |
|  | *NRT patch* | *339* | *1.386* | *0.973* | *1.975* | *0.250* | *1.81* | *0.071* |
|  | NRT lozenge | 191 | 1.278 | 0.813 | 2.008 | 0.295 | 1.06 | 0.287 |
|  | NRT spray | 154 | 0.903 | 0.462 | 1.766 | 0.309 | -0.30 | 0.766 |

^a^EOT, end of treatment. 6MO, six months. ^b^BUP=bupropion; NRT+BUP=nicotine replacement therapy (NRT) and BUP to EOT (12 weeks); then individuals were randomized to CBUP+NRT=chronic BUP and NRT, CNRT+BUP=chronic NRT and BUP, or NFT=no further treatment, from EOT to 12 months; PLA=placebo; VAR=varenicline; NRT= randomized to NRT patch, NRT lozenge, or to NRT spray.
