# Supplementary material for: Drug Metabolizing Enzyme and Transporter Gene Variation, Nicotine Metabolism, Prospective Abstinence, and Cigarette Consumption
Source: PLoS One. 2015 Jul 1;10(7):e0126113. doi: 10.1371/journal.pone.0126113 (PMC4488893; doi:10.1371/journal.pone.0126113)
Supplement: S11 Table — (DOCX) [file pone.0126113.s011.docx]

**S11 Table. TRICL rs1137115 Lung Cancer Results.**

| Study sample | Odds Ratio | 95% CI | *P* |
| --- | --- | --- | --- |
| Meta-analysis^a^ | 0.9199 | 0.8721-0.9705 | 0.0022 |
| Heidelberg^b^ | 1.0110 | 0.8113-1.2599 | 0.9223 |
| ICR^c^ | 0.8960 | 0.8189-0.9803 | 0.0167 |
| NCI^d^ | 0.9105 | 0.8396-0.9873 | 0.0234 |
| Toronto^e^ | 0.9505 | 0.6948-1.3003 | 0.7511 |

^a^Fixed effects and random effects models of four studies of lung cancer gave the same results. Cochran’s *Q* *p*-value was 0.745 and *I*^2^ was 0.000. ^b^The Heidelberg GWAS included Heidelberg lung cancer study cases [[1](#_ENREF_1)] and Heidelberg-European Prospective Investigation into Cancer and Nutrition controls [[2](#_ENREF_2)]. ^c^The Institute for Cancer Research replication GWAS included cases collected through the GEenetic Lung CAncer Predisposition Study [[3](#_ENREF_3)] and controls collected through the National Study of Colorectal Cancer Genetics [[4](#_ENREF_4)]. ^d^The NCI GWAS consisted of case:control samples from: the Environment and Genetics in Lung cancer Etiology study [[5](#_ENREF_5)]; the α-Tocopherol, β-Carotene Cancer Prevention Study [[6](#_ENREF_6)]; the Prostate, Lung, Colon, Ovary Screening Trial [[7](#_ENREF_7)]; and the Cancer Prevention Study II Nutrition Cohort [[8](#_ENREF_8)]. ^e^The Toronto GWAS study recruited cases from hospitals in the University of Toronto network, and randomly selected age and sex-matched controls from family medicine clinics [[9](#_ENREF_9)].

**References**

1. Boeing H, Wahrendorf J and Becker N (1999) EPIC-Germany--A source for studies into diet and risk of chronic diseases. European Investigation into Cancer and Nutrition. Annals of nutrition & metabolism 43: 195-204.
2. Dally H, Edler L, Jager B, Schmezer P, Spiegelhalder B, Dienemann H, et al. (2003) The CYP3A4*1B allele increases risk for small cell lung cancer: effect of gender and smoking dose. Pharmacogenetics 13: 607-618.
3. Eisen T, Matakidou A, Houlston R and Consortium G (2008) Identification of low penetrance alleles for lung cancer: the GEnetic Lung CAncer Predisposition Study (GELCAPS). BMC cancer 8: 244.
4. Penegar S, Wood W, Lubbe S, Chandler I, Broderick P, Papaemmanuil E, et al. (2007) National study of colorectal cancer genetics. British journal of cancer 97: 1305-1309.
5. Landi MT, Consonni D, Rotunno M, Bergen AW, Goldstein AM, Lubin JH, et al. (2008) Environment And Genetics in Lung cancer Etiology (EAGLE) study: an integrative population-based case-control study of lung cancer. BMC public health 8: 203.
6. (1994) The alpha-tocopherol, beta-carotene lung cancer prevention study: design, methods, participant characteristics, and compliance. The ATBC Cancer Prevention Study Group. Annals of epidemiology 4: 1-10.
7. Hayes RB, Sigurdson A, Moore L, Peters U, Huang WY, Pinsky P, et al. (2005) Methods for etiologic and early marker investigations in the PLCO trial. Mutation research 592: 147-154.
8. Calle EE, Rodriguez C, Jacobs EJ, Almon ML, Chao A, McCullough ML, et al. (2002) The American Cancer Society Cancer Prevention Study II Nutrition Cohort: rationale, study design, and baseline characteristics. Cancer 94: 2490-2501.
9. Wang Y, McKay JD, Rafnar T, Wang Z, Timofeeva MN, Broderick P, et al. (2014) Rare variants of large effect in BRCA2 and CHEK2 affect risk of lung cancer. Nature genetics 46: 736-741.
