# Supplementary material for: Drug Metabolizing Enzyme and Transporter Gene Variation, Nicotine Metabolism, Prospective Abstinence, and Cigarette Consumption
Source: PLoS One. 2015 Jul 1;10(7):e0126113. doi: 10.1371/journal.pone.0126113 (PMC4488893; doi:10.1371/journal.pone.0126113)
Supplement: S1 File — (DOCX) [file pone.0126113.s012.docx]

**S1 File. Discovery (Stage I) and Validation (Stage II) Analyses.**

**Affymetrix DMET^™^ Plus Array genotyping (Stage I)**

563 DNA samples from 554 individuals (338 PKTWIN and 216 SMOFAM individuals, with 9 duplicates) were genotyped using the Affymetrix DMET^™^ Plus Array [[1](#_ENREF_1), [2](#_ENREF_2)]. 561 of 563 DNA samples met concentration specifications (≥40ng/ul by PicoGreen^®^). The genotyping was accomplished in two stages by the Affymetrix Service group. In the first stage, 133 DNA samples from the PKTWIN and SMOFAM datasets were genotyped, along with six genomic DNA samples provided by Affymetrix as control samples. In the second stage, 430 samples and 15 control samples were genotyped. A genotyping completion rate threshold ≥98% was considered passing. Genotype completion pass rates for the two stages were 133 of 133 and 427 of 430 samples with average genotyping completion rates of 0.9992 and 0.9990. Six of six and 14 of 15 control samples passed with average genotyping completion rates of 0.9979 and 0.9977. Average replicate concordance for test and control samples at stages one and two were 0.9997, 0.9998, 0.9993 and 0.9989, respectively. Average concordance of control samples with previously obtained genotypes was 0.9997 and 0.9994 for stages one and two.

**Hierarchical Linear Regression Models**

The HLM model for PKTWIN is defined: Let $Y_{ijk}$ be the NMR for the i-th child in the k-th family where j takes on a unique value for each set of MZ twins and for each other child (i.e., separate values for each DZ twin). Let $X_{ijk}$be the additive or dominantly coded SNP value for person ijk with coefficient B, and let $W_{ijk}$ be a vector of covariates with vector coefficients A. Then the HLM for examining the relationship of the SNP to NMR is as follows:

$$Y_{ijk}= \mu+B_{1}X_{ijk}+AW_{ijk}+a_{1k}+ a_{jk} +\epsilon_{ijk}$$

Here $a_{1k}$ is a random effect for family, $a_{jk}$ is a random effect for sibling group within family (with MZ twins sharing the same value and DZ twins each having a unique value), and $\epsilon_{ijk}$ is a random effect.

The HLM model for SMOFAM is defined: Let $Y_{ijk}$ be the NMR for the i-th person in the k-th family, where j takes on a unique value for each parent and for all siblings. Let $X_{ijk}$be the additive coded SNP value for person ijk with coefficient B, and let $W_{ijk}$ be a vector of covariates with vector coefficients A. Then the HLM for examining the relationship of the SNP to NMR is:

$$Y_{ijk}= \mu+B_{1}X_{ijk}+AW_{ijk}+a_{1k}^{(4)}\left[ M_{i}+\frac{K_{i}}{2} \right]+ a_{2k}^{(4)} \left[ F_{i}+\frac{K_{i}}{2} \right]+a_{ijk}^{(2)}\left[ \frac{K_{i}}{\sqrt{2}} \right]+ c_{jk}^{(3)}+\epsilon_{ijk}$$

Here $M_{i}$ is a dummy variable for mothers, $F_{i}$ for fathers and $K_{i}$ for children. The terms $a_{1k}^{(4)}$, $a_{2k}^{(4)}$, $a_{ijk}^{(2)}$, $c_{jk}^{(3)}$, and $\epsilon_{ijk}$ are random effects, such that the term $a_{1k}^{(4)}\left[ M_{i}+\frac{K_{i}}{2} \right]+ a_{2k}^{(4)} \left[ F_{i}+\frac{K_{i}}{2} \right]+a_{ijk}^{(2)}\left[ \frac{K_{i}}{\sqrt{2}} \right]$ represents the additive genetic component with a total variance of $\sigma_{A}^{2}$ whereas Var($c_{jk}^{(3)})$ = $\sigma_{C}^{2}$ and Var($\epsilon_{ijk})= \sigma_{E}^{2}$. Here $a_{1k}^{(4)}$ and $a_{2k}^{(4)}$ induce the required genetic additive genetic covariances between each parent and each child among the children. However, the induced variances for the children are only $\sigma_{A}^{2}/2$ and the remaining variance $\sigma_{A}^{2}/2$ is provided by $a_{ijk}^{(2)}$. The common environmental component $c_{jk}^{(3)}$ is shared among children but unique to each parent. The unique environmental component is represented by $\epsilon_{ijk}$.

**TaqMan^®^ SNP Genotyping Assay Genotyping (Stage II)**

For 14 SNPs, 100 ng of DNA was aliquoted into TaqMan^®^ OpenArray^®^ 384-well Sample Plates and dried down at room temperature overnight. TaqMan^®^ OpenArray^®^ Genotyping master mix was dispensed to the plate wells, the mixture dispensed into the 3,072 through-holes in the TaqMan^®^ OpenArray^®^ Genotyping Plate with an Accufill loader, predesigned or custom TaqMan^®^ SNP Genotyping Assays were used for genotyping (see S4 Table), cycled on a GeneAmp^®^ PCR System 9700 according to the standard protocol, and read on an NT Cycler Instrument. Cluster plots were reviewed manually and additional manual genotype calling was performed if required. ~8% of the DNA samples were included in duplicate to calculate genotype discordance. Genotypes were analyzed using TaqMan^®^ Genotyper Software v1.3. SNP assays for rs3856650 and rs28399435 were dropped from further analysis because of poor clustering and low call rates. The latter assay was designed to interrogate a non-synonymous substitution not interrogated by the DMET^™^ Plus panel. DNA samples with genotype completion rates <75% were dropped from further analysis. We observed a 1.5% replicate DNA sample genotype discordance rate for clinical trial participant DNA genotyped using TaqMan^®^ SNP Genotyping Assays on the OpenArray^®^ Genotyping System before assay and DNA sample removal and a 0.9% discordance rate after assay and DNA sample removal.

rs1884725 and rs4803381 were analyzed on a ViiA^™^ 7 Real-Time PCR System using Taqman^®^ SNP Genotyping assays with standard DME Taqman^®^ SNP Genotyping assay conditions. rs1884725 genotyping was performed with a predesigned TaqMan^®^ SNP Genotyping Assay and was unremarkable (see S5 and S6 Tables). The latest human genome assembly (hg38) does not align rs4803381 to the genome, assemblies hg18 and hg19 align rs4803381 5’ of the transcription start sites of both CYP2A6 and CYP2A7, and assemblies hg16 and hg17 align rs4803381 5’ of CYP2A6. Unpublished sequencing data supports the location of rs4803381 5’ of CYP2A6 (unpublished data, personal communication, Zhu and Tyndale), which we confirmed using two genotyping assays interrogating both locations. Working with the Life Technologies Bioinformatics group, two custom assays were designed to genotype rs4803381, which has two possible locations, 5’ of *CYP2A6* and 5’ of *CYP2A7* in the latest genome assembly (hg19). NCBI maps rs4803381 5’ of *CYP2A6* and UCSC identifies the SNP at two locations, 5’ of *CYP2A6* and 5’ of *CYP2A7* in the hgTable browser. Assay AHMSY6C was designed to interrogate rs4803381 at the position 5’ of *CYP2A6*, with the expectation of observing variation, and Assay AHN1XCK was designed to interrogate the homologous position 5’ of *CYP2A7*, with the expectation that AHN1XCK would not reveal variation. AHMSY6C was used to genotype 356 PKFAM (PKTWIN and SMOFAM) DNA and gave a 96.3% completion rate. There was a 97.1% concordance rate for the called genotypes when compared to the DMET^™^ Plus Array probe AM_11364 genotype calls. Of the 10 DNA samples with genotypes discordant between AHMSY6C and AM_11364, eight gave GG with AHMSY6C and AG with AM_11364, while two gave AG with AHMSY6C and GG with AM_11364 (see below). Therefore, Assay AHMSY6C undercounts the rs4803381 minor allele relative to Probe AM_11364.

rs4803381 genotypes using two different genotyping platforms.

|  | DMET^™^ Plus AM_11364, Stage I | | | |
| --- | --- | --- | --- | --- |
| TaqMan^®^ AHMSY6C on ViiA^™^7, Stage II |  | **AA** | **AG** | **GG** |
|  | **AA** | 28 | 0 | 0 |
|  | **AG** | 0 | 157 | 2 |
|  | **GG** | 0 | 8 | 148 |

The TaqMan^®^ SNP Genotyping Assay AHN1XCK gave all homozygous called genotypes for 165 of 168 DNA samples successfully genotyped (98.2% completion rate), confirming our expectation that rs4803381 is found 5’ of *CYP2A6*. AHMSY6C was then used to genotype DNA samples from the eight RCTs (see S5 and S6 Tables). After exclusion of Assays and DNA samples with poor performance, TaqMan^®^ SNP Genotyping Assay completion rates are ≥95% in 107 of 112 clinical trial-by-SNP cells; five clinical trial-by-SNP cells have genotyping rates ≥90% and <95% (S5 Table). Hardy-Weinberg Equilibrium (HWE) exact *p-*values are <0.05 in 17 of 112 clinical trial-by-SNP cells (S6 Table). rs2292954, rs1064349, rs16947 and rs1080985 exhibit three, three, five and four clinical trial-by-SNP cells with HWE *p*-values <0.05, respectively, and were excluded from further analyses, leaving two clinical trial-by-SNP cell HWE *p*-values <0.05 in 89 remaining clinical trial-by-SNP cells.

**DMET SNPs and Commercial Genotyping Assay Metrics**

We used a commercially-available analysis platform to genotype 1936 variants in 231 DMET genes selected in an industry survey, with specific polymorphisms chosen by committee; 32 genes chosen by all seven companies were defined as Core ADME genes, while the remaining genes were still considered to represent DMET candidates [[1-3](#_ENREF_1)]. We observed 99.5% concordance between DMET™ and TaqMan^®^ SNP Genotyping Assay genotypes after quality filtering; some multiplex platforms exhibit higher concordance rates [[4](#_ENREF_4)]. The average coverage of the DMET^™^ Plus array to common tagSNPs is lower than all genome-wide platforms examined, but has comparable coverage (20%-25%) for *CYP2A6* [[5](#_ENREF_5)]. *CYP2A6* is a PharmADME Core gene, and a Pharmacogenetics Research Network (PGRN) PharmGKB Very Important Pharmacogene (VIP) gene [[6](#_ENREF_6)]. The purpose of genotyping PharmADME or PGRN PharmGKB VIP gene variants included on the DMET^™^ Plus Array [[1](#_ENREF_1)] and on the Illumina ADME panel [[7](#_ENREF_7)] is to interrogate putatively functional variation, not to interrogate directly, or through linkage disequilibrium, all DMET gene variation. SNPs declared significant in the discovery stage of this analysis and included in the validation stage included SNPs within cytochrome P450 genes. 50% of the TaqMan^®^ SNP Genotyping Assay exclusions in the validation phase of this study were *CYP2A6* and *CYP2D6* SNPs. The Illumina ADME panel for PharmaADME Core gene variant analysis exhibits less than optimal completion rates for SNPs at these two genes and two *CYP2D6* SNPs exhibited significant deviation from HWE in an analysis of a European American ancestry patient population including rs1080985 [[7](#_ENREF_7)]. rs1080985 exhibited significant deviation from HWE in the validation stage of this study.

***Post-hoc* Power to detect effects of DMET SNPs on NMR and prospective abstinence**

The power to detect proportions of variance (0.5%, 1.0%, 2.0%, 4.0%) of the residualized transformed NMR was 42%, 70%, 94% and 100% with the sample size tested in analysis of rs1137115 (N=614). The power to detect genetic odds ratios (1.05, 1.10, 1.20, 1.40) on six month abstinence in the sample of 2499 individuals for nine SNPs ranged from 7.6-12.4%, 16.4-34.0%, 28.3-84.3%, to 94.4-100%, respectively (S6 Table).

**References**

1. Burmester JK, Sedova M, Shapero MH and Mansfield E (2010) DMET microarray technology for pharmacogenomics-based personalized medicine. Methods Mol Biol 632: 99-124.
2. Deeken J (2009) The Affymetrix DMET platform and pharmacogenetics in drug development. Curr Opin Mol Ther 11: 260-268.
3. Sissung TM, English BC, Venzon D, Figg WD and Deeken JF (2010) Clinical pharmacology and pharmacogenetics in a genomics era: the DMET platform. Pharmacogenomics 11: 89-103.
4. Fernandez CA, Smith C, Yang W, Lorier R, Crews KR, Kornegay N, et al. (2012) Concordance of DMET plus genotyping results with those of orthogonal genotyping methods. Clinical pharmacology and therapeutics 92: 360-365.
5. Gamazon ER, Skol AD and Perera MA (2012) The limits of genome-wide methods for pharmacogenomic testing. Pharmacogenetics and genomics 22: 261-272.
6. McDonagh EM, Wassenaar C, David SP, Tyndale RF, Altman RB, Whirl-Carrillo M, et al. (2012) PharmGKB summary: very important pharmacogene information for cytochrome P-450, family 2, subfamily A, polypeptide 6. Pharmacogenetics and genomics 22: 695-708.
7. Oetjens MT, Denny JC, Ritchie MD, Gillani NB, Richardson DM, Restrepo NA, et al. (2013) Assessment of a pharmacogenomic marker panel in a polypharmacy population identified from electronic medical records. Pharmacogenomics 14: 735-744.
