# Supplementary material for: Drug Metabolizing Enzyme and Transporter Gene Variation, Nicotine Metabolism, Prospective Abstinence, and Cigarette Consumption
Source: PLoS One. 2015 Jul 1;10(7):e0126113. doi: 10.1371/journal.pone.0126113 (PMC4488893; doi:10.1371/journal.pone.0126113)
Supplement: S2 File — (DOCX) [file pone.0126113.s013.docx]

**Prior CYP2A6 Associations with NMR, Abstinence, CPD and Lung Cancer Risk.**

In the twin-based laboratory-based study used in our Stage I analysis, variation at *CYP2A6* was previously associated with the rate of nicotine metabolism, and additive genetic influence remained after accounting for six *CYP2A6* variants [[1](#_ENREF_1), [2](#_ENREF_2)]. In analyses of 148 *ad libitum* smokers, levels of nicotine and of *trans*-3’-hydroxycotinine, and multiple ratios of nicotine metabolites, including the NMR, are significantly correlated with *CYP2A6* genotypes [[3](#_ENREF_3)], grouped by predicted CYP2A6 activity [[1](#_ENREF_1), [4-12](#_ENREF_4)]. The “CYP2A6 activity model” modeled the association between seven *CYP2A6* variants, sex and smoking status with several metabolic ratios in a laboratory study of nicotine metabolism [[13](#_ENREF_13)] in European American ancestry individuals [[14](#_ENREF_14)]. The CYP2A6 activity model accounted for 47.6% of the variance of the laboratory study-based NMR 240 minutes after labeled compound administration [[13](#_ENREF_13)].

In retrospective analyses of therapy efficacy in four clinical trials [[15-17](#_ENREF_15)], European American or African American ancestry treatment-seeking smokers with a NMR in the lowest quartile [[18-20](#_ENREF_18)], or with *CYP2A6* variants associated with low enzyme activity [[21](#_ENREF_21), [22](#_ENREF_22)], and randomized to nicotine replacement therapy or to placebo pharmacotherapy, were significantly more likely to remain abstinent than those in the upper three quartiles of the NMR or with *CYP2A6* variants associated with normal activity. In retrospective analysis of clinical trial participants [[23](#_ENREF_23)], and stratifying individuals by the 25^th^ percentile of the CYP2A6 activity model, three hypotheses of association of CYP2A6 activity and relapse risk were confirmed: a) slow-metabolizer status decreases risk; b) slow-metabolizer status and randomization to active pharmacotherapy each decrease risk; and c) slow-metabolizer status and randomization to nicotine replacement therapy (NRT) each decrease risk [[24](#_ENREF_24)]. In a prospective NMR-stratified smoking cessation trial including both African American and European American ancestry participants randomized to nicotine patch, varenicline and placebo therapies, the NMR was significantly associated with abstinence at end of treatment and at six months in interaction with treatment, with normal metabolizers more likely to quit than slow metabolizers when randomized to varenicline compared to nicotine patch [[25](#_ENREF_25)].

In three samples of smokers of two different ancestries drawn from two cohort studies totaling 2,401 individuals [[14](#_ENREF_14), [26](#_ENREF_26)], European American ancestry smokers with loss of function *CYP2A6* haplotypes exhibited significantly reduced CPD [[27](#_ENREF_27)]; and, among smokers with FTND/FTCD scores ≥4, the CYP2A6 activity model was significantly associated with continuous CPD (*P*=0.005 European American ancestry, *P*=0.027 African American ancestry) [[27](#_ENREF_27)]. In 860 European smokers included in a lung cancer case:control study, *CYP2A6* normal metabolizers smoked significantly more CPD than reduced metabolizers (27.7 vs 24.7 CPD, *P*=0.01) [[28](#_ENREF_28)]. In meta-GWAS analyses of European smokers, *CYP2A6* SNPs rs1801272 (c.479T>A/p.L160H) and rs4105144 (-2729A>G) are significantly associated with CPD [effect sizes of 0.68 CPD (N=66,380, *P*=1.1E-4) and 0.39 CPD (N=83,317, *P*=2.2E-12)] [[29](#_ENREF_29)]. Bloom *et al* estimate that rs1137115 is in strong linkage disequilibrium (*D*’≥0.95) with rs4105144 [[27](#_ENREF_27)].

In an Italian case:control study (N=4,221) [[30](#_ENREF_30)], rs1801272 was significantly associated with lung cancer (*P*=0.026) [[31](#_ENREF_31)]. In the lighter-smoking stratum from a lung cancer case:control study of European American ancestry indivdiuals, *CYP2A6* metabolizer status, defined by haplotype, was significantly associated with lung cancer (*P*=0.036) [[32](#_ENREF_32)]. In a meta-GWAS analysis (N=42,289), rs4105144 was significantly associated with lung cancer (*P*=0.04) [[29](#_ENREF_29)]. In 845 European current smokers in a lung cancer case:control study [[28](#_ENREF_28)], rs4105144 is associated with increased serum cotinine (*P*_trend_=0.0001) [[28](#_ENREF_28)], a function of decreased cotinine clearance [[33](#_ENREF_33)], and with decreased lung cancer risk (*P*=0.01, adjusted for cotinine) [[28](#_ENREF_28)].

**References**

1. Benowitz NL, Swan GE, Jacob P, 3rd, Lessov-Schlaggar CN and Tyndale RF (2006) CYP2A6 genotype and the metabolism and disposition kinetics of nicotine. Clinical pharmacology and therapeutics 80: 457-467.
2. Swan GE, Lessov-Schlaggar CN, Bergen AW, He Y, Tyndale RF and Benowitz NL (2009) Genetic and environmental influences on the ratio of 3'hydroxycotinine to cotinine in plasma and urine. Pharmacogenetics and genomics 19: 388-398.
3. Malaiyandi V, Goodz SD, Sellers EM and Tyndale RF (2006) CYP2A6 genotype, phenotype, and the use of nicotine metabolites as biomarkers during ad libitum smoking. Cancer epidemiology, biomarkers & prevention : a publication of the American Association for Cancer Research, cosponsored by the American Society of Preventive Oncology 15: 1812-1819.
4. Pitarque M, von Richter O, Oke B, Berkkan H, Oscarson M and Ingelman-Sundberg M (2001) Identification of a single nucleotide polymorphism in the TATA box of the CYP2A6 gene: impairment of its promoter activity. Biochemical and biophysical research communications 284: 455-460.
5. Yamano S, Tatsuno J and Gonzalez FJ (1990) The CYP2A3 gene product catalyzes coumarin 7-hydroxylation in human liver microsomes. Biochemistry 29: 1322-1329.
6. Oscarson M, McLellan RA, Gullsten H, Yue QY, Lang MA, Bernal ML, et al. (1999) Characterisation and PCR-based detection of a CYP2A6 gene deletion found at a high frequency in a Chinese population. FEBS letters 448: 105-110.
7. Oscarson M, McLellan RA, Gullsten H, Agundez JA, Benitez J, Rautio A, et al. (1999) Identification and characterisation of novel polymorphisms in the CYP2A locus: implications for nicotine metabolism. FEBS letters 460: 321-327.
8. Ariyoshi N, Sawamura Y and Kamataki T (2001) A novel single nucleotide polymorphism altering stability and activity of CYP2a6. Biochemical and biophysical research communications 281: 810-814.
9. Dempsey D, Tutka P, Jacob P, 3rd, Allen F, Schoedel K, Tyndale RF, et al. (2004) Nicotine metabolite ratio as an index of cytochrome P450 2A6 metabolic activity. Clinical pharmacology and therapeutics 76: 64-72.
10. Swan GE, Benowitz NL, Jacob P, 3rd, Lessov CN, Tyndale RF, Wilhelmsen K, et al. (2004) Pharmacogenetics of nicotine metabolism in twins: methods and procedures. Twin Res 7: 435-448.
11. Xu C, Rao YS, Xu B, Hoffmann E, Jones J, Sellers EM, et al. (2002) An in vivo pilot study characterizing the new CYP2A6*7, *8, and *10 alleles. Biochemical and biophysical research communications 290: 318-324.
12. Oscarson M, McLellan RA, Asp V, Ledesma M, Bernal Ruiz ML, Sinues B, et al. (2002) Characterization of a novel CYP2A7/CYP2A6 hybrid allele (CYP2A6*12) that causes reduced CYP2A6 activity. Human mutation 20: 275-283.
13. Bloom J, Hinrichs AL, Wang JC, von Weymarn LB, Kharasch ED, Bierut LJ, et al. (2011) The contribution of common CYP2A6 alleles to variation in nicotine metabolism among European-Americans. Pharmacogenetics and genomics 21: 403-416.
14. Bierut LJ, Madden PA, Breslau N, Johnson EO, Hatsukami D, Pomerleau OF, et al. (2007) Novel genes identified in a high-density genome wide association study for nicotine dependence. Human molecular genetics 16: 24-35.
15. Lerman C, Jepson C, Wileyto EP, Epstein LH, Rukstalis M, Patterson F, et al. (2006) Role of functional genetic variation in the dopamine D2 receptor (DRD2) in response to bupropion and nicotine replacement therapy for tobacco dependence: results of two randomized clinical trials. Neuropsychopharmacology : official publication of the American College of Neuropsychopharmacology 31: 231-242.
16. Schnoll RA, Patterson F, Wileyto EP, Heitjan DF, Shields AE, Asch DA, et al. (2010) Effectiveness of extended-duration transdermal nicotine therapy: a randomized trial. Annals of internal medicine 152: 144-151.
17. Ahluwalia JS, Okuyemi K, Nollen N, Choi WS, Kaur H, Pulvers K, et al. (2006) The effects of nicotine gum and counseling among African American light smokers: a 2 x 2 factorial design. Addiction 101: 883-891.
18. Lerman C, Tyndale R, Patterson F, Wileyto EP, Shields PG, Pinto A, et al. (2006) Nicotine metabolite ratio predicts efficacy of transdermal nicotine for smoking cessation. Clinical pharmacology and therapeutics 79: 600-608.
19. Patterson F, Schnoll RA, Wileyto EP, Pinto A, Epstein LH, Shields PG, et al. (2008) Toward personalized therapy for smoking cessation: a randomized placebo-controlled trial of bupropion. Clinical pharmacology and therapeutics 84: 320-325.
20. Schnoll RA, Patterson F, Wileyto EP, Tyndale RF, Benowitz N and Lerman C (2009) Nicotine metabolic rate predicts successful smoking cessation with transdermal nicotine: a validation study. Pharmacology, biochemistry, and behavior 92: 6-11.
21. Lerman C, Jepson C, Wileyto EP, Patterson F, Schnoll R, Mroziewicz M, et al. (2010) Genetic variation in nicotine metabolism predicts the efficacy of extended-duration transdermal nicotine therapy. Clinical pharmacology and therapeutics 87: 553-557.
22. Ho MK, Mwenifumbo JC, Al Koudsi N, Okuyemi KS, Ahluwalia JS, Benowitz NL, et al. (2009) Association of nicotine metabolite ratio and CYP2A6 genotype with smoking cessation treatment in African-American light smokers. Clinical pharmacology and therapeutics 85: 635-643.
23. Piper ME, Smith SS, Schlam TR, Fiore MC, Jorenby DE, Fraser D, et al. (2009) A randomized placebo-controlled clinical trial of 5 smoking cessation pharmacotherapies. Archives of general psychiatry 66: 1253-1262.
24. Chen LS, Bloom AJ, Baker TB, Smith SS, Piper ME, Martinez M, et al. (2014) Pharmacotherapy effects on smoking cessation vary with nicotine metabolism gene (CYP2A6). Addiction 109: 128-137.
25. Lerman C, Schnoll RA, Hawk LW, Cinciripinni P, George TP, Wileyto EP, et al. (in press ) A Randomized Placebo-controlled Trial to Test a Genetically-informed Biomarker For Personalizing Treatment for Tobacco Dependence. Lancet Respir Med.
26. Saccone SF, Pergadia ML, Loukola A, Broms U, Montgomery GW, Wang JC, et al. (2007) Genetic linkage to chromosome 22q12 for a heavy-smoking quantitative trait in two independent samples. American journal of human genetics 80: 856-866.
27. Bloom AJ, Harari O, Martinez M, Madden PA, Martin NG, Montgomery GW, et al. (2012) Use of a predictive model derived from in vivo endophenotype measurements to demonstrate associations with a complex locus, CYP2A6. Human molecular genetics 21: 3050-3062.
28. Timofeeva MN, McKay JD, Smith GD, Johansson M, Byrnes GB, Chabrier A, et al. (2011) Genetic polymorphisms in 15q25 and 19q13 loci, cotinine levels, and risk of lung cancer in EPIC. Cancer epidemiology, biomarkers & prevention : a publication of the American Association for Cancer Research, cosponsored by the American Society of Preventive Oncology 20: 2250-2261.
29. Thorgeirsson TE, Gudbjartsson DF, Surakka I, Vink JM, Amin N, Geller F, et al. (2010) Sequence variants at CHRNB3-CHRNA6 and CYP2A6 affect smoking behavior. Nature genetics 42: 448-453.
30. Landi MT, Consonni D, Rotunno M, Bergen AW, Goldstein AM, Lubin JH, et al. (2008) Environment And Genetics in Lung cancer Etiology (EAGLE) study: an integrative population-based case-control study of lung cancer. BMC public health 8: 203.
31. Rotunno M, Yu K, Lubin JH, Consonni D, Pesatori AC, Goldstein AM, et al. (2009) Phase I metabolic genes and risk of lung cancer: multiple polymorphisms and mRNA expression. PloS one 4: e5652.
32. Wassenaar CA, Dong Q, Wei Q, Amos CI, Spitz MR and Tyndale RF (2011) Relationship between CYP2A6 and CHRNA5-CHRNA3-CHRNB4 variation and smoking behaviors and lung cancer risk. J Natl Cancer Inst 103: 1342-1346.
33. Zhu AZ, Renner CC, Hatsukami DK, Swan GE, Lerman C, Benowitz NL, et al. (2013) The ability of plasma cotinine to predict nicotine and carcinogen exposure is altered by differences in CYP2A6: the influence of genetics, race, and sex. Cancer epidemiology, biomarkers & prevention : a publication of the American Association for Cancer Research, cosponsored by the American Society of Preventive Oncology 22: 708-718.
